# Supplementary material for: Glycan-Induced Protein Dynamics in Human Norovirus P Dimers Depend on Virus Strain and Deamidation Status
Source: Molecules. 2021 Apr 7;26(8):2125. doi: 10.3390/molecules26082125 (PMC8067865; doi:10.3390/molecules26082125)
Supplement: Supplementary file 1 [file molecules-26-02125-s001.pdf]

Supplemental figures and tables

Glycan-induced protein dynamics in human norovirus P dimers depend on virus strain and deamidation status

Jasmin Dülfer [1], Hao Yan [1], Maxim N Brodmerkel [2], Robert Creutznacher [3], Alvaro Mallagaray [3], Thomas Peters [3], Carl Caleman [4,5], Erik G Marklund [2] and Charlotte Uetrecht [1,6]*

[1] Heinrich Pette Institute, Leibniz Institute for Experimental Virology, Hamburg, Germany;
[2] Department of Chemistry – BMC, Uppsala University, Uppsala, Sweden
[3] Institute of Chemistry and Metabolomics, University of Lübeck, Lübeck, Germany
[4] Department of Physics and Astronomy, Uppsala University, Uppsala, Sweden
[5] Center for Free-Electron Laser Science, Deutsches Elektronen-Synchrotron, Hamburg, Germany
[6] European XFEL GmbH, Schenefeld, Germany
* Correspondence: charlotte.uetrecht@xfel.eu

Contents

1 Supporting data 2
1.1 Sequence alignment and homology modeling 2
1.2 Identification of deamidation 4
1.3 Native MS 12
1.4 Analysis of bimodal spectra 14
1.5 Woods Plots 25
1.6 MD simulations 27
2 HDX figures and tables 35
2.1 Peptide coverage maps 35
2.2 HDX summary tables 40
2.3 MS-Viewer search keys 45
2.4 Deuterium uptake plots 46

1 Supporting data

1.1 Sequence alignment and homology modeling

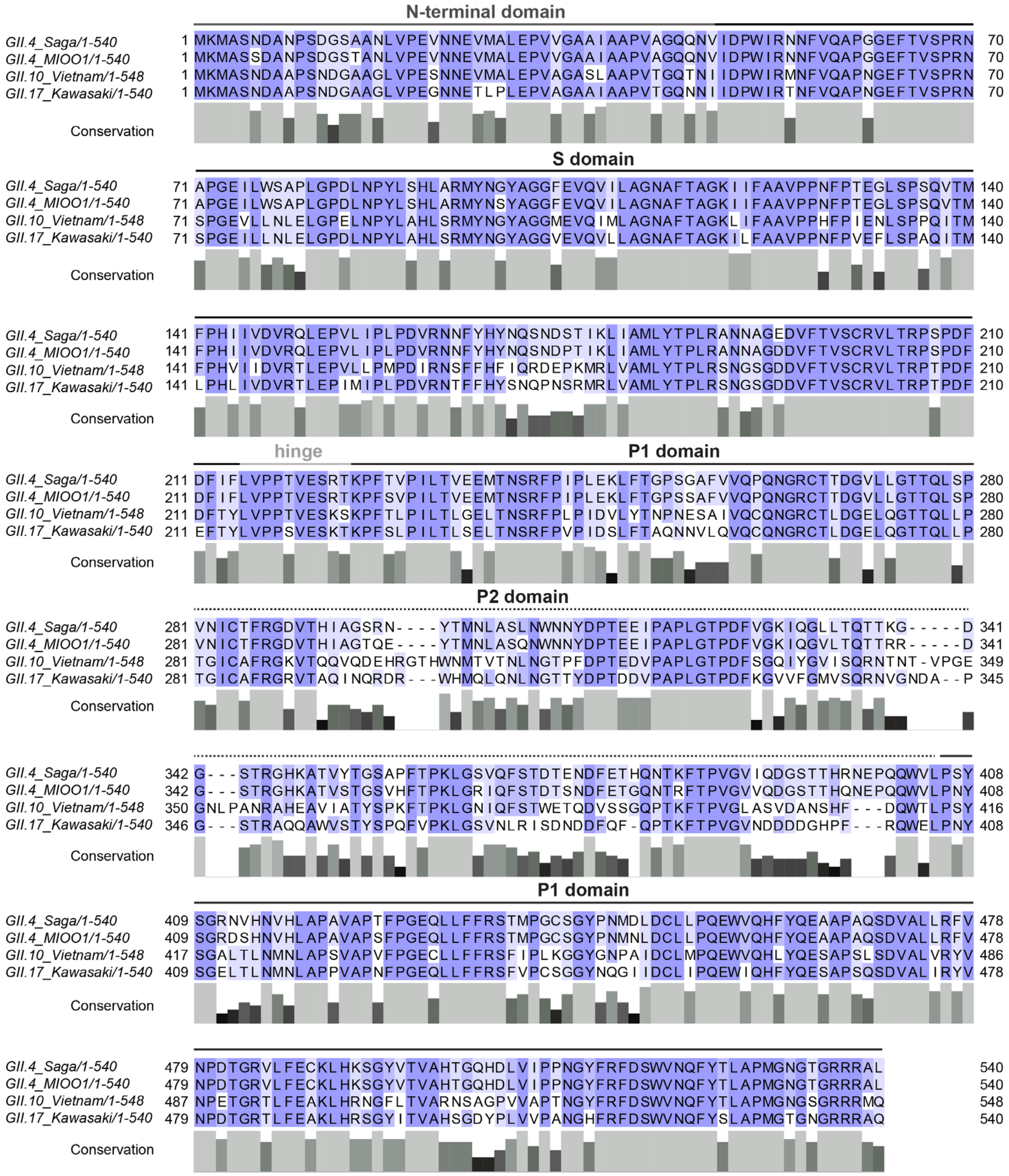

Figure S 1: Sequence alignment of GII.4 Saga, GII.4 MI001, GII.10 Vietnam and GII.17 Kawasaki major capsid protein (VP1) sequences. Color coding represents sequence identity (blue), similarity (light blue) or difference (white). Bars below the sequence indicate the degree of amino acid conservation among the strains at the respective position (low (black) to high (light grey)).

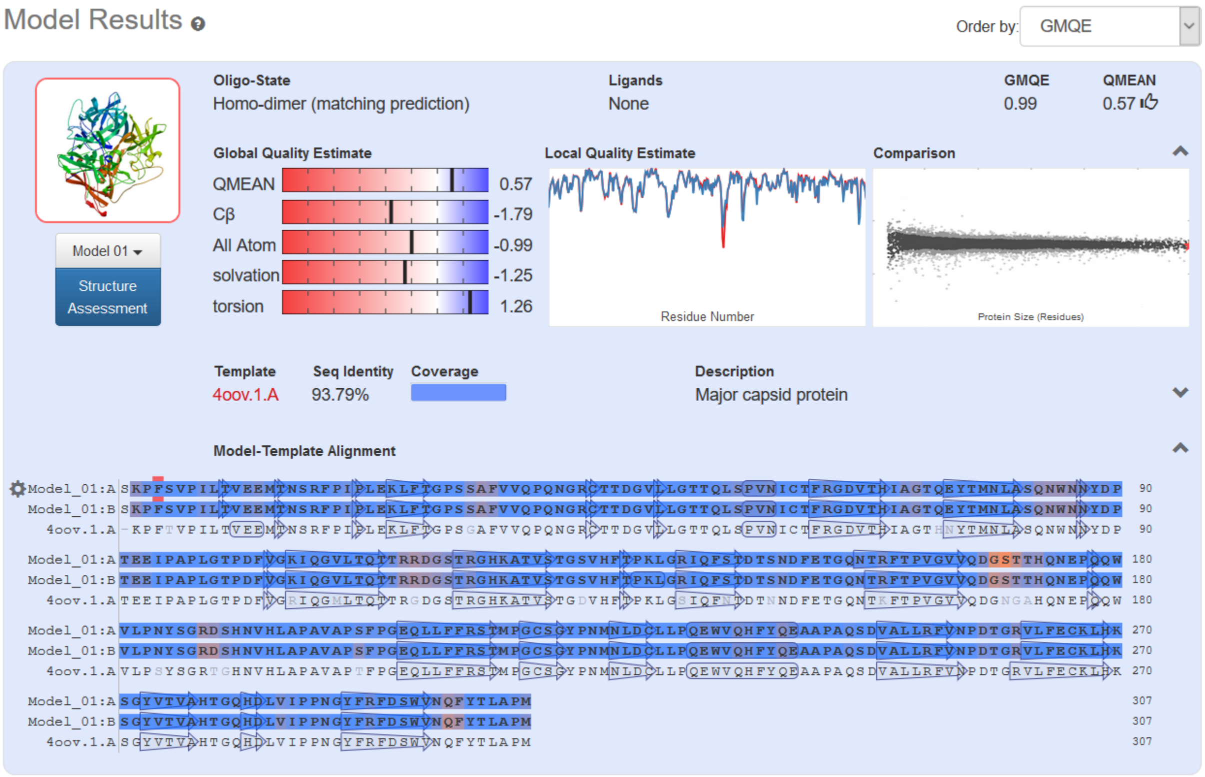

Figure S 2: SWISS MODEL result for the GII.4 MI001 P dimer homology model indicating good model accuracy.

1.2 Identification of deamidation

Table S 1: Identification of deamidation sites in GII.4 MI001 and GII.4 Saga P dimers stored in buffers of different pH. No deamidation sites were identified in GII.10 Vietnam and GII.17 Kawasaki P dimers. Deamidation positions and probability were calculated in MaxQuant based on peptide fragment spectra.

Strain (storage pH)	Peptide sequence	Deamidated position	Deamidation position probability	deamidated fraction in %
**GII.4 MI001 (pH 4.9)**	RSTMPGCSGYPNM**N**L	N448	0.99	8
**GII.4 MI001 pH (7.3)**	FRSTMPGCSGYPNM**N**L	N448	0.82	2
	STDTS**N**DFETGQNTRF	N373	1	approx. 64
	**N**SRFPIPLEKL	N239	0.98	7
**GII.4 Saga pH (7.3)**	STDTE**N**DFETHQ	N373	0.96	approx. 88

A

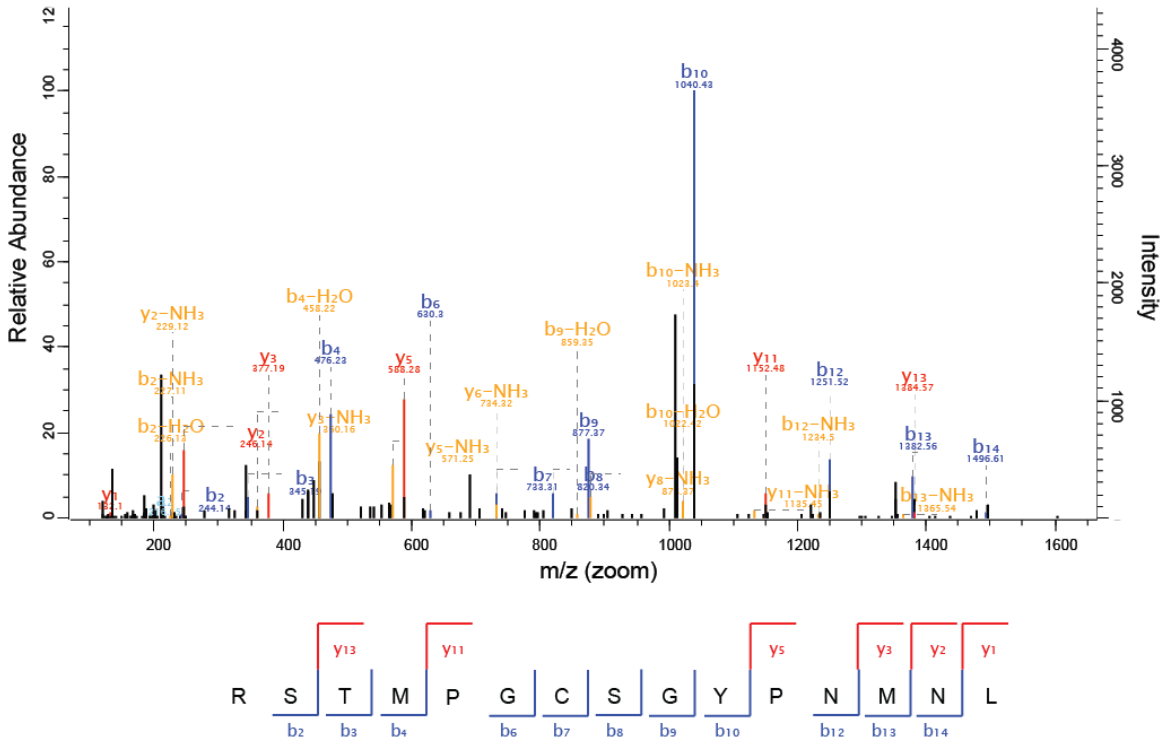

B

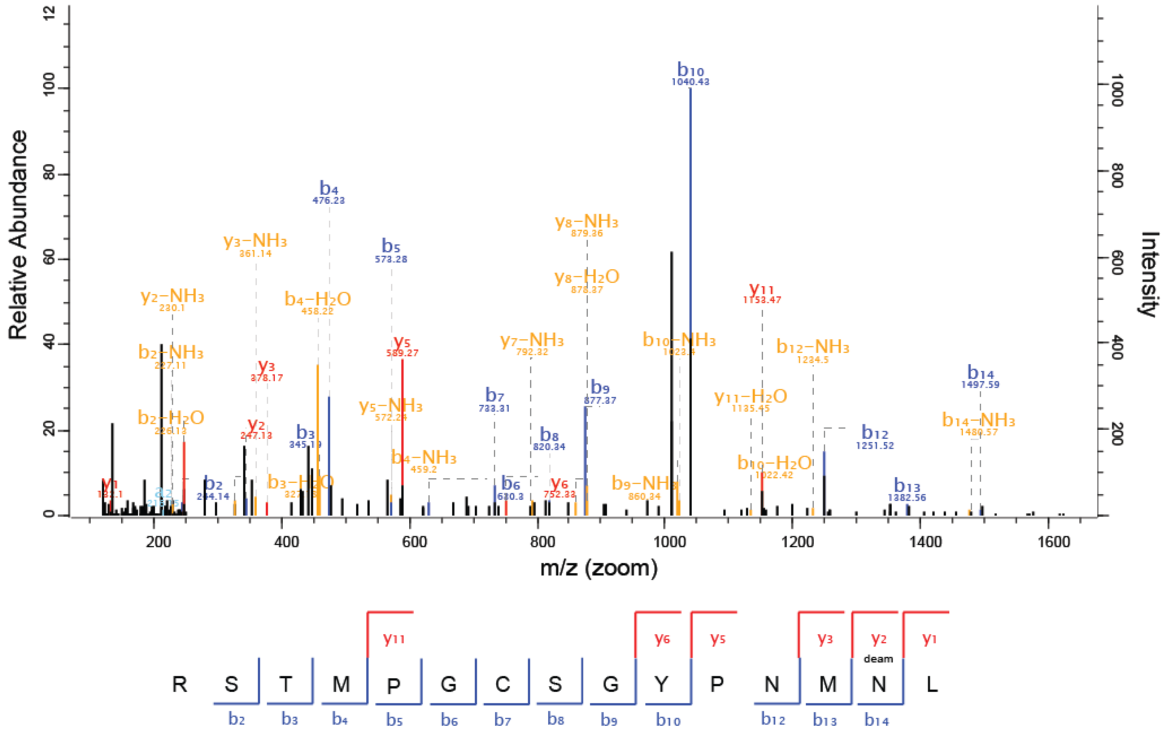

Figure S 3: Fragment spectra of wildtype (A) and deamidated (B) peptide RSTMPGCSGYPNMN[448]L from GII.4 MI001 P dimer stored at pH 4.9 (5 months, 5°C).

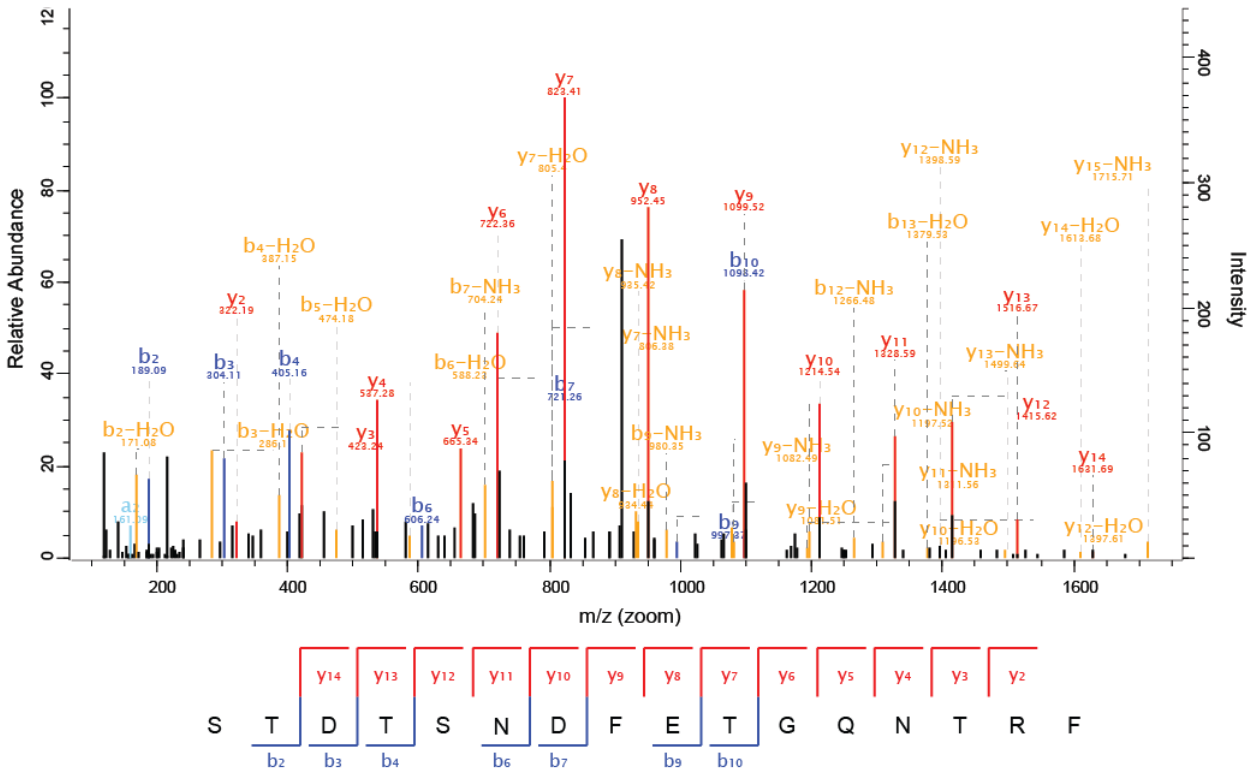

Figure S 4: Fragment spectra of wildtype peptide STDTSN[373]DFETGQNTRF from GII.4 MI001 P dimer stored at pH 4.9 (5 months, 5°C). No peptide carrying a deamidation at N373 could be detected.

A

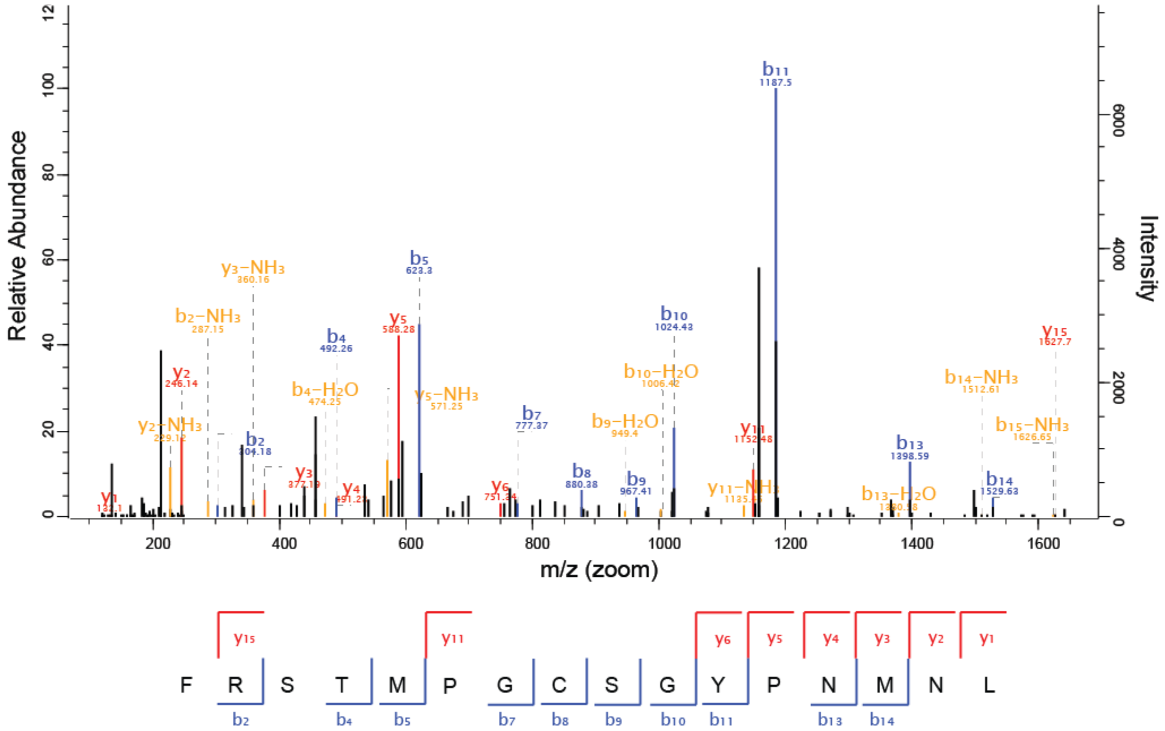

B

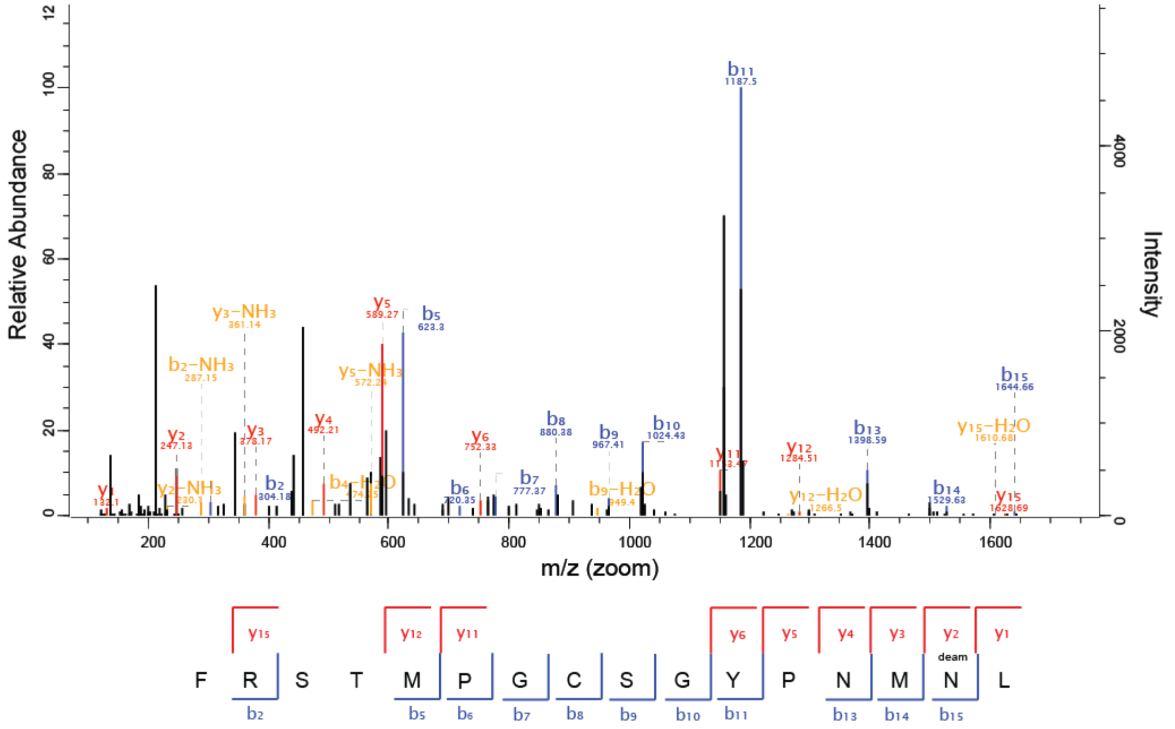

Figure S 5: Fragment spectra of wildtype (A) and deamidated (B) peptide FRSTMPGCSGYPNMN[448]L from GII.4 MI001 P dimer stored at pH 7.3 (1 year, 5°C).

A

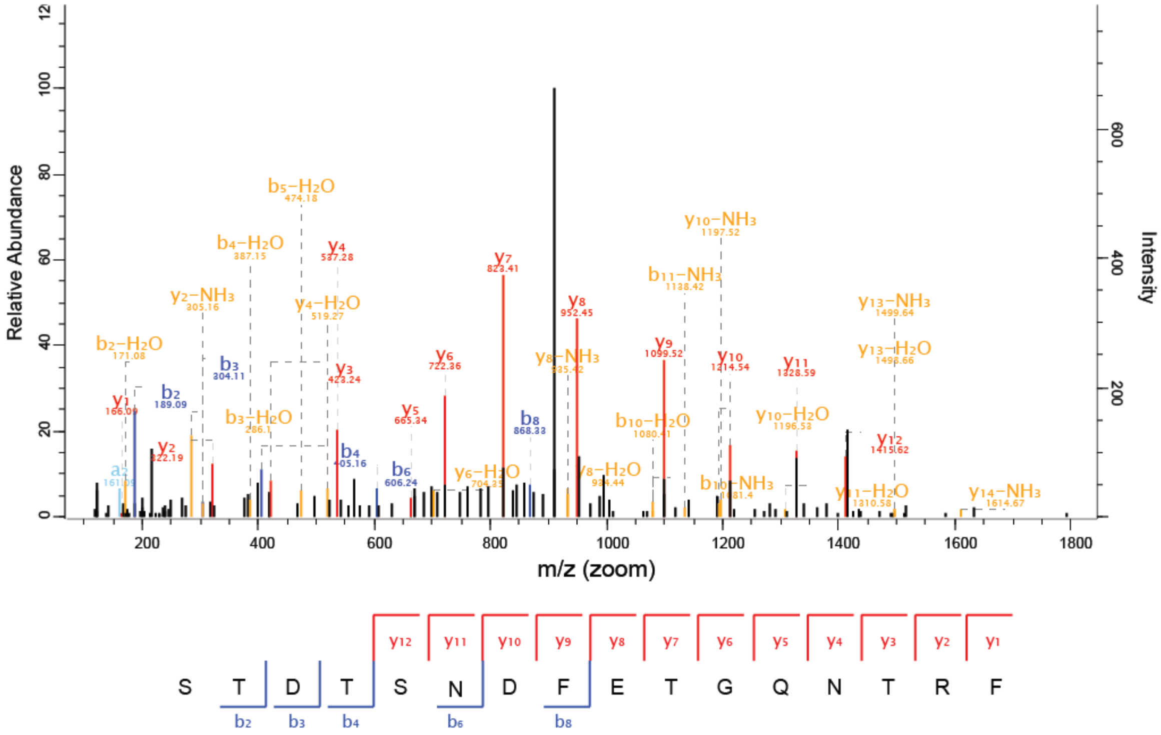

B

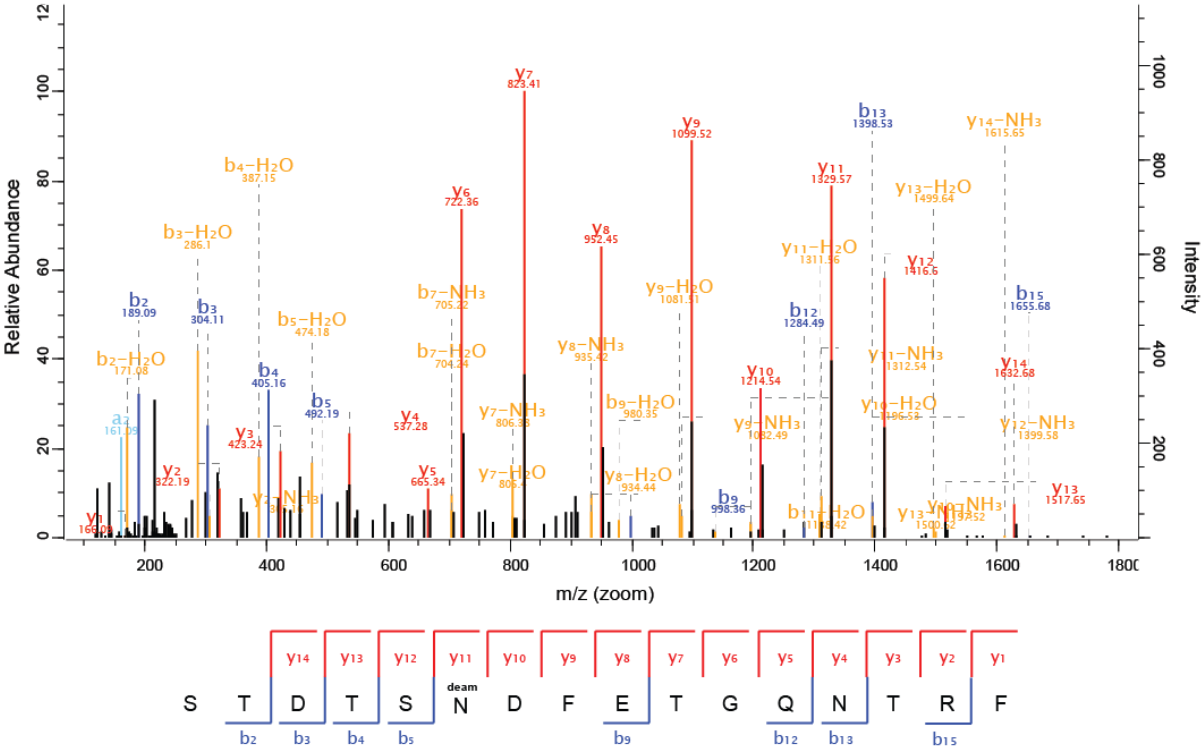

Figure S 6: Fragment spectra of wildtype (A) and deamidated (B) peptide STDTSN373DFETGQNTRF from GII.4 MI001 P dimer stored at pH 7.3 (1 year, 5°C).

A

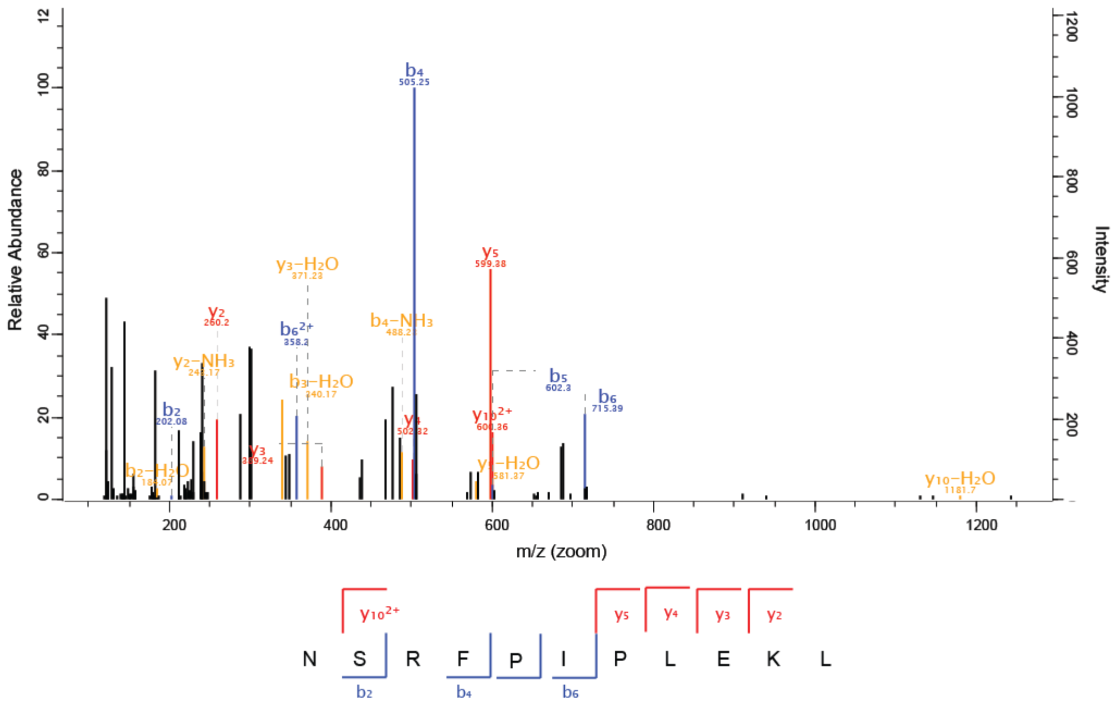

B

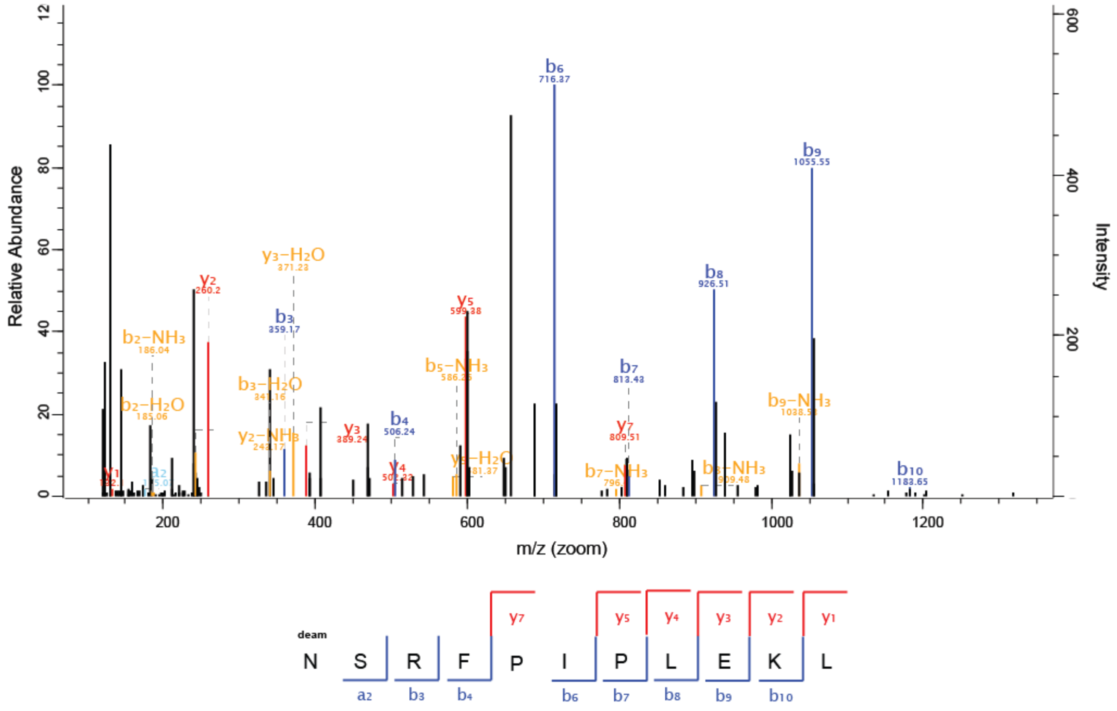

Figure S 7: Fragment spectra of wildtype (A) and deamidated (B) peptide N[239]SRFPIPLEKL from GII.4 MI001 P dimer stored at pH 7.3 (1 year, 5°C).

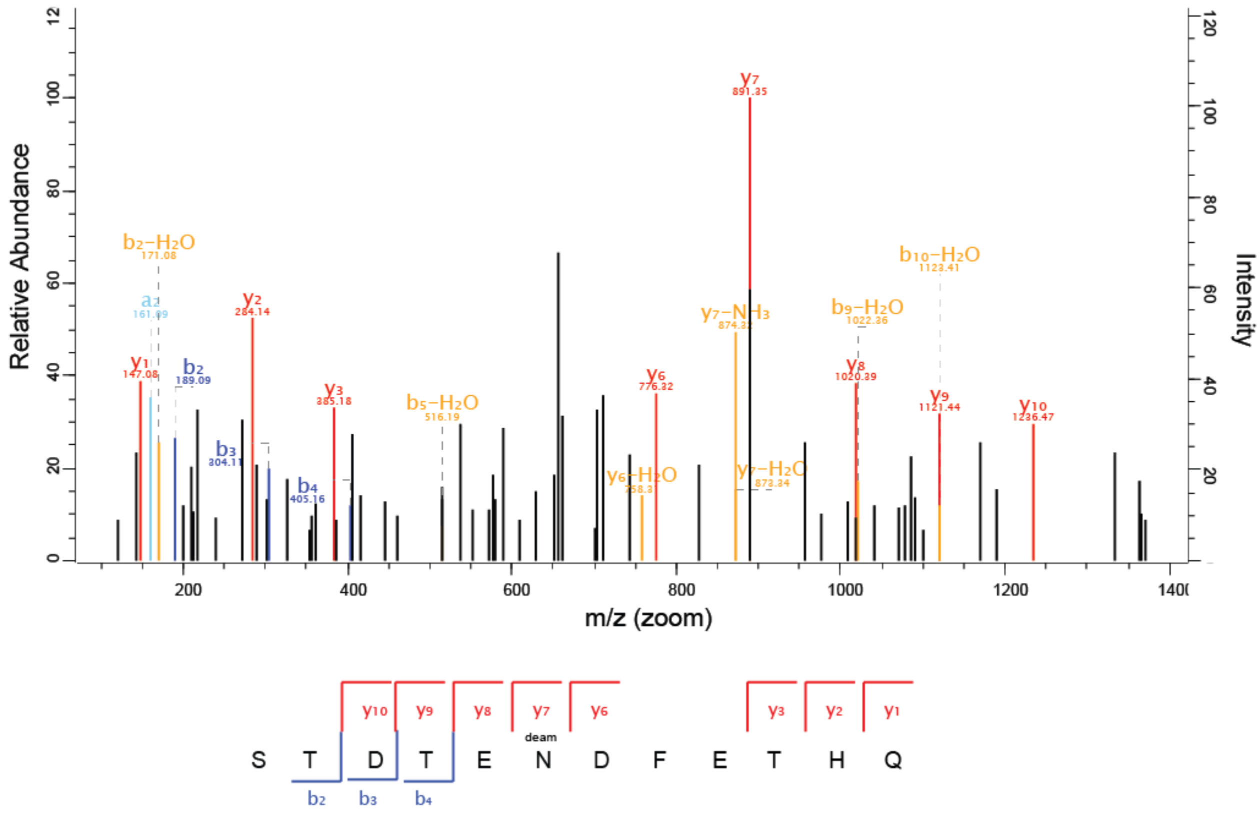

Figure S 8: Fragment spectra of deamidated peptide STDTEN[373]DFETHQ from GII.4 Saga P dimer stored at pH 7.3 (more than 2 years, 5°C). Due to the low abundance wildtype N373 peptides did not get selected for fragmentation in the mass spectrometer and could therefore not be identified in the automated peptide search in MaxQuant. Therefore, MS data was searched manually for wildtype peptide precursors, which led to the identification of approximately 12 % N373 wildtype peptides.

A

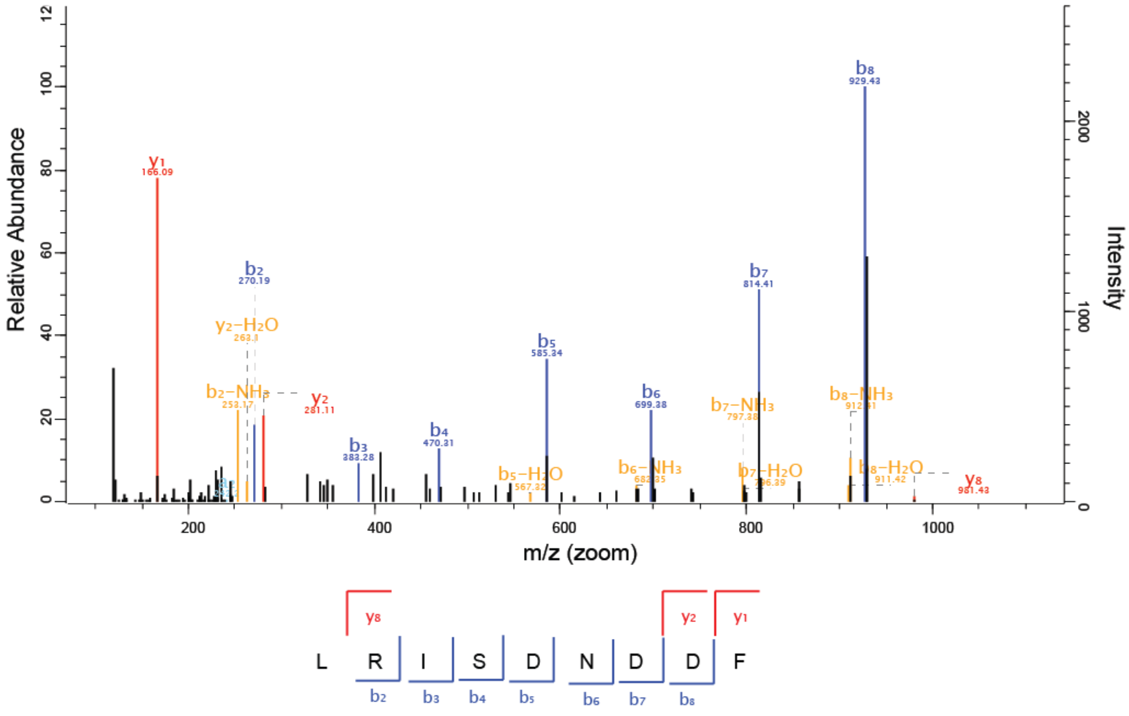

B

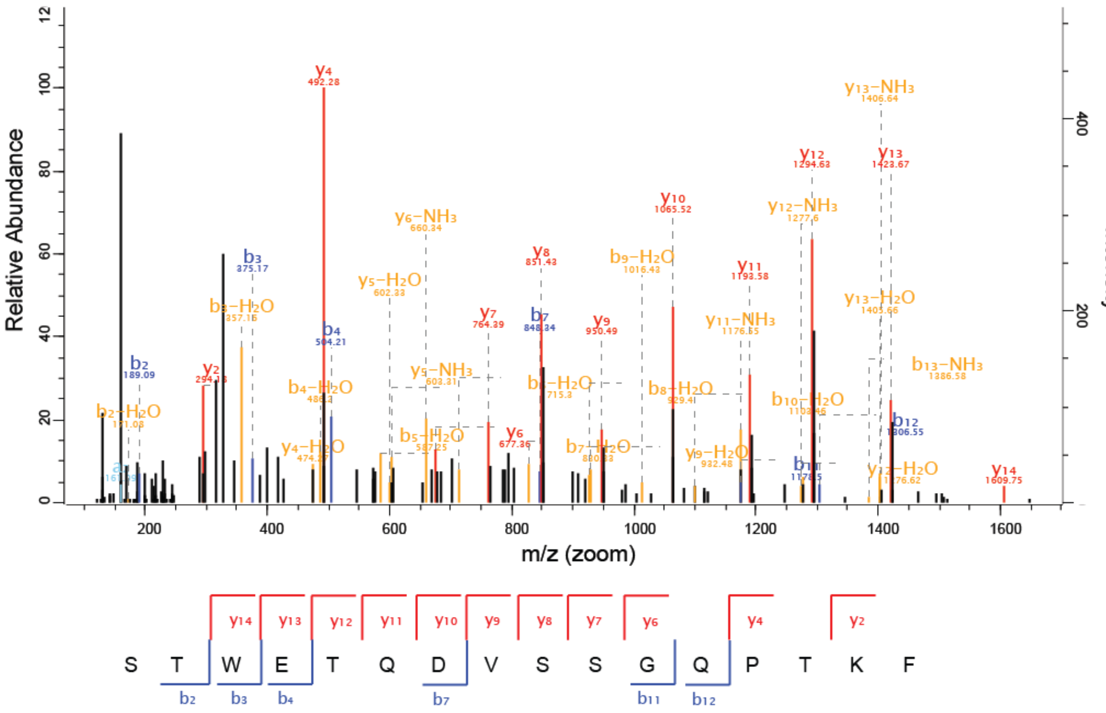

Figure S 9: Fragment spectra of peptides LRISDND[377]DF from GII.17 Kawasaki P dimer (A) and STWETQ[384]DVSSGQPTKF from GII.10 Vietnam P dimer (B). Both strains retain their wildtype sequence at the GII.4 N373 equivalent positon, even after more than a year of storage at 5°C.

1.3 Native MS

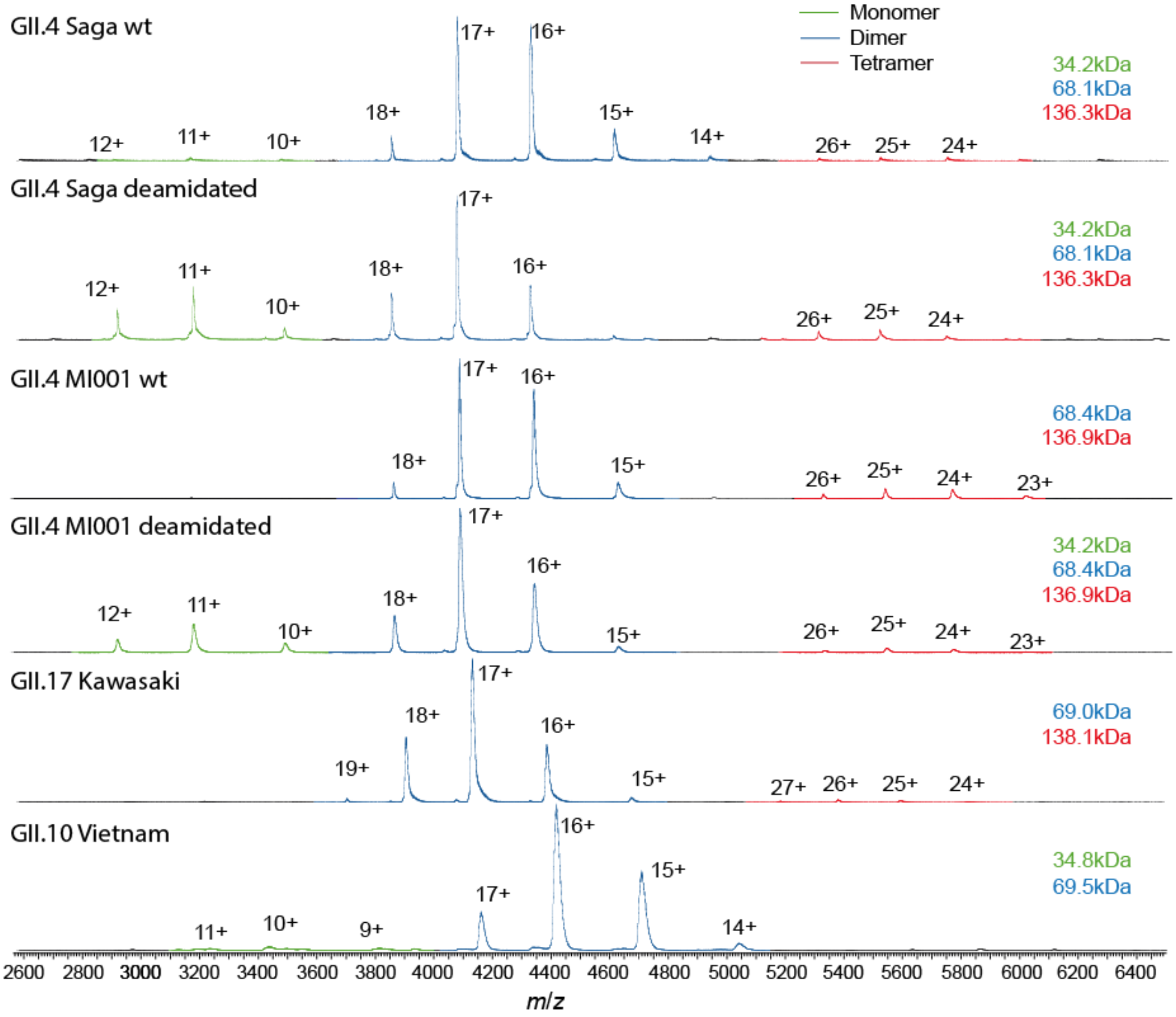

Figure S 10: Native mass spectra of different human norovirus P domain samples. GII.17 Kawasaki, GII.10 Vietnam, wildtype GII.4 MI001 and wildtype GII.4 Saga P domains are present as dimers with the expected molecular masses, apart from unspecific tetramers formed during the ESI process. Both deamidated GII.4 P domains (100 % deamidated GII.4 Saga and 64 % deamidated GII.4 MI001) are also present as monomers. No native MS data is available for the partially (88 %) deamidated GII.4 Saga P dimer. Quantification of the individual species in each strain can be found in Table S1.

Table S 2: Fractions of P monomers, dimers and tetramers from native MS (Figure S10)

**Strain**	**Monomer fraction (%)**	**Dimer fraction (%)**	**Tetramer fraction (%)**
**GII.4 Saga deamidated (100%)**	**32**	**60**	8
**GII.4 Saga wildtype**	0	**100**	0
**GII.4 MI001 deamidated (64%)**	**16**	**80**	4
**GII.4 MI001 wildtype**	0	**91**	9
**GII.17 Kawasaki**	0	**100**	0
**GII.10 Vietnam**	4	**96**	0

1.4 Analysis of bimodal spectra

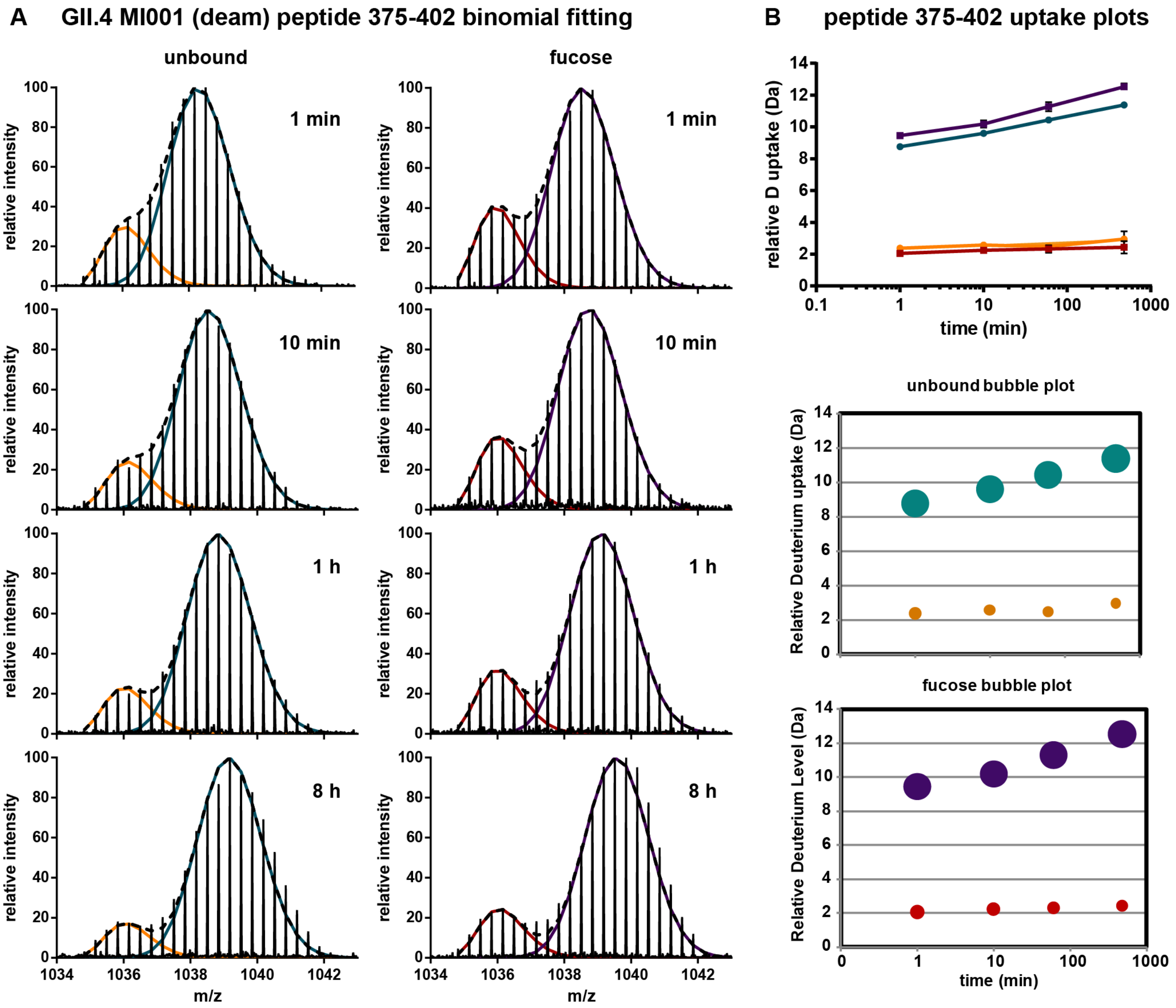

Figure S 11: Peptide HDX data analysis example for binomial fitting of bimodal spectra. A) Binomial fitting with bimodal deconvolution was applied in HXExpress resulting in a low (orange, red) and a high (green, violet) deuterated peak distribution for every state and time point. B) Deuteration values for the individual peak distributions can be visualized as uptake plot and significant differences between the unbound and ligand-bound state can be statistically analyzed. The relative deuterium uptake for each peak distribution can also be displayed as bubble plot, where the circle area corresponds to the relative intensity of the individual peak distribution at a given time point. Here, constant intensity ratios over time point towards the presence of two conformationally distinct protein subpopulations.

Table S 3: Representative uptake plots for peptides with significant deuteration differences according to Table 1.

**Region with significant changes in HDX**	**Bimodal peak distribution**	**Representative Uptake Plot**<br><br>(centroid analysis for unimodal peak distributions, individual binomial fitting of both peak distributions (1st, 2nd) for bimodal peak distributions)
**GII.10 Vietnam P dimer + 100 mM fucose**		
311-336	No	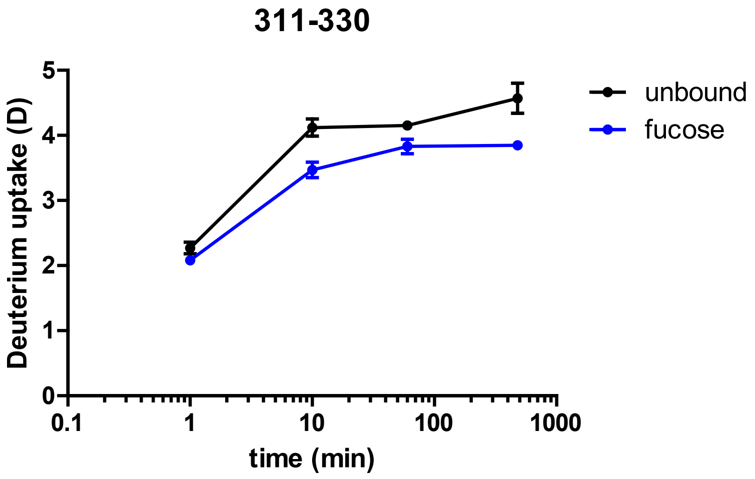
337-364	Yes	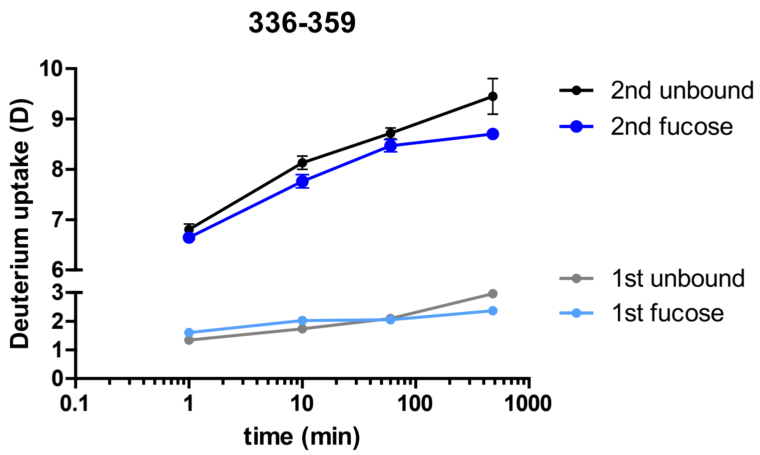

379-399	Yes	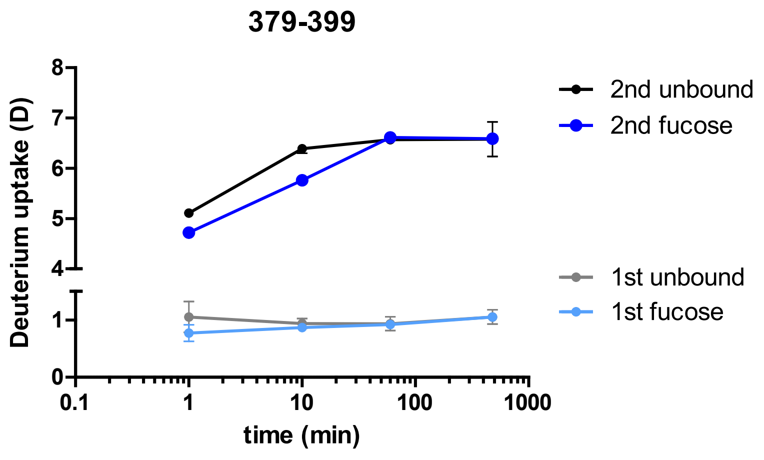
442-458	No	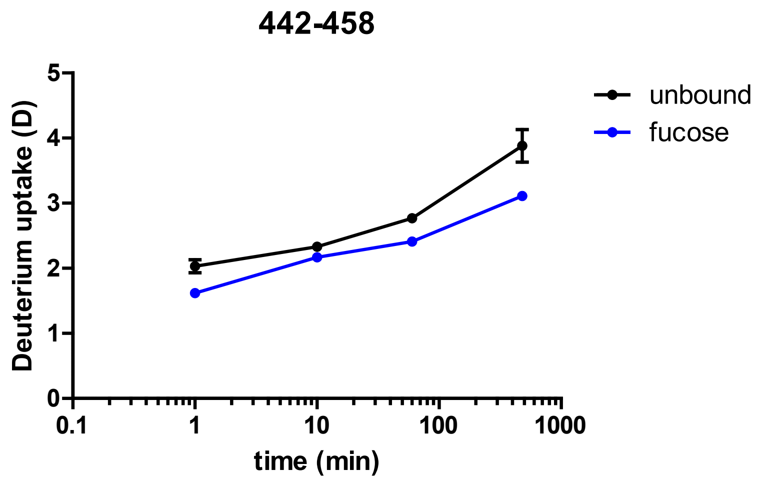
**GII.10 Vietnam P dimer + 10 mM B trisaccharide**		
336-361	Yes	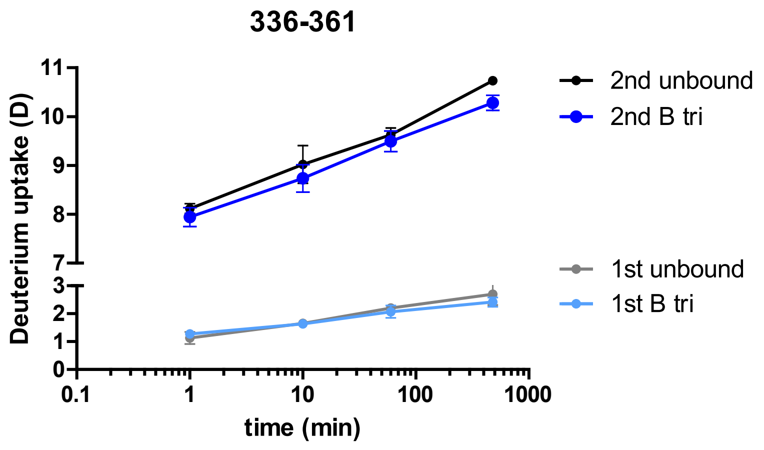

<table>
<tr><td>379-428</td><td>Yes</td><td>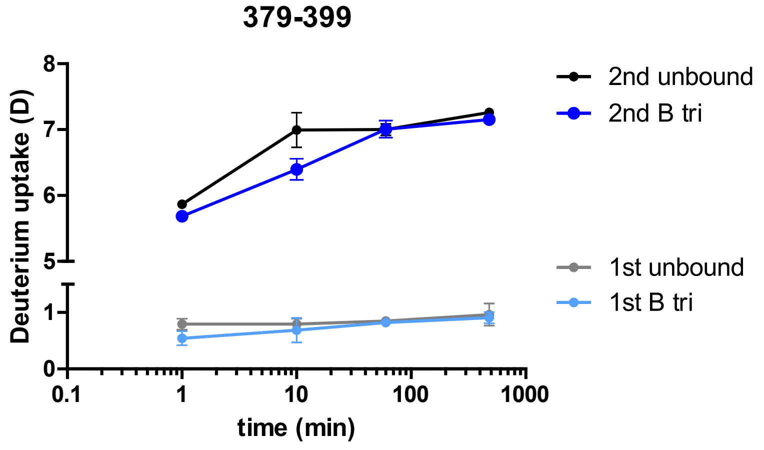

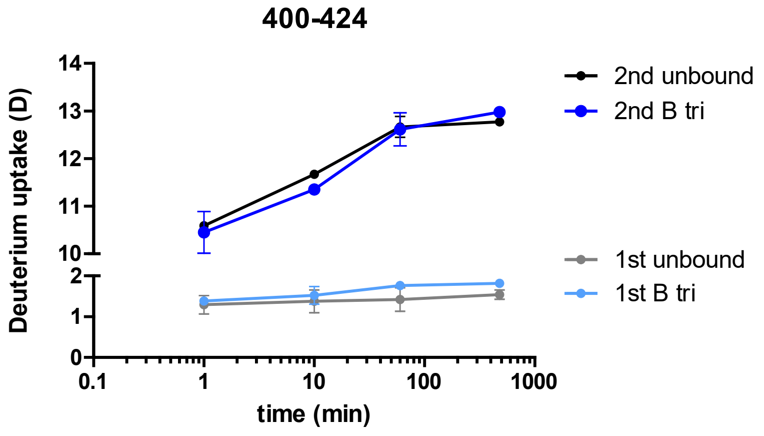
</td></tr>
<tr><td>440-458</td><td>No</td><td>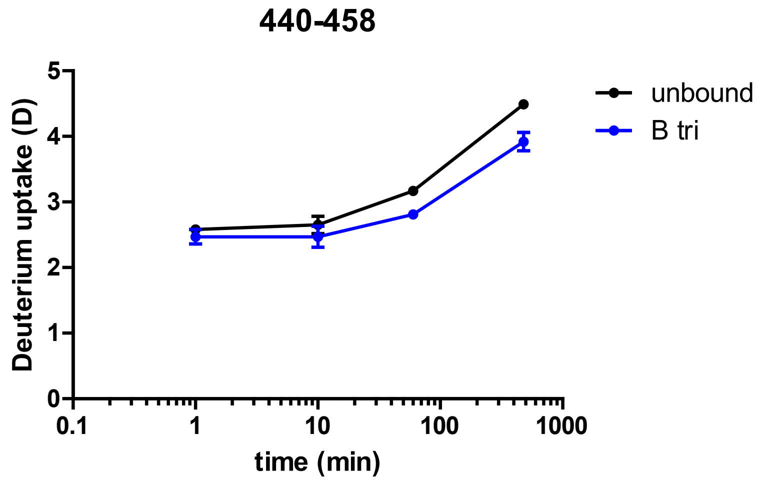
</td></tr>
</table>

<table>
<tr><td>483-496</td><td>Yes</td><td>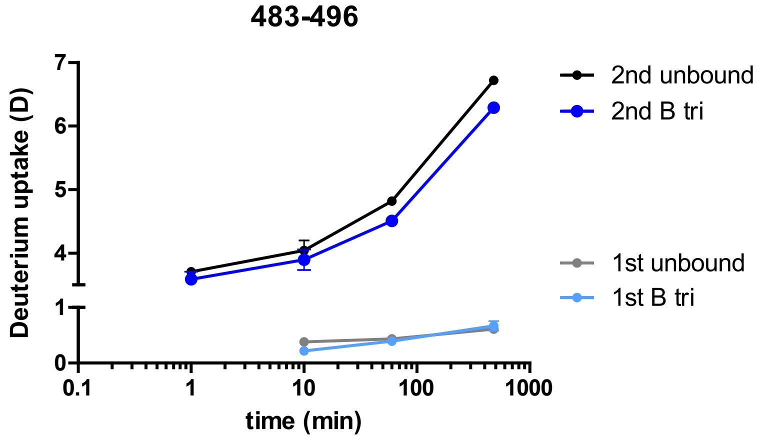
</td></tr>
<tr><td colspan="3">GII.17 Kawasaki P dimer + 100 mM fucose</td></tr>
<tr><td>269-286</td><td>No</td><td>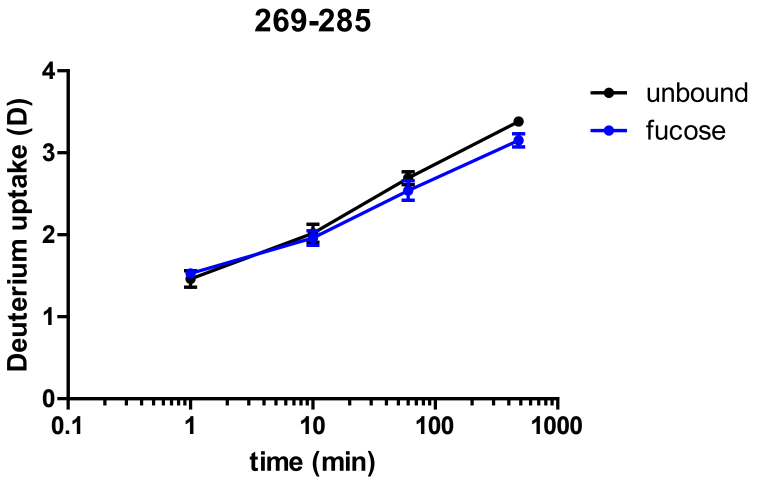
</td></tr>
<tr><td>333-353</td><td>Yes</td><td>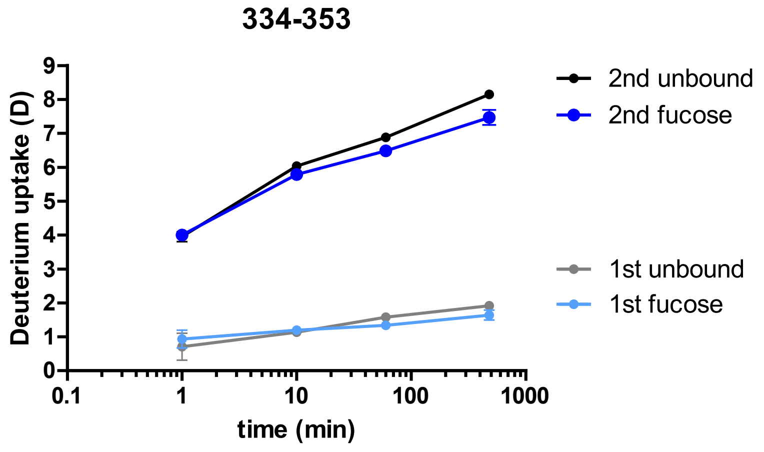
</td></tr>
</table>

434-452	No	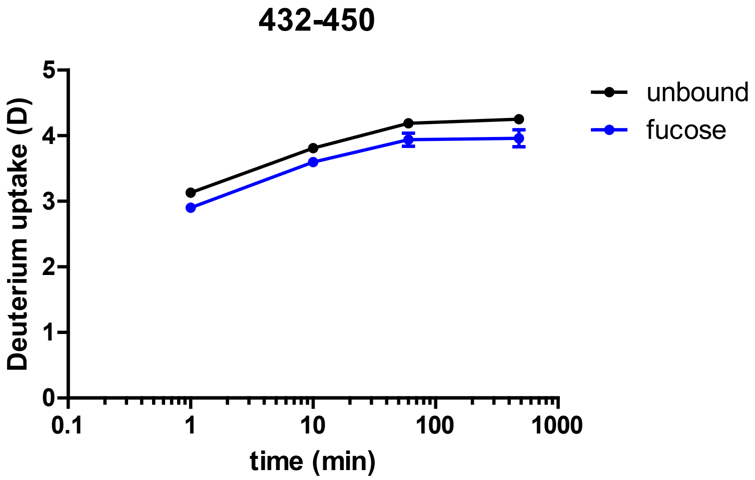
**GII.17 Kawasaki P dimer + 10 mM B trisaccharide**		
333-353	Yes	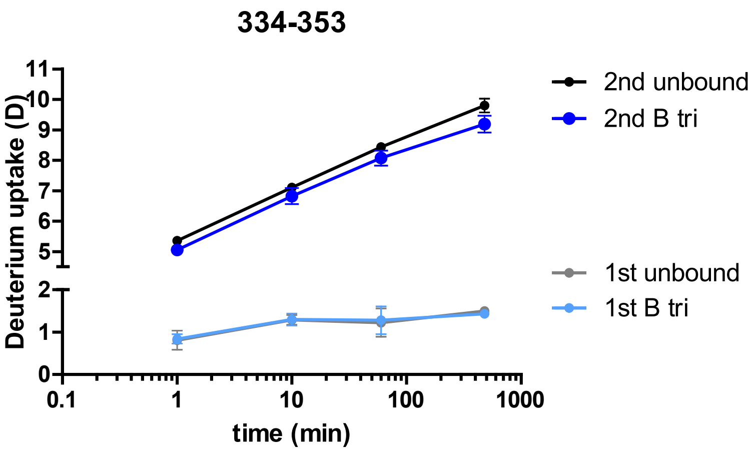
**GII.4 MI001 P dimer wt + 100 mM fucose**		
283-303	Yes	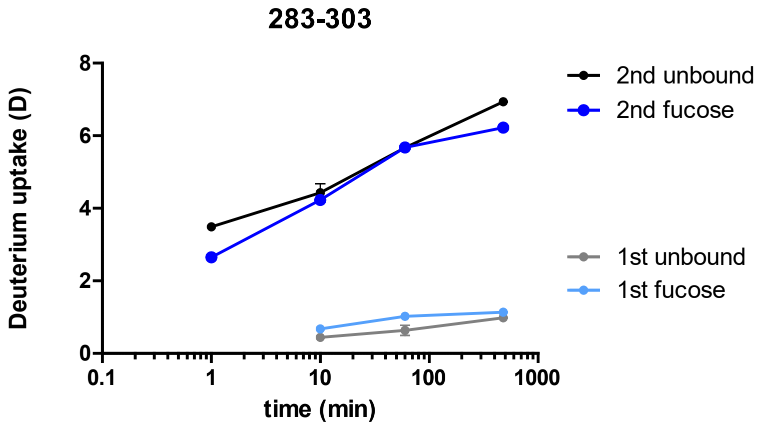

<table>
<tr><td>434-449</td><td>No</td><td>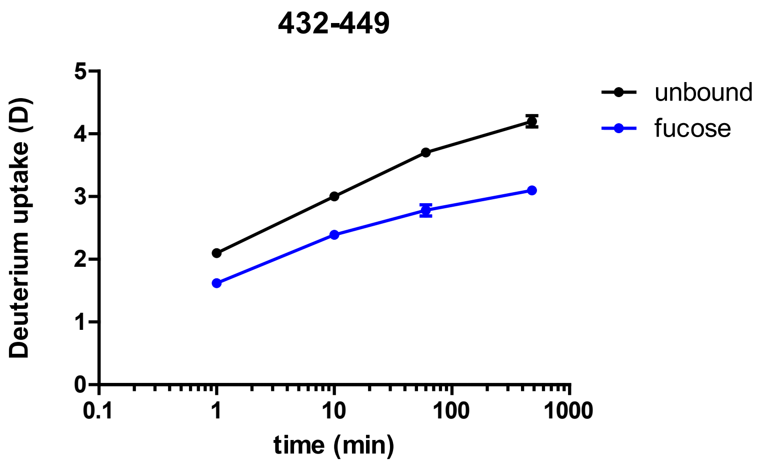
</td></tr>
<tr><td colspan="3">GII.4 MI001 P dimer+ 10 mM B trisaccharide</td></tr>
<tr><td>283-298</td><td>Yes</td><td>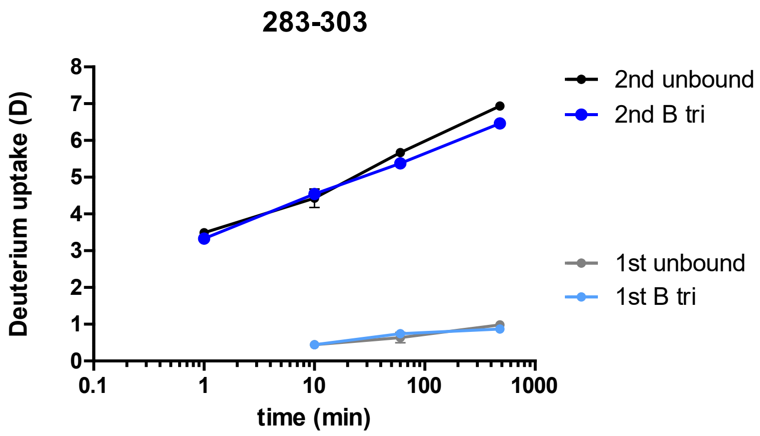
</td></tr>
<tr><td>333-353</td><td>Yes</td><td>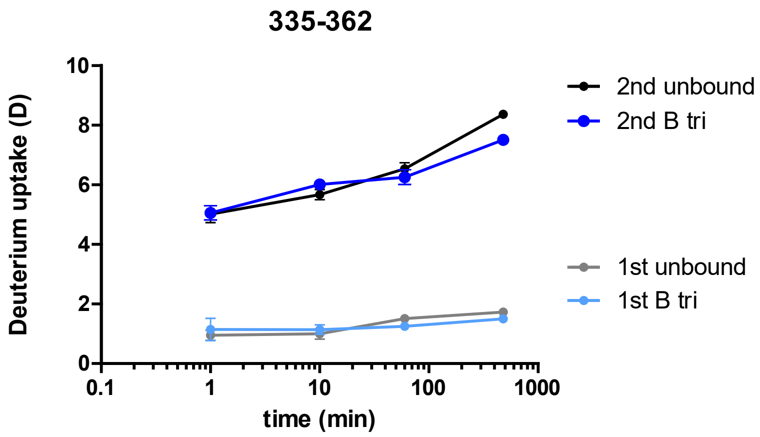
</td></tr>
</table>

434-450	No	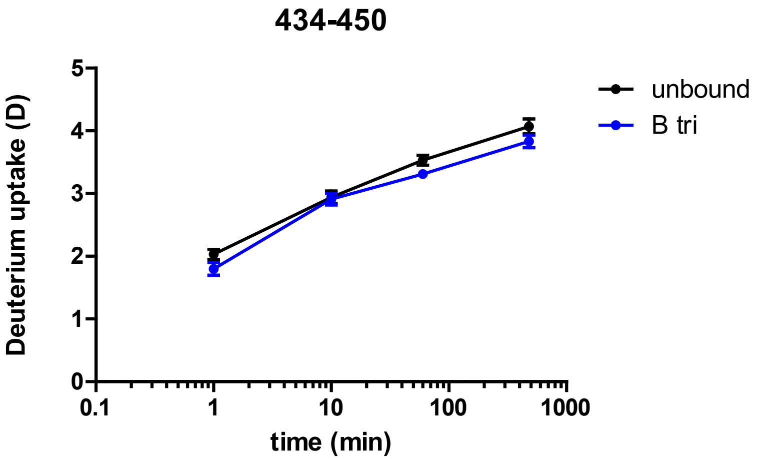
**Partially deamidated GII.4 MI001 P dimer + 100 mM fucose**		
273-286	Broad distribution / not clearly bimodal	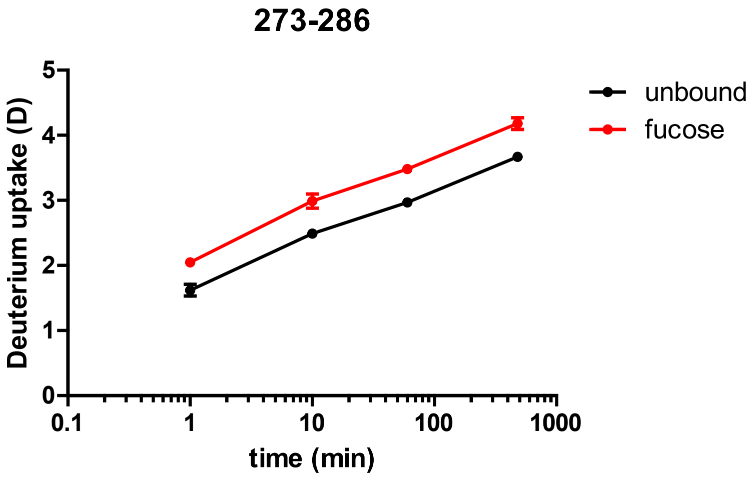
335-372	Yes	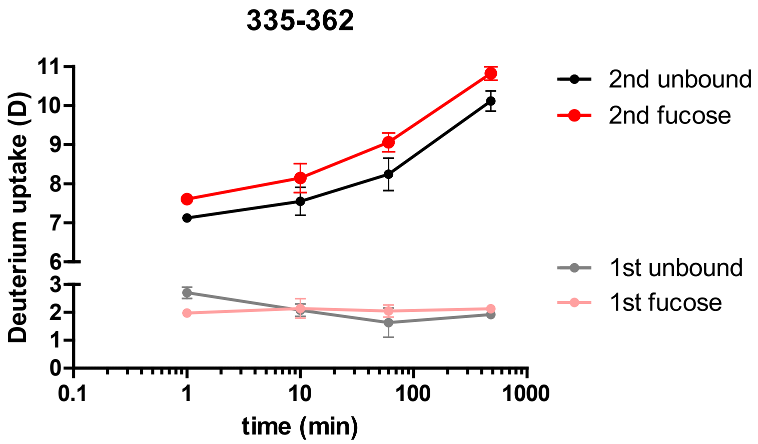

384-402	Yes	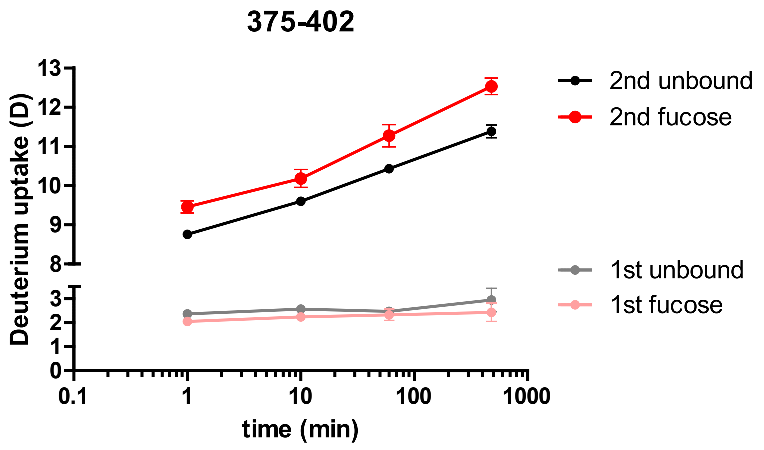
433-449	No	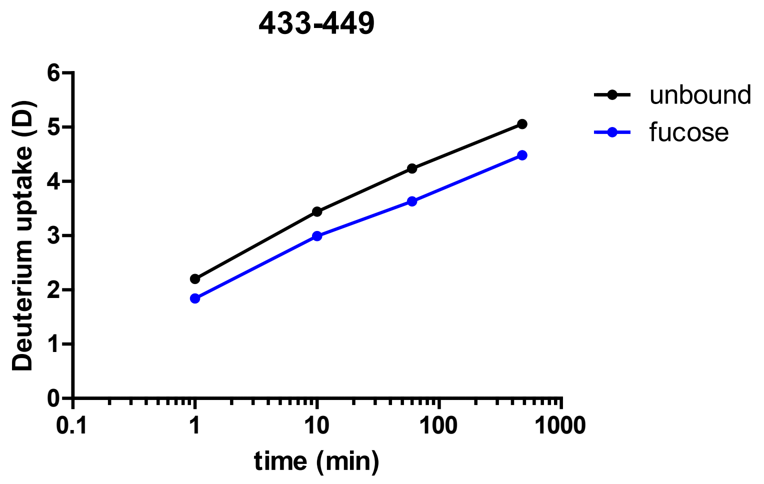
**Partially deamidated GII.4 Saga P dimer + 100 mM fucose**		
287-303	No	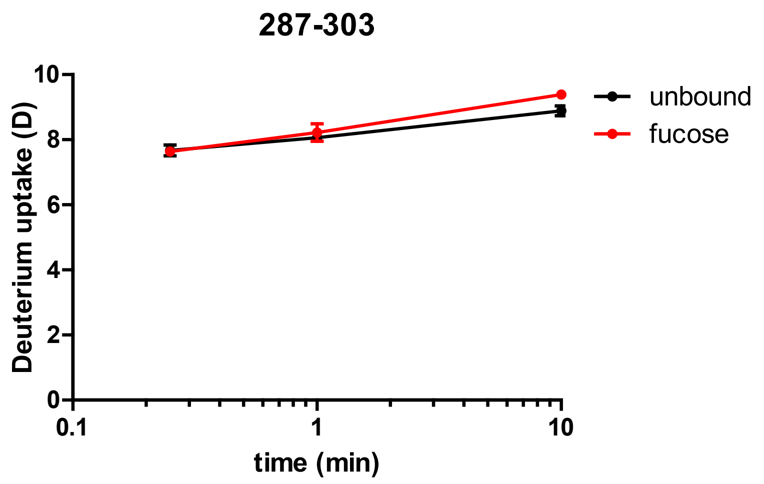

<table>
<tr><td>335-352</td><td>No</td><td>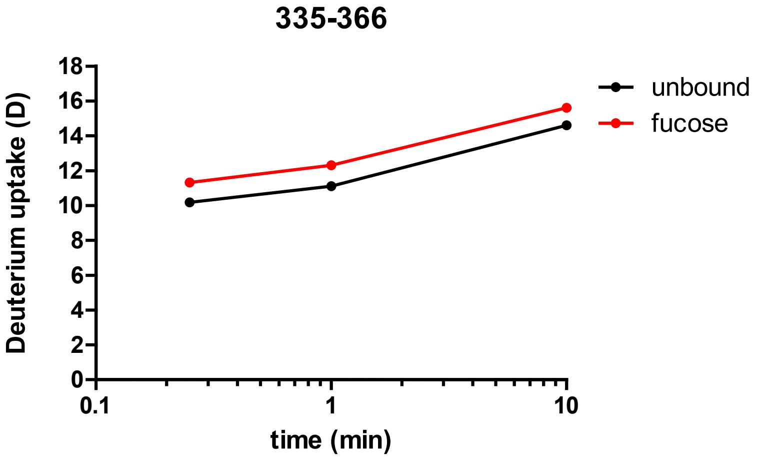
</td></tr>
<tr><td>375-431</td><td>No</td><td>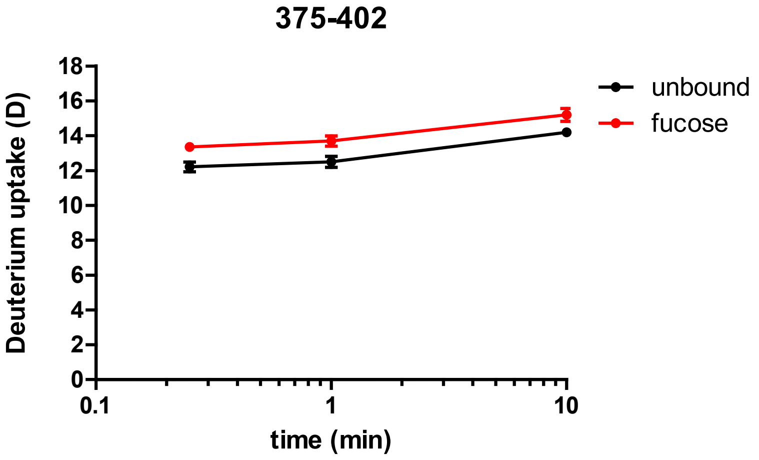

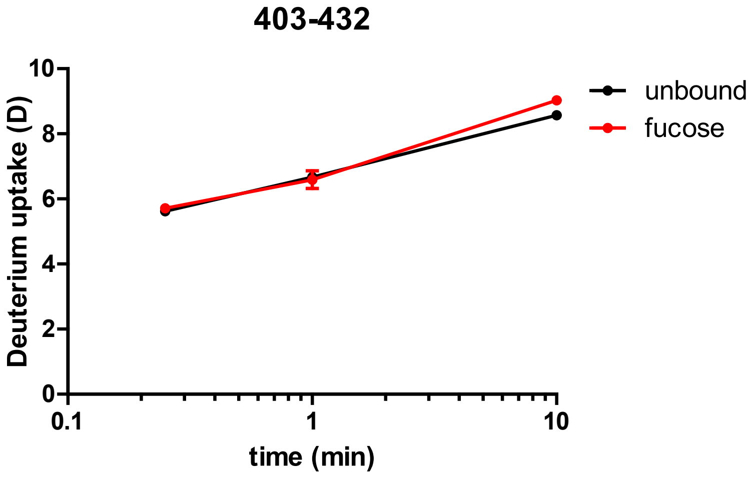
</td></tr>
</table>

432-449	No	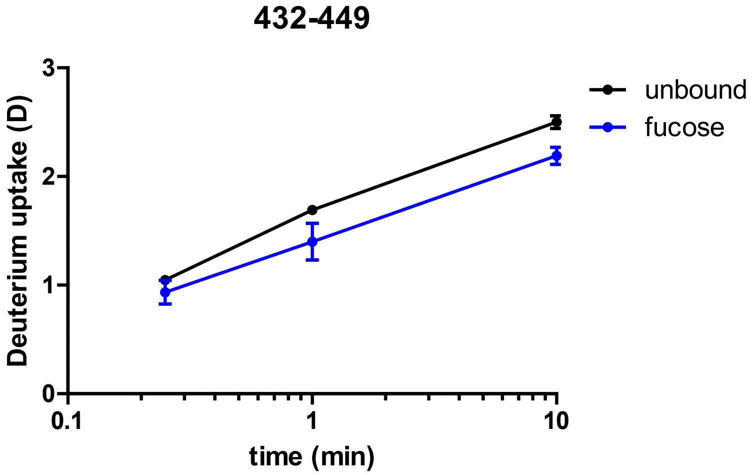 432-449<br>Deuterium uptake (D)<br>3<br>2<br>1<br>0<br>0.1<br>1<br>10<br>time (min)<br>unbound<br>fucose

1.5 Woods Plots

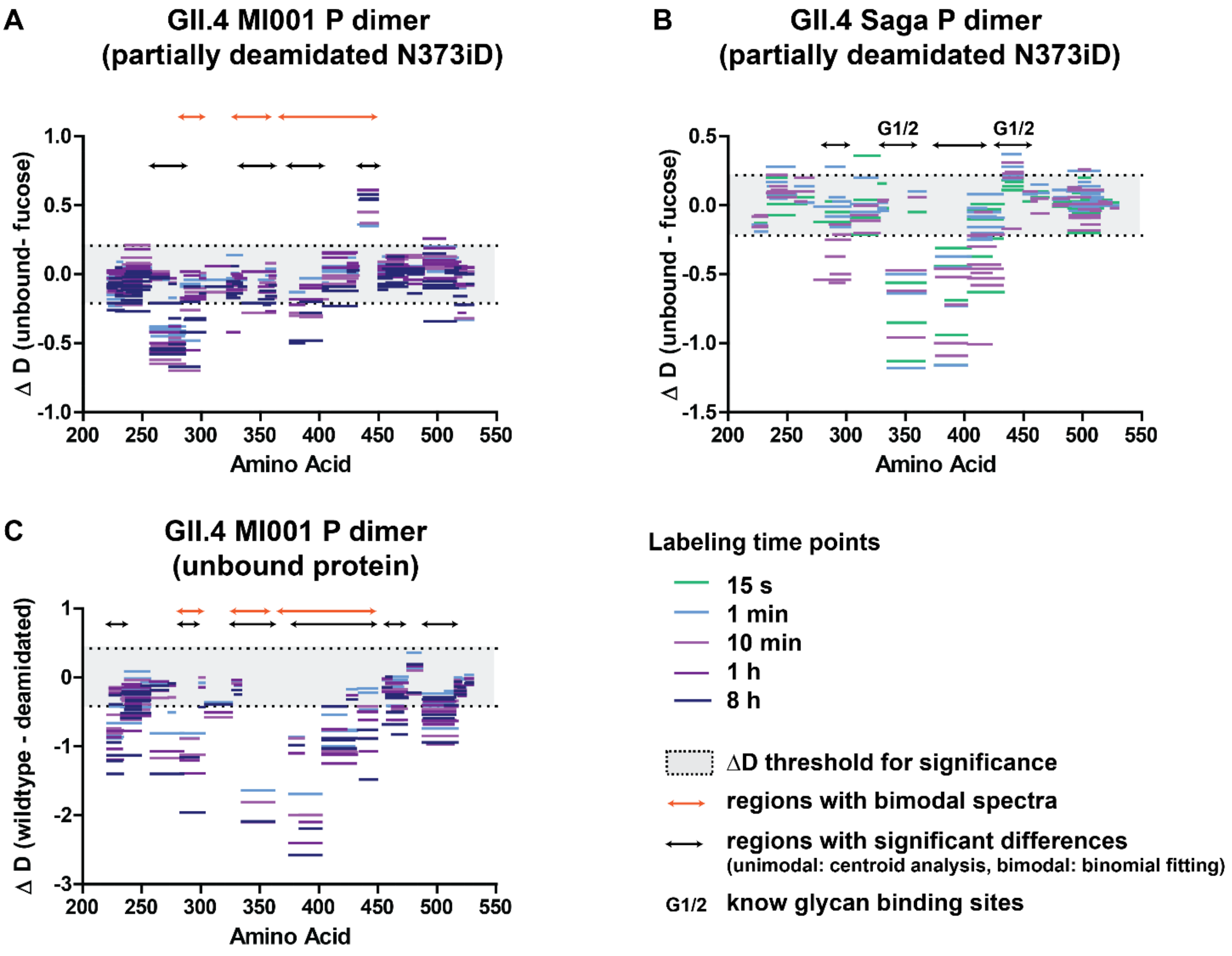

Figure S 12: Woods plots for HDX-MS experiments with partially deamidated P dimers. Depicted are deuteration differences from centroid analysis at different labeling time points in individual comparison experiments (A,B: unbound – ligand; C: wild type – partially deamidated protein without ligand). The ΔD threshold represents 2 x pooled standard deviation of the individual dataset. Arrows indicate regions with bimodal spectra (orange) and regions with significant deuteration differences (black). Significance was assessed via passing the ΔD threshold and Student's t-Test with $p<0.05$ as well as validating deuteration differences for bimodal spectra with binomial fitting (Table S 3). Due to strong bimodality especially in the fucose datasets, not all regions that pass the ΔD threshold are considered statistically significant after manual validation. G1/2 indicates regions with significant deuteration differences that contain known glycan binding sites (Table 1 main manuscript).

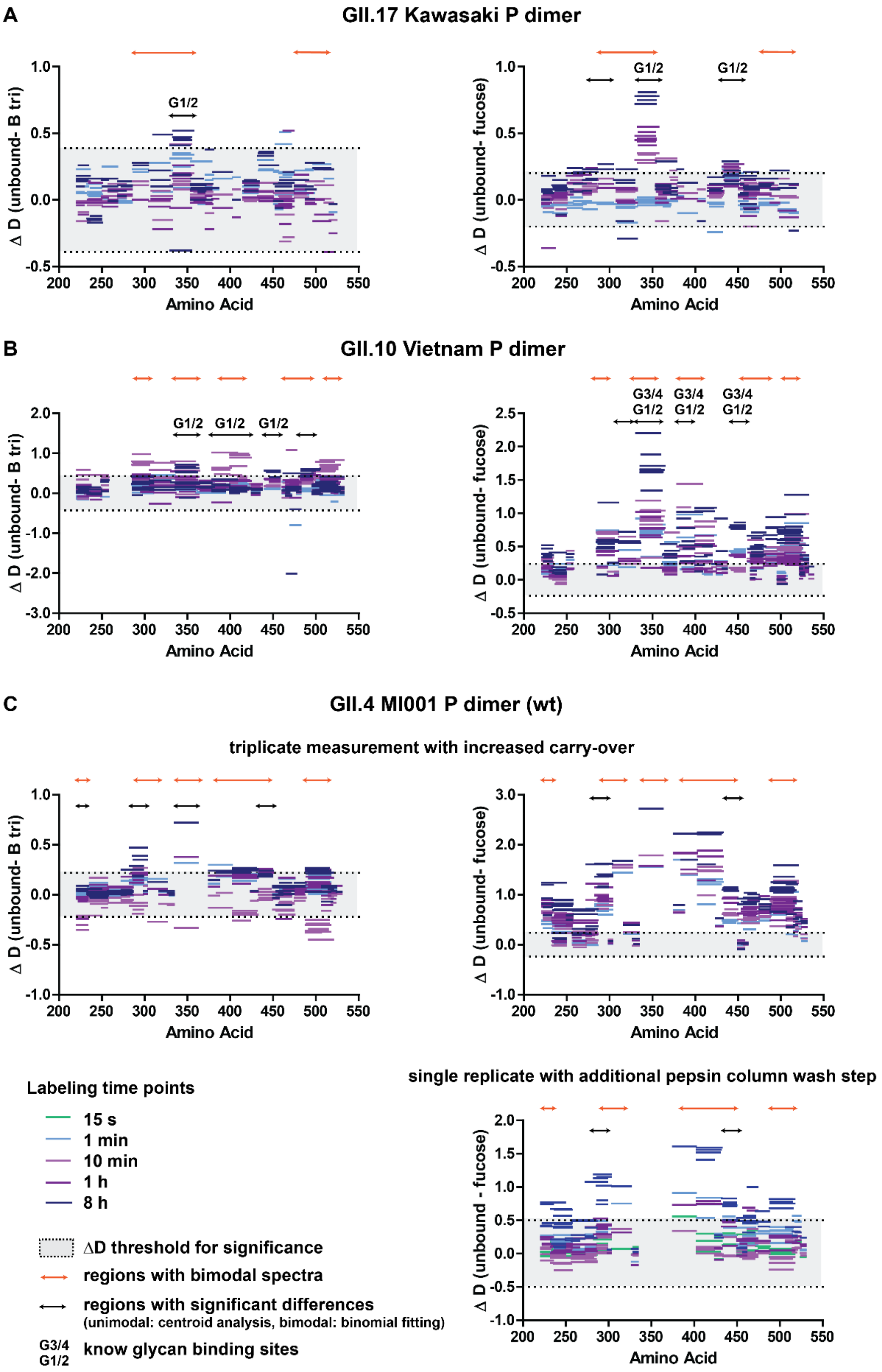

Figure S 13: Woods plots for HDX-MS experiments with partially deamidated P dimers. Depicted are deuteration differences from centroid analysis at different labeling time points in individual comparison experiments (unbound - ligand). The ΔD threshold represents 2 x pooled standard deviation of the individual

dataset. Arrows indicate regions with bimodal spectra (orange) and regions with significant deuteration differences (black). Significance was assessed via passing the ΔD threshold and Student's t-Test with $p<0.05$ as well as validating deuteration differences for bimodal spectra with binomial fitting (Table S 3). Due to strong bimodality especially in the fucose datasets, not all regions that pass the ΔD threshold are considered statistically significant after manual validation. G1/2 indicates regions with significant deuteration differences that contain known glycan binding sites and G3/4 indicates the two additional fucose binding sites only seen in GII.10 Vietnam P dimers (Table 1 main manuscript).

1.6 MD simulations

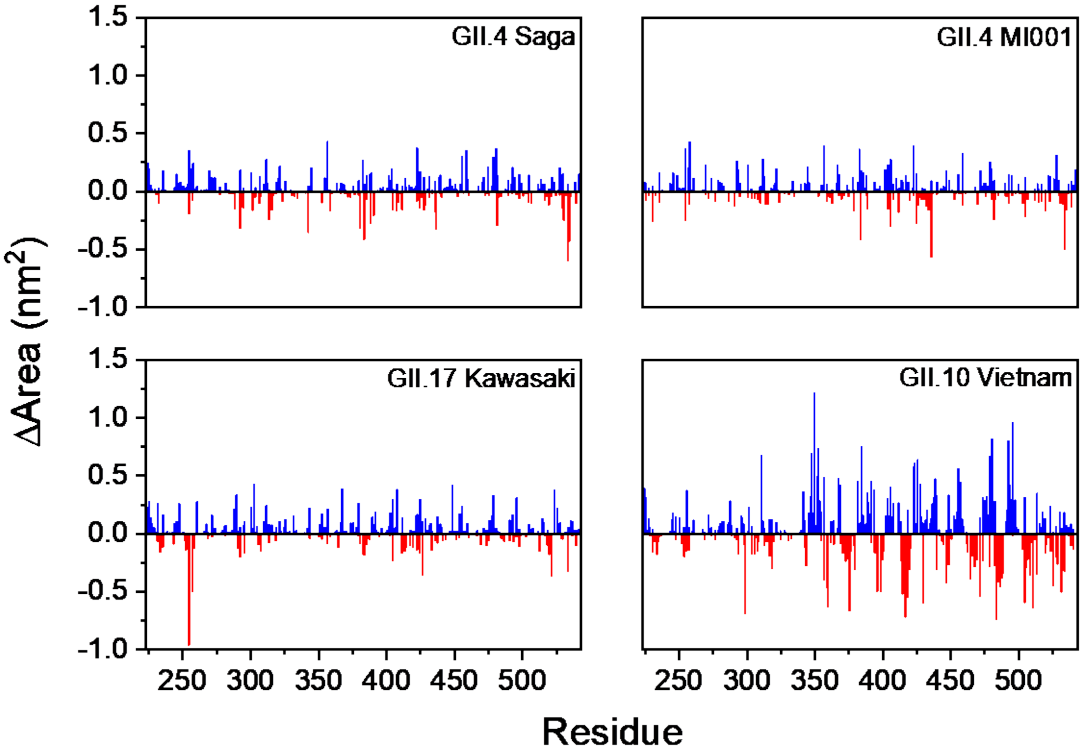

Figure S 14: Solvent accessible surface area calculations for GII.4 Saga, GII.4 MI001, GII.17 Kawasaki and GII.10 Vietnam. Blue peaks describe an increase of surface area per residue, whilst red peaks represent a decrease of the area throughout the simulations as compared to the initial structure.

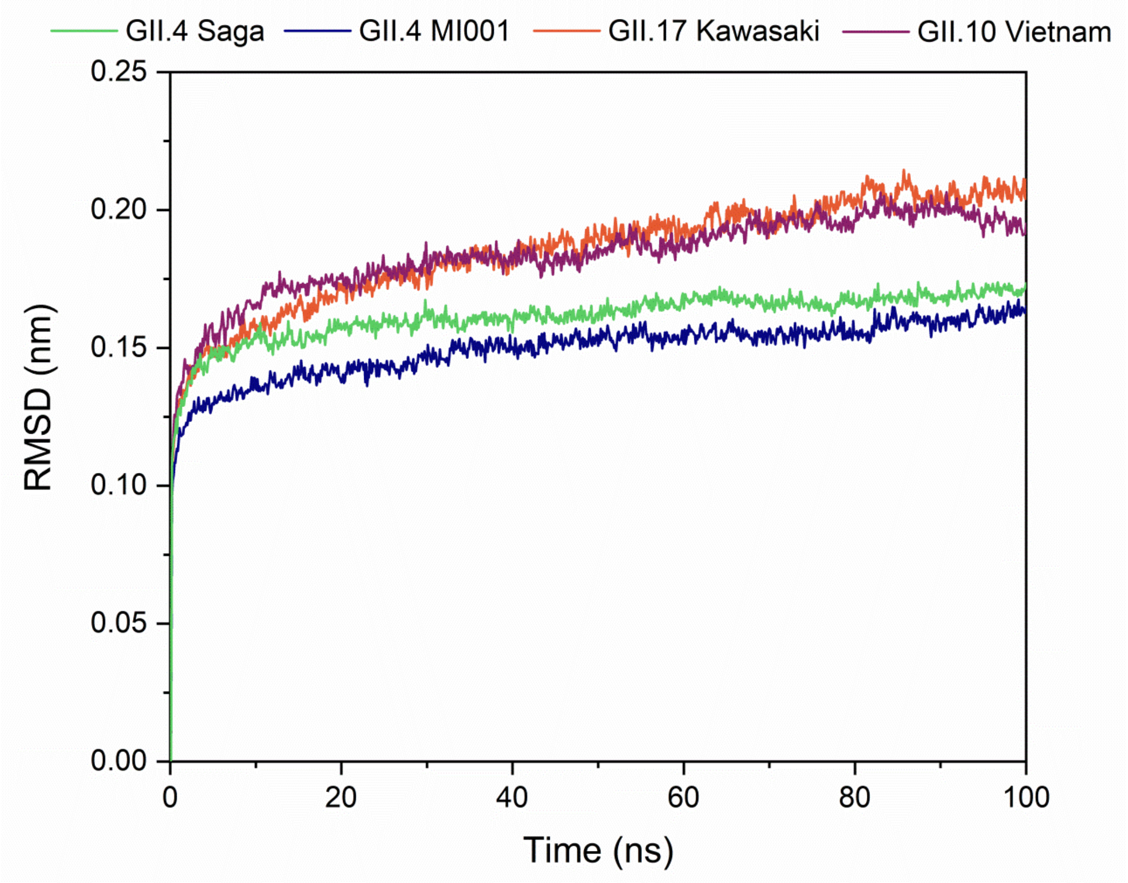

Figure S 15: RMSD of the GII.4 Saga, GII.4 MI001, GII.17 Kawasaki and GII.10 Vietnam P dimer strains.

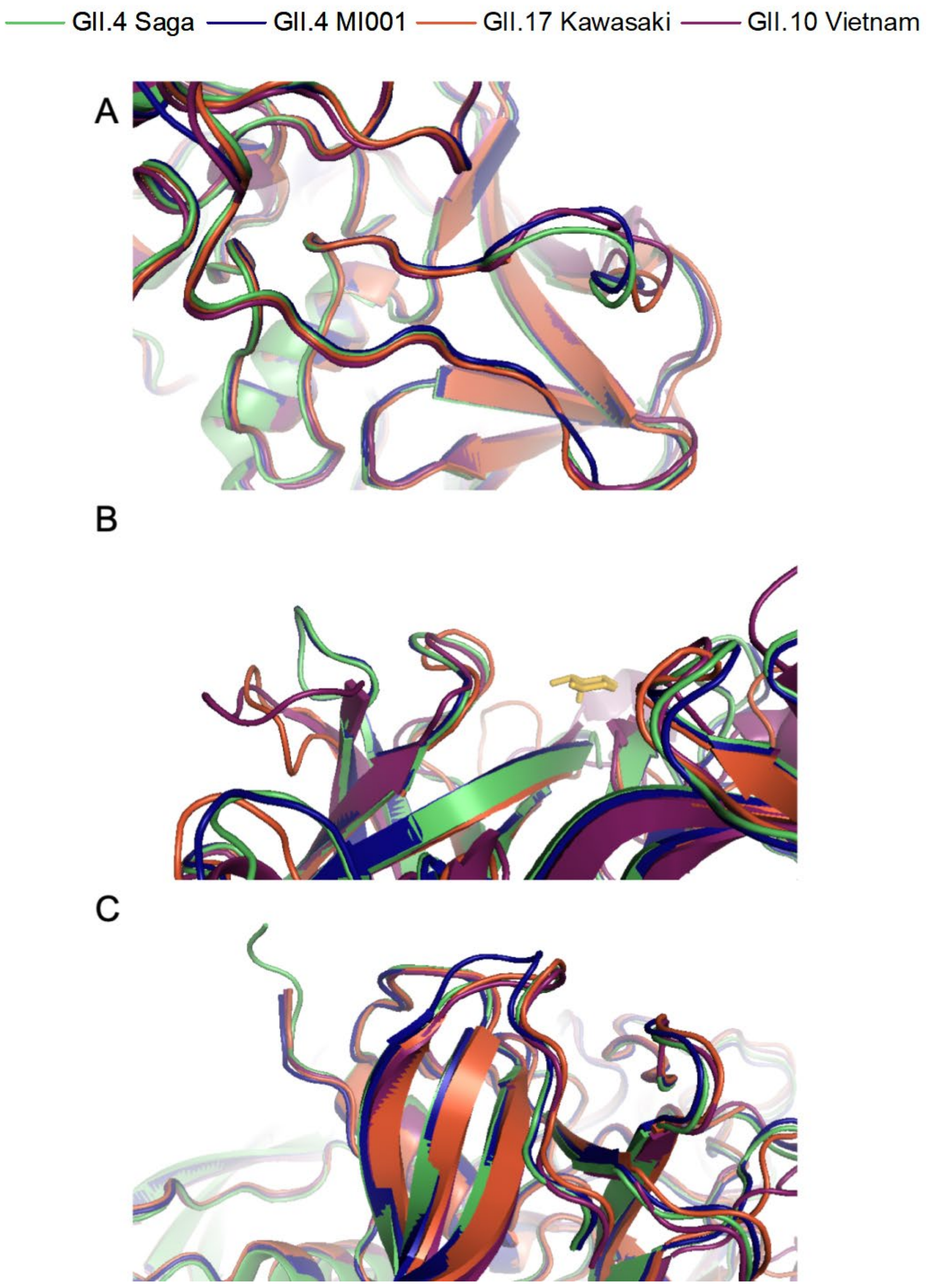

Figure S 16: Overlaid peptide chains of GII.4 Saga, GII.4 MI001, GII.17 Kawasaki and GII.10 Vietnam reveal differences in the peptide chains around residue 250 (A), 300 (B) and 424 (C). These areas of interest were chosen according to higher fluctuation observed in the RMSF data (figure 5 in the main manuscript). Unlike the loop around residue 350 (figure 6 in the main manuscript), which features a small helix in the GII.10 Vietnam strain, conformations depicted in this image show only smaller differences between the different strains. Fucose can be observed in panel B yellow-colored.

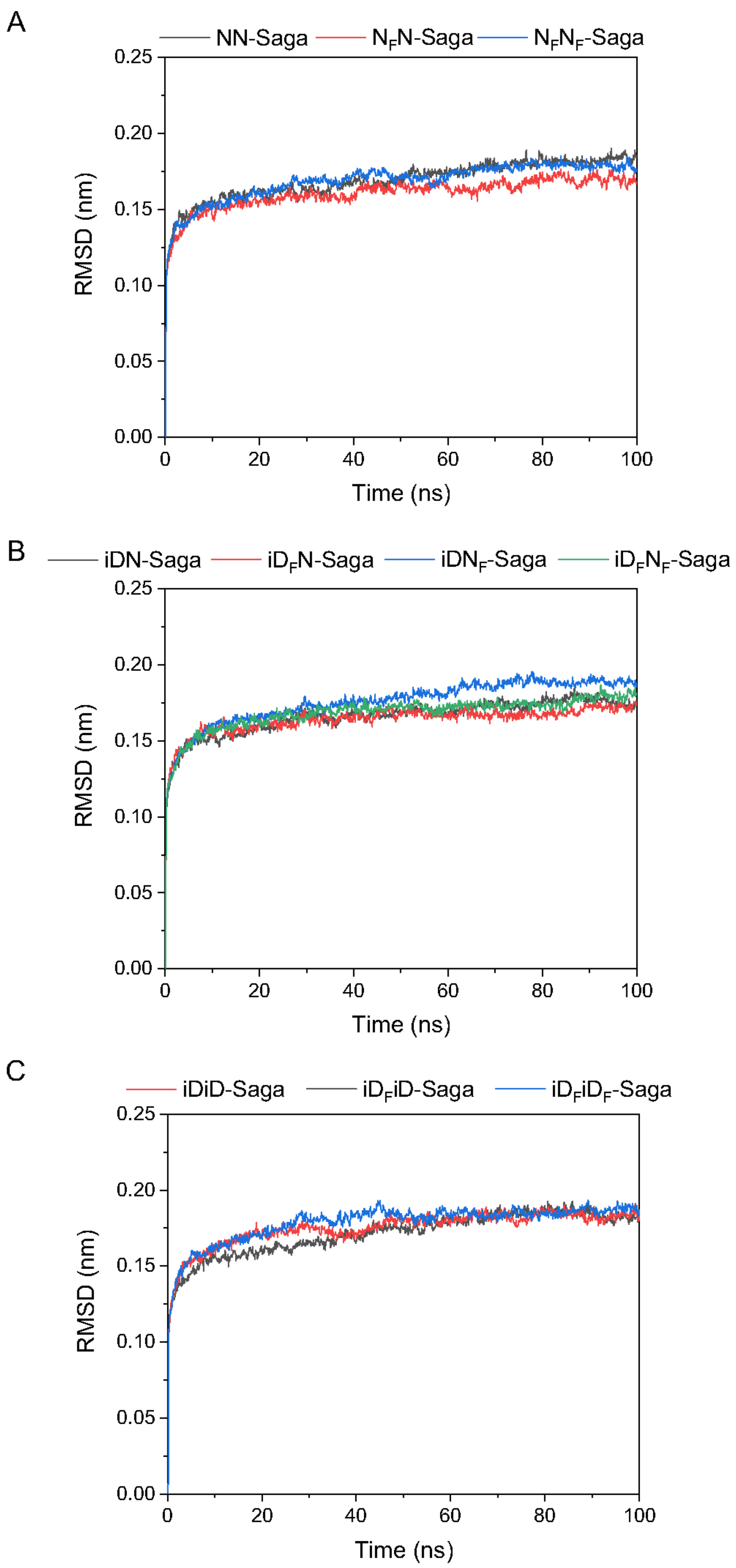

Figure S 17: RMSD of GII.4 Saga NN (A), iDN (B) and iDiD (C) P dimers, pristine and complexed by fucose.

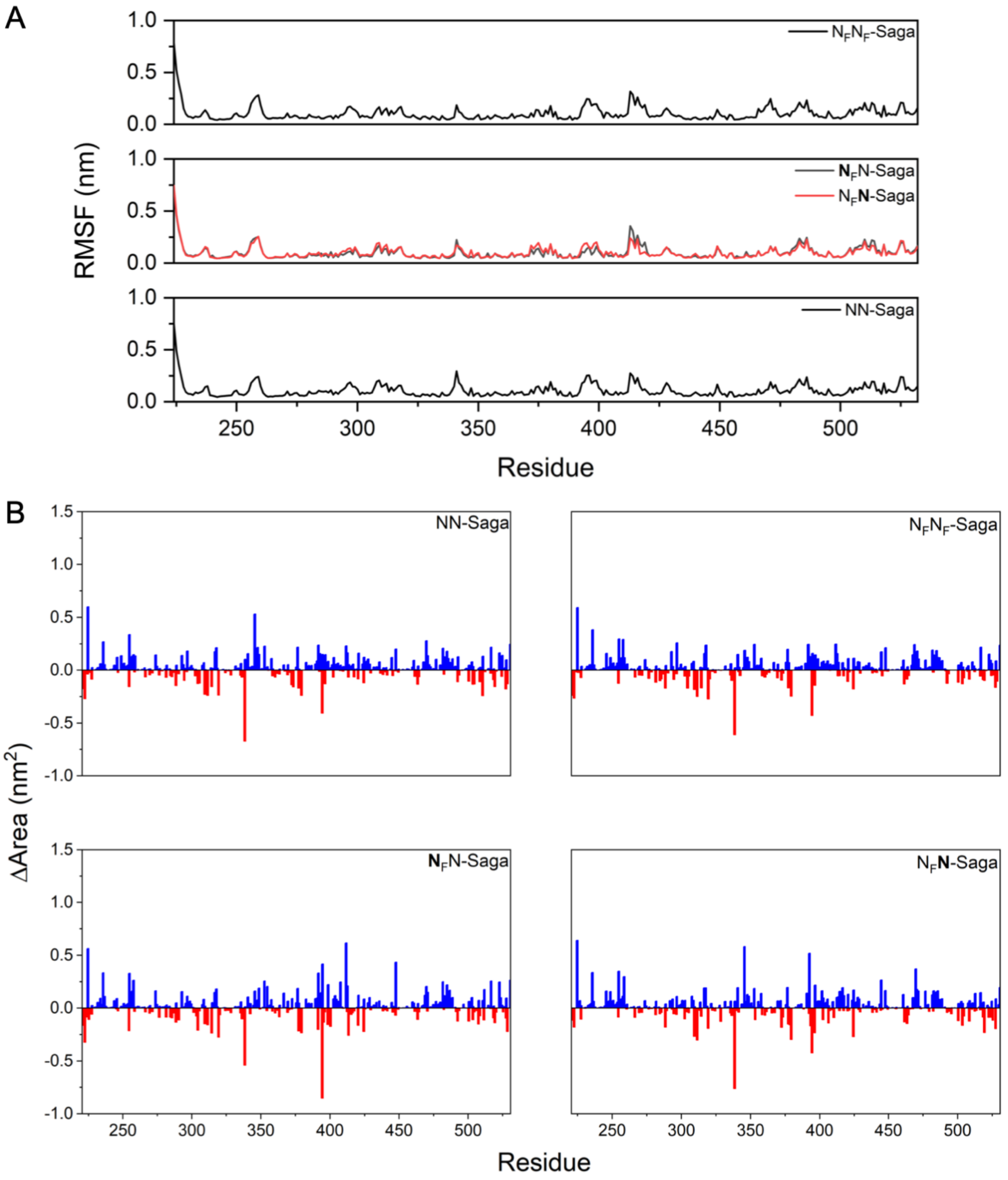

Figure S 18: RMSF (A) and the solvent accessible surface area (B) of NN-Saga without and complexed by fucose. Bold characters in the legends in panel B relate to the protein chain presented by the data.

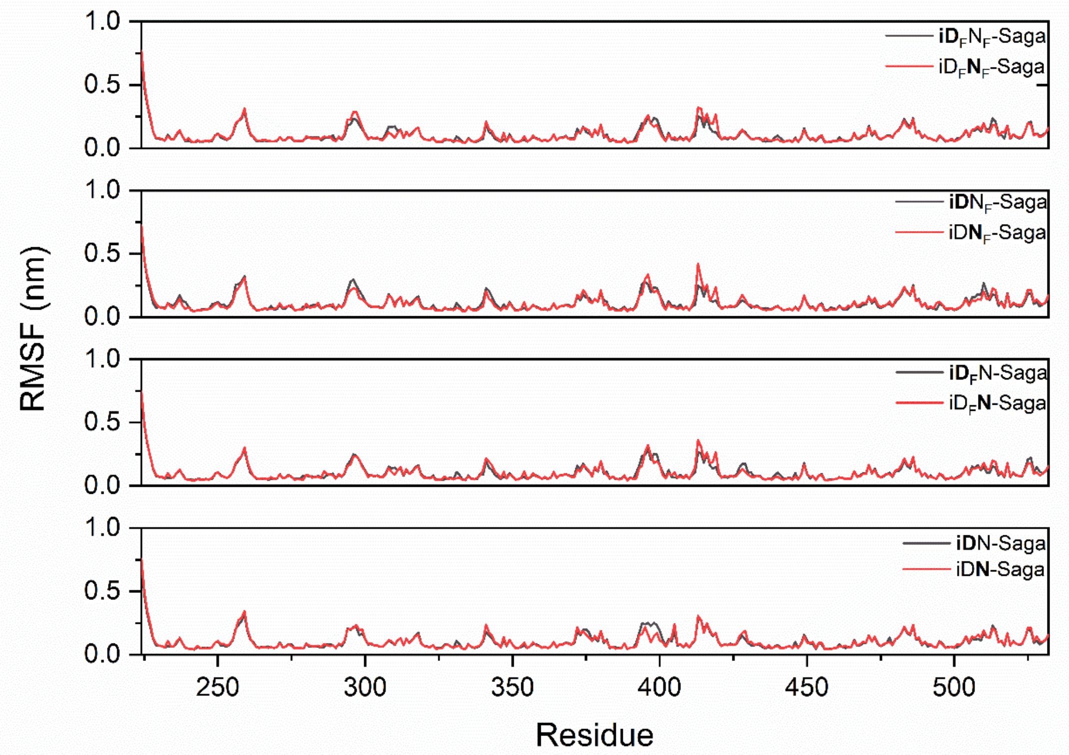

Figure S 19: RMSF (A) and the solvent accessible surface area (B) of iDN-Saga without and complexed by fucose. Bold characters in the legends in panel B relate to the protein chain presented by the data.

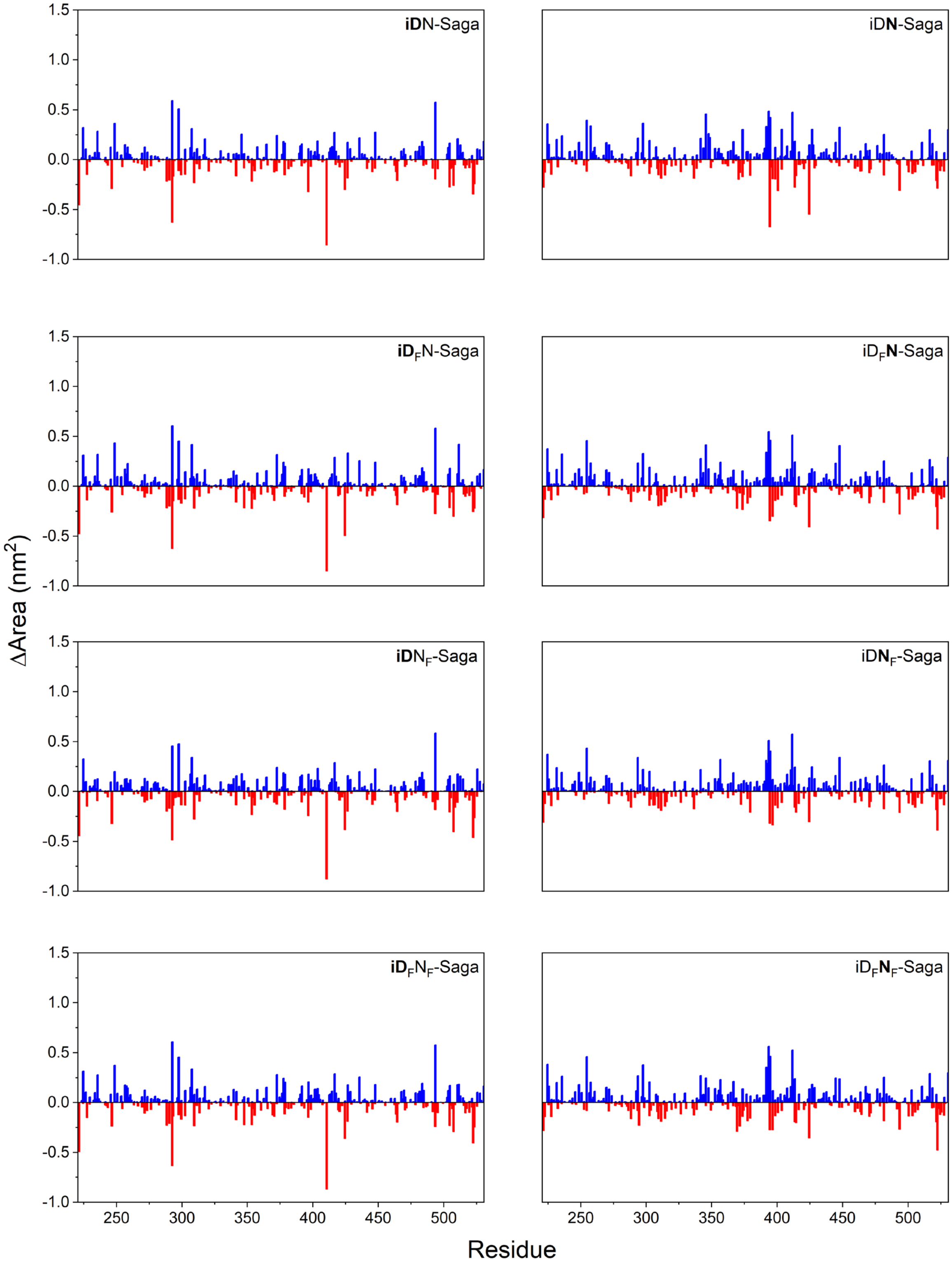

Figure S 19: continued.

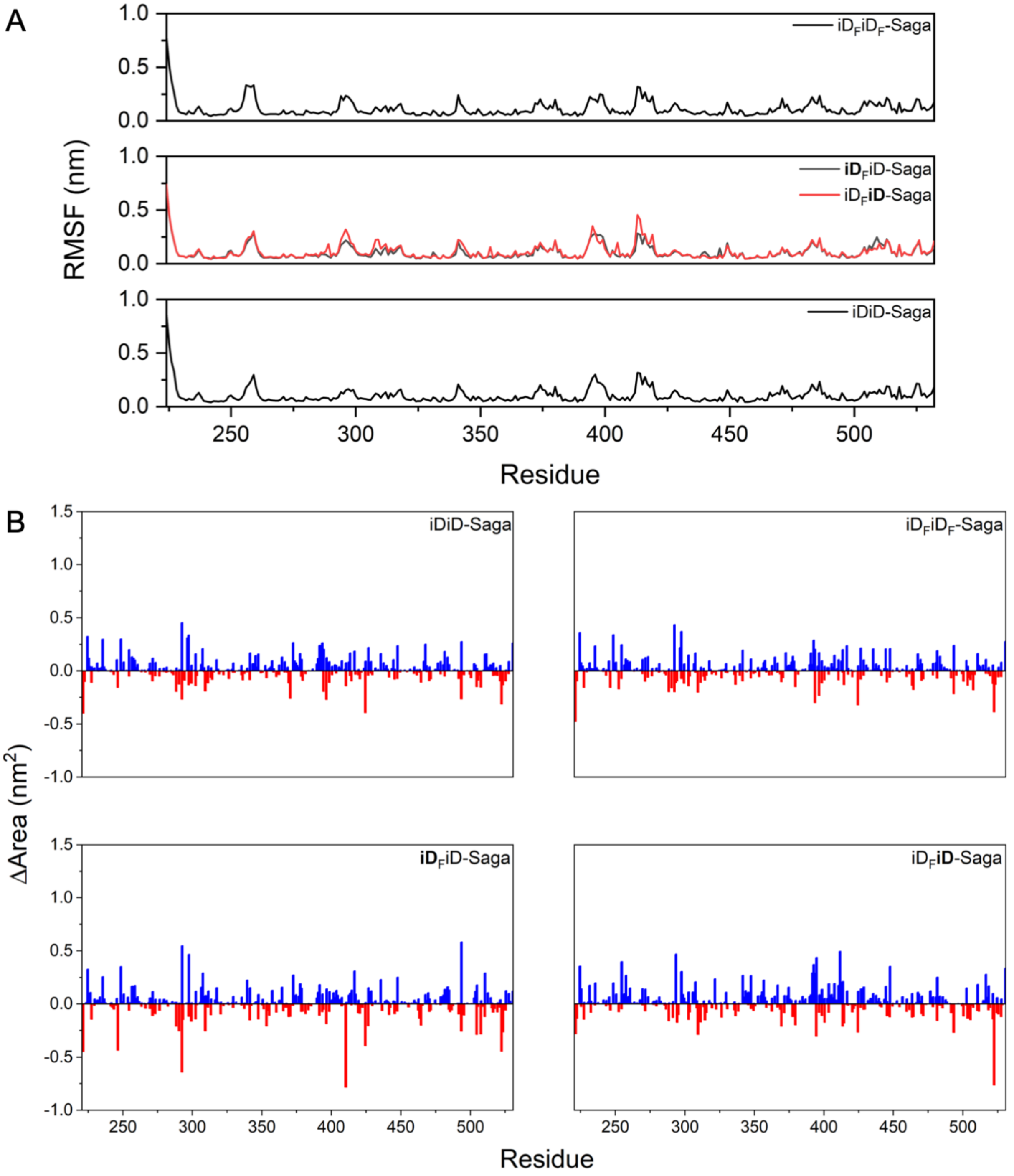

Figure S 20: RMSF (A) and the solvent accessible surface area (B) of iDiD-Saga without and complexed by fucose. Bold characters in the legends in panel B relate to the protein chain presented by the data.

2 HDX figures and tables

2.1 Peptide coverage maps

GII.17 Kawasaki P dimer + 10 mM HBGA B trisaccharide (97 % sequence coverage)

GII.17 Kawasaki P dimer + 100 mM fucose (97 % sequence coverage)

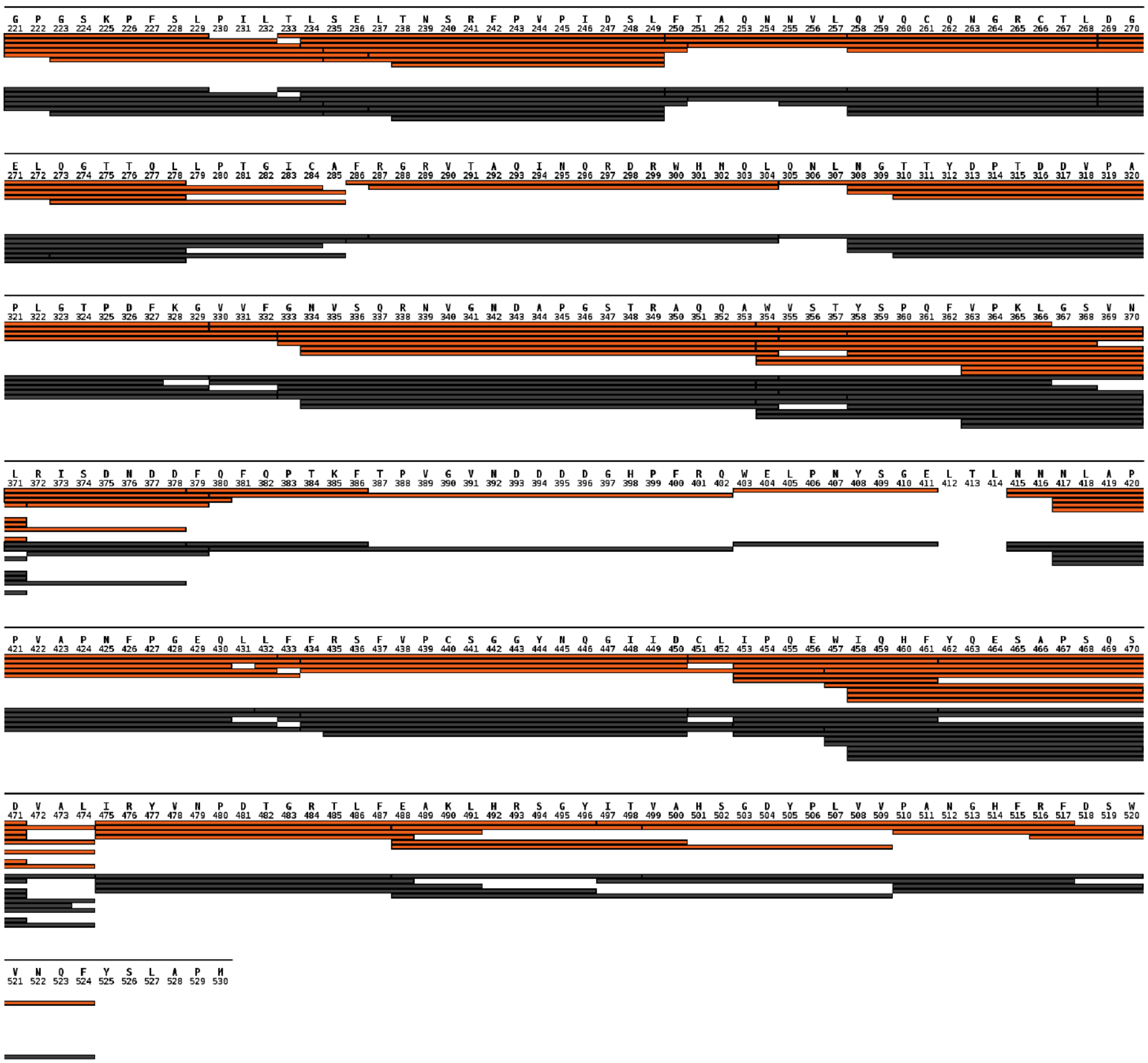

Figure S 21: GII.17 Kawasaki P domain effective peptide coverage map for HDX-MS experiments with HBGA B trisaccharide (orange) or fucose (grey). The N-terminal GPGs sequence was introduced during cloning, so that the first GII.17 P domain residue is K225. Sequence coverage is shown for two individual HDX-MS datasets with either B trisaccharide or fucose. Sequence coverage after peptide identification was 99 %.

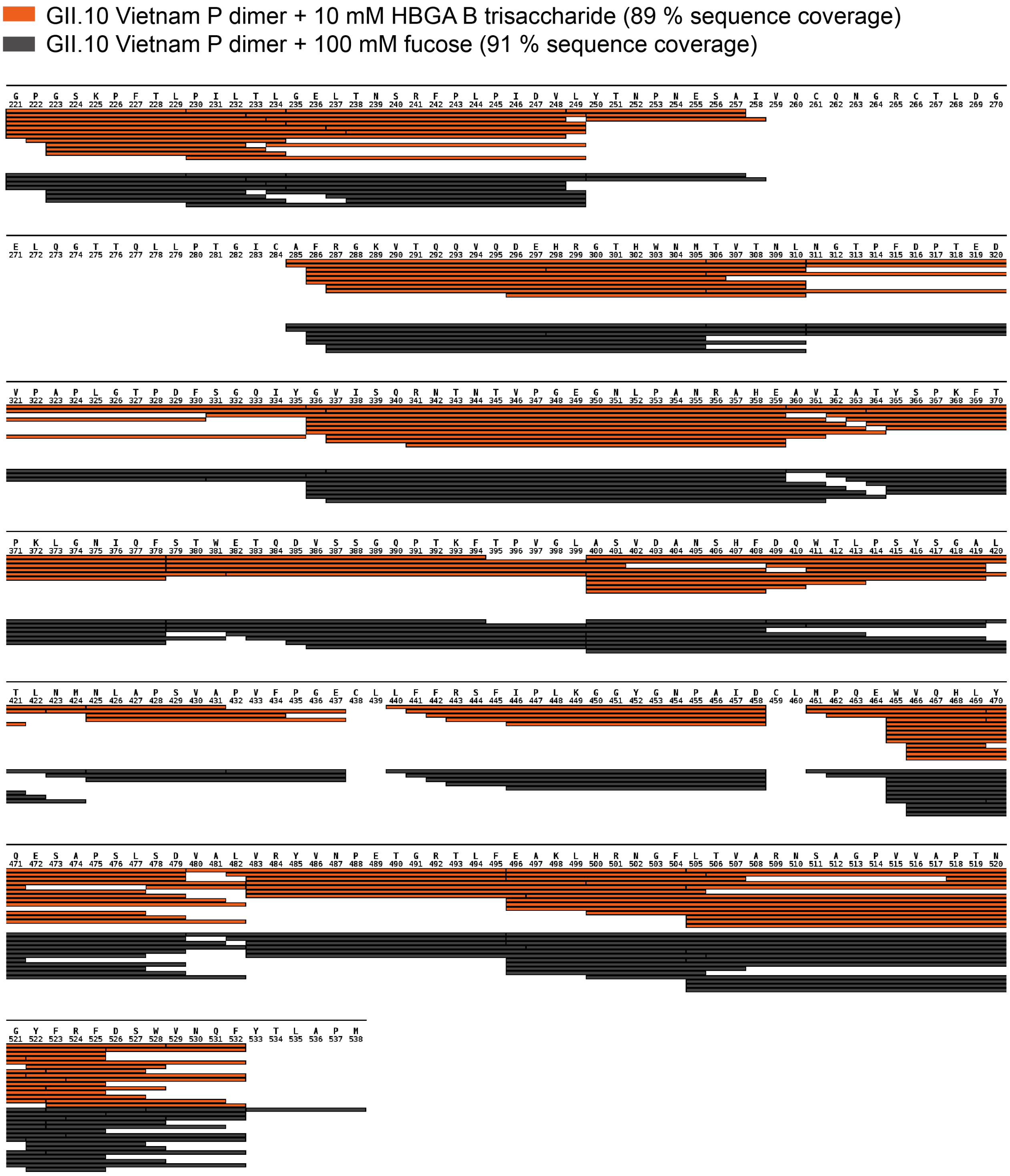

Figure S 22: GII.10 Vietnam P domain effective peptide coverage map for HDX-MS experiments with HBGA B trisaccharide (orange) or fucose (grey). Peptides for region 259-284 were identified in the non-deuterated sample, but data quality in the deuterated samples was insufficient to be included in the HDX MS analysis. The N-terminal GPG sequence was introduced during cloning, so that the first GII.10 P domain residue is S224. Sequence coverage is shown for two individual HDX-MS datasets with either B trisaccharide or fucose. Sequence coverage after peptide identification was 96 %.

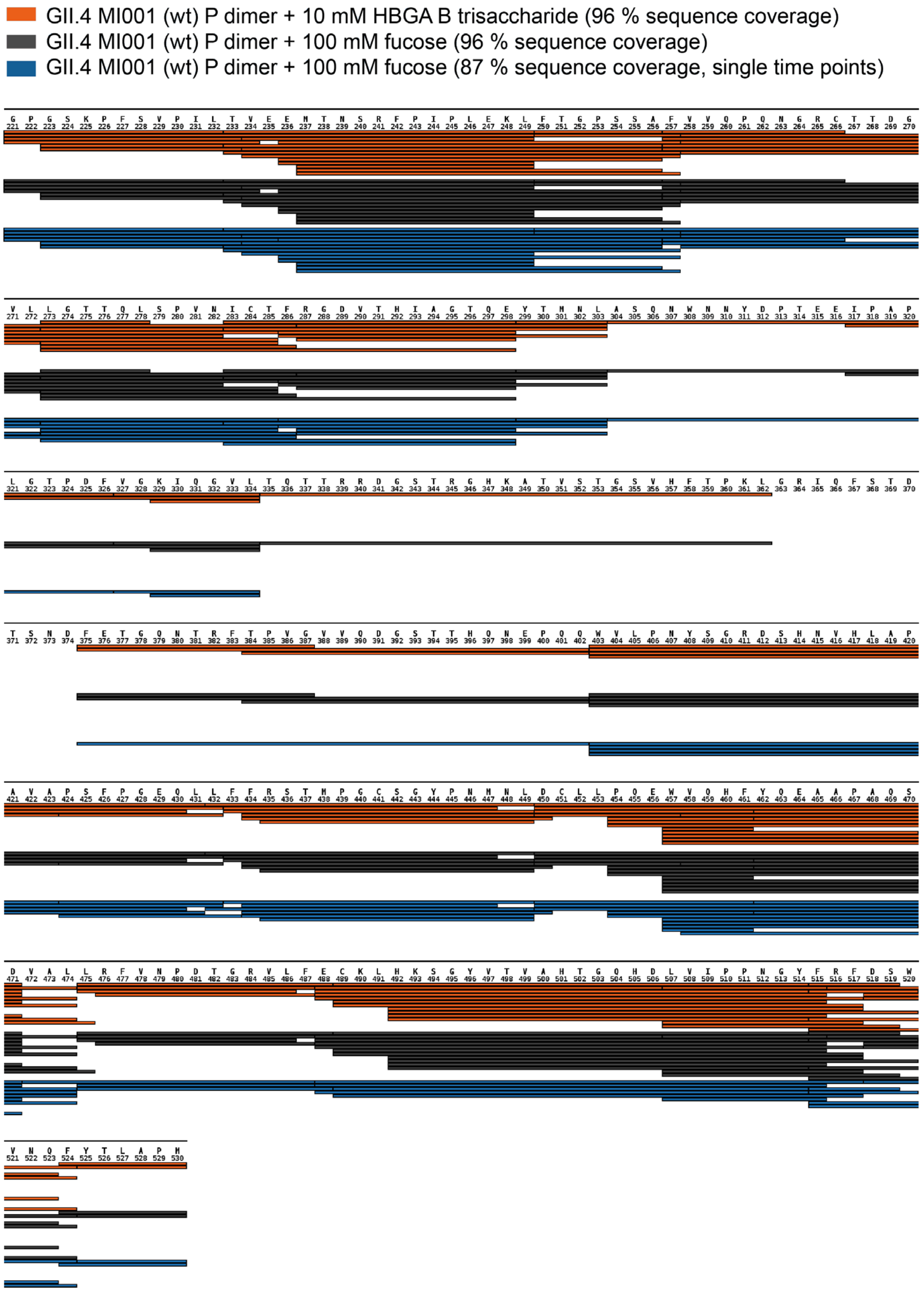

Figure S 23: GII.4 MI001 wildtype P domain effective peptide coverage map for HDX-MS experiments with HBGA B trisaccharide (orange) and fucose (triplicate measurement (grey) or single measurement (blue)). Peptides covering the N373 deamidation site were identified in the non-deuterated sample, but data quality in the deuterated samples was insufficient to be included in the HDX-MS analysis. The N-terminal GPGs sequence was introduced during cloning, so that the first GII.4 P domain residue is K225. Sequence coverage is shown for two individual HDX-MS datasets (triplicate measurement of P dimer + B trisaccharide or fucose, single replicate measurement of P dimer + fucose). Sequence coverage after peptide identification was 100 %.

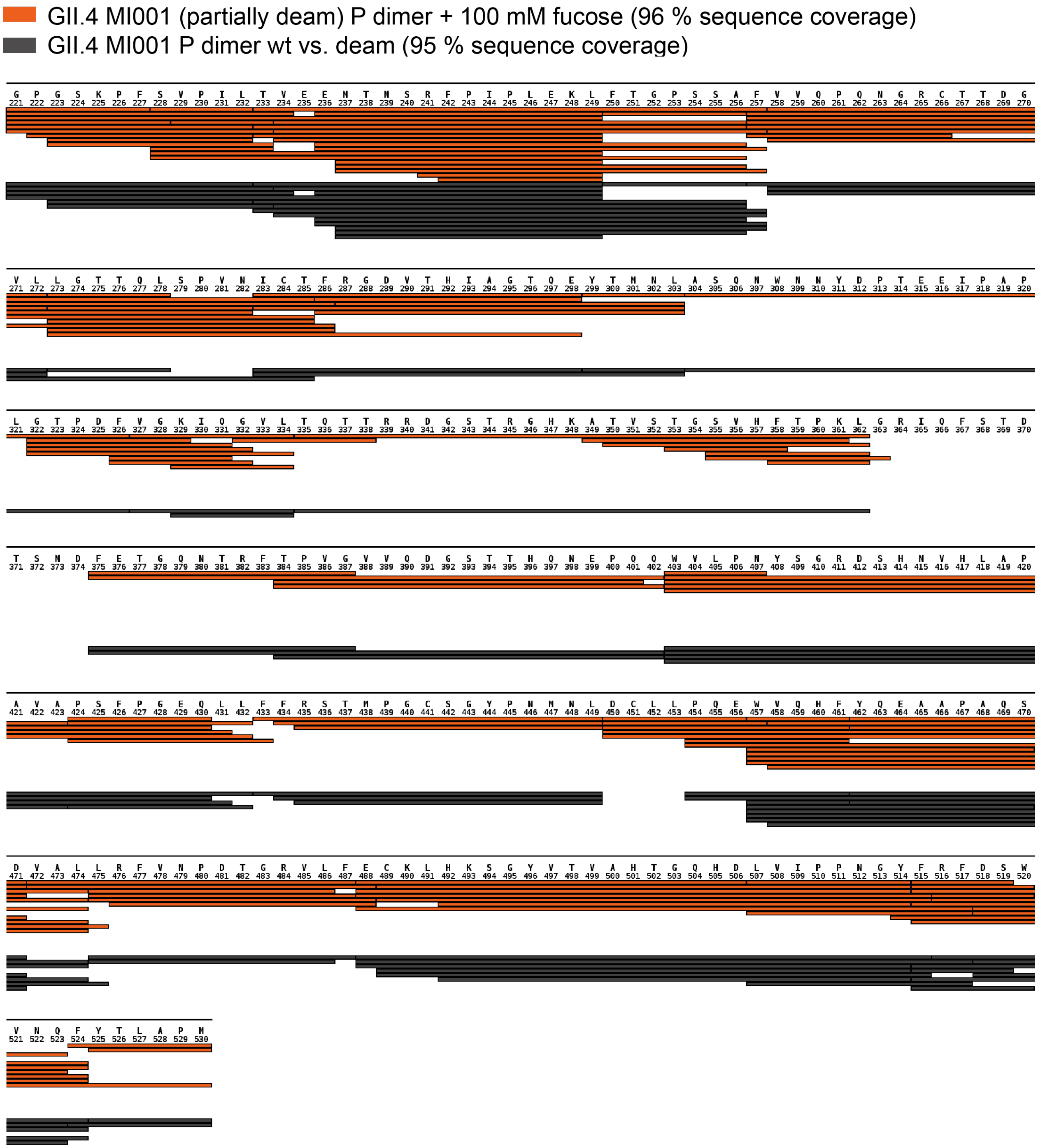

Figure S 24: GII.4 MI001 partially deamidated P domain effective peptide coverage map for HDX-MS experiments with fucose (orange) and a comparison of wild type and partially deamidated P dimer without ligand (grey). Peptides covering the N373 deamidation site were identified in the non-deuterated sample, but data quality in the deuterated samples was insufficient to be included in the HDX-MS analysis. The N-terminal GPGs sequence was introduced during cloning, so that the first GII.4 P domain residue is K225. Sequence coverage is shown for two individual HDX-MS datasets. Sequence coverage after peptide identification was 100 %.

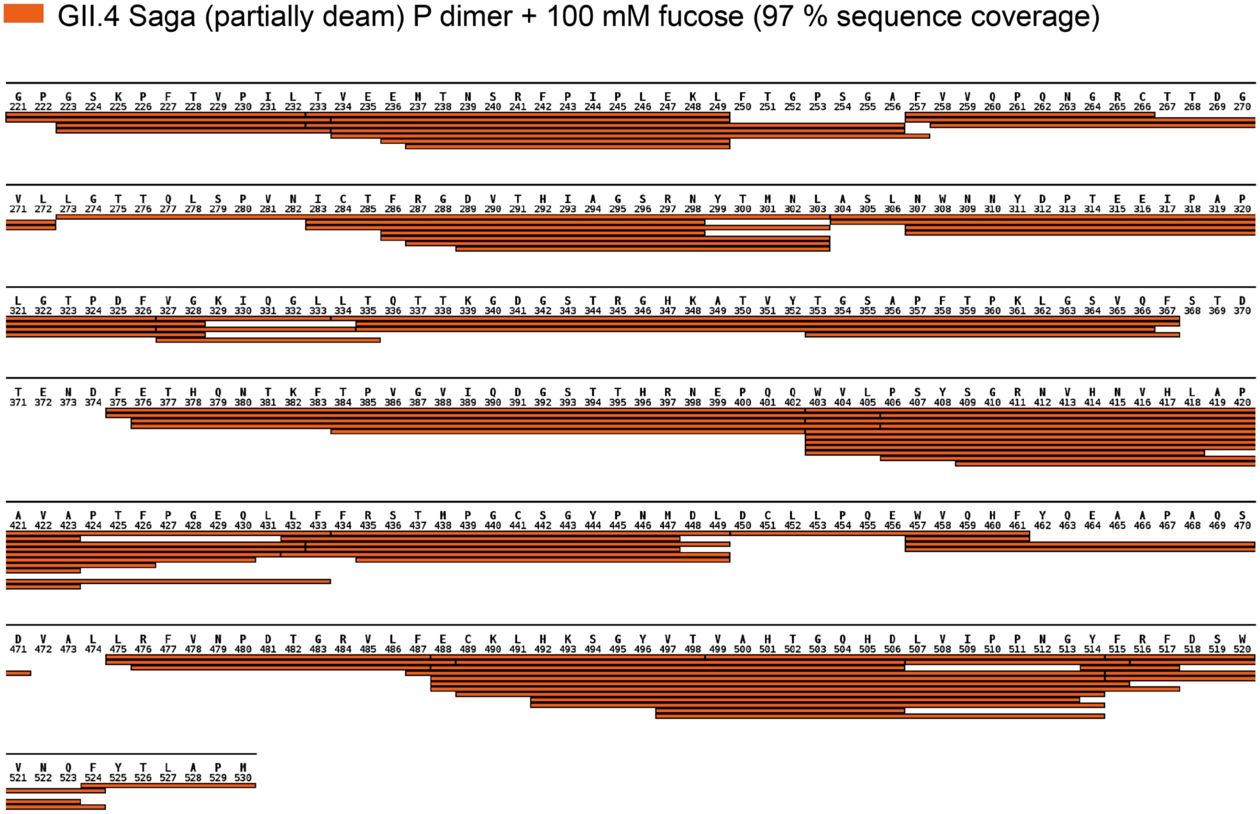

Figure S 25: GII.4 Saga partially deamidated P domain effective peptide coverage map for the HDX-MS experiment with fucose. Peptides covering the N373 deamidation site were identified in the non-deuterated sample, but data quality in the deuterated samples was insufficient to be included in the HDX-MS analysis. The N-terminal GPGs sequence was introduced during cloning, so that the first GII.4 P domain residue is K225. Sequence coverage after peptide identification was 100 %.

2.2 HDX summary tables

Table S 4: HDX summary tables for all datasets according to HDX-MS community recommendations (Masson, G.R., et al., Nat Methods, 2019. **16**(7): p. 595-602).
* Back-exchange values might be overestimated in some datasets due to inconsistencies in P dimer FD sample preparation. Back-exchange from fully deuterated model peptides (T9) provides a more accurrate estimate of back-exchange in the HDX-MS setup.

T1 **GII.10 Vietnam P dimer**

Data Set	unbound	100 mM fucose
HDX reaction details	20 mM Tris, 150 mM NaCl, pH 7, 25 °C	
HDX time course (min)	1, 10, 60, 480	
HDX control samples	FD model peptides in T9	
Back-exchange (mean)	NA	
# of Peptides	117	117
Sequence coverage	91%	91%
Average peptide length / Redundancy	15.9 / 5.8	15.9 / 5.8
Replicates (biological or technical)	3 (technical)	3 (technical)
Repeatability (average standard deviation)	0.1	0.073
Significant differences in HDX (delta HDX > X D)	T test (p<0.05) and delta D > 0.25 (2x pooled average SD), delta D > 0.55 (99% percentile) for 8h time point single measurement, manual validation in case of bimodality	

T2 **GII.10 Vietnam P dimer**

Data Set	unbound	10 mM HBGA B trisaccharide
HDX reaction details	20 mM Tris, 150 mM NaCl, pH 7, 25 °C	
HDX time course (min)	1, 10, 60, 480	
HDX control samples	Fully deuterated protein control, labeled for 24 h, buffer 20 mM Tris, 6 M urea, pH 7 + FD model peptides in T9	
Back-exchange (mean)	50 ± 15 % (FD protein)*	
# of Peptides	126	126
Sequence coverage	89%	89%
Average peptide length / Redundancy	16.4 / 6.49	16.4 / 6.49
Replicates (biological or technical)	3 (technical)	3 (technical)
Repeatability (average standard deviation)	0.12	0.178
Significant differences in HDX (delta HDX > X D)	T test (p<0.05) and delta D > 0.43 (2x pooled average SD), manual validation in case of bimodality	

T3 **GII.17 Kawasaki P dimer**

Data Set	unbound	100 mM fucose
HDX reaction details	20 mM Tris, 150 mM NaCl, pH 7, 25 °C	
HDX time course (min)	1, 10, 60, 480	
HDX control samples	Fully deuterated protein control, labeled for 72 h, buffer 20 mM Tris, 6 M urea, pH 7 + FD model peptides in T9	
Back-exchange (mean)	64 ± 11 % (FD protein)*	
# of Peptides	89	89
Sequence coverage	97%	97%
Average peptide length / Redundancy	16.3 / 4.69	16.3 / 4.69
Replicates (biological or technical)	3 (technical)	3 (technical)
Repeatability (average standard deviation)	0.057	0.083
Significant differences in HDX (delta HDX > X D)	T test (p<0.05) and delta D > 0.20 (2x pooled average SD), manual validation in case of bimodality	

T4 **GII.17 Kawasaki P dimer**

Data Set	unbound	10 mM HBGA B trisasccharide
HDX reaction details	20 mM Tris, 150 mM NaCl, pH 7, 25 °C	
HDX time course (min)	1, 10, 60, 480	
HDX control samples	Fully deuterated protein control, labeled for 72 h, buffer 20 mM Tris, 6 M urea, pH 7 + FD model peptides in T9	
Back-exchange (mean)	58 ± 14 % (FD protein)*	
# of Peptides	84	84
Sequence coverage	97%	97%
Average peptide length / Redundancy	16.1 / 4.36	16.1 / 4.36
Replicates (biological or technical)	3 (technical)	3 (technical)
Repeatability (average standard deviation)	0.115	0.157
Significant differences in HDX (delta HDX > X D)	T test (p<0.05) and delta D > 0.39 (2x pooled average SD), manual validation in case of bimodality	

T5 **GII.4 MI001 P dimer wildtype**

Data Set	unbound	10 mM HBGA B trisasccharide
HDX reaction details	20 mM Tris, 150 mM NaCl, pH 7, 25 °C	
HDX time course (min)	1, 10, 60, 480	
HDX control samples	Fully deuterated protein control, labeled for 72 h, buffer 20 mM Tris, 6 M urea, pH 7 + FD model peptides in T9	
Back-exchange (mean)	47 ± 12 % (FD protein)*	
# of Peptides	100	100
Sequence coverage	96%	96%
Average peptide length / Redundancy	16.9 / 5.43	16.9 / 5.43
Replicates (biological or technical)	3 (technical)	3 (technical)
Repeatability (average standard deviation)	0.08	0.073
Significant differences in HDX (delta HDX > X D)	T test (p<0.05) and delta D > 0.22 (2x pooled average SD), manual validation in case of bimodality	

Data Set	unbound	100 mM fucose
HDX reaction details	20 mM Tris, 150 mM NaCl, pH 7, 25 °C	
HDX time course (min)	1, 10, 60, 480	
HDX control samples	Fully deuterated protein control, labeled for 72 h, buffer 20 mM Tris, 6 M urea, pH 7 + FD model peptides in T9	
Back-exchange (mean)	47 ± 12 % (FD protein)*	
# of Peptides	100	100
Sequence coverage	96%	96%
Average peptide length / Redundancy	16.9 / 5.43	16.9 / 5.43
Replicates (biological or technical)	3 (technical)	3 (technical)
Repeatability (average standard deviation)	0.08	0.09
Significant differences in HDX (delta HDX > X D)	T test (p<0.05) and delta D > 0.23 (2x pooled average SD), manual validation in case of bimodality	

T6 **GII.4 MI001 P dimer wildtype**

Data Set	unbound	100 mM fucose
HDX reaction details	20 mM Tris, 150 mM NaCl, pH 7, 25 °C	
HDX time course (min)	0.25, 1, 10, 60, 480	
HDX control samples	Fully deuterated protein control, labeled for 24 h, buffer 20 mM Tris, 6 M urea, pH 7 + FD model peptides in T9	
Back-exchange (mean)	40 ± 8 % (FD protein)*	
# of Peptides	81	81
Sequence coverage	87%	87%
Average peptide length / Redundancy	16.6 / 4.34	16.6 / 4.34
Replicates (biological or technical)	1 (technical)	1 (technical)
Repeatability (average standard deviation)	0.1 for FD (2 technical replicates)	-
Significant differences in HDX (delta HDX > X D)	delta D > 0.5 (99% percentile of dataset T5 with similar SD), manual validation in case of bimodality	

T7 **GII.4 MI001 P dimer partially deamidated**

Data Set	unbound	100 mM fucose
HDX reaction details	20 mM Tris, 150 mM NaCl, pH 7, 25 °C	
HDX time course (min)	1, 10, 60, 480	
HDX control samples	Fully deuterated protein control, labeled for 72 h, buffer 20 mM Tris, 6 M urea, pH 7 + FD model peptides in T9	
Back-exchange (mean)	41 ± 13 % (FD protein)*	
# of Peptides	123	123
Sequence coverage	96%	96%
Average peptide length / Redundancy	15.0 / 5.94	15.0 / 5.94
Replicates (biological or technical)	3 (technical)	3 (technical)
Repeatability (average standard deviation)	0.074	0.079
Significant differences in HDX (delta HDX > X D)	T test (p<0.05) and delta D > 0.21 (2x pooled average SD), manual validation in case of bimodality	

T8 **GII.4 MI001 P dimer wildtype vs. partially deamidated**

Data Set	unbound (wt) (T5)	unbound (deam) (T7)
HDX reaction details	20 mM Tris, 150 mM NaCl, pH 7, 25 °C	
HDX time course (min)	1, 10, 60, 480	
HDX control samples	fully deuterated protein control (FD T5 and T7) + FD model peptides in T9	
Back-exchange (mean)	see T5 and T7 for FD protein	
# of Peptides	69	69
Sequence coverage	95%	95%
Average peptide length / Redundancy	16.4 / 3.65	16.4 / 3.65
Replicates (biological or technical)	3 (technical)	3 (technical)
Repeatability (average standard deviation)	0.081	0.072
Significant differences in HDX (delta HDX > X D)	delta D normalized with FD controls ratio, T test (p<0.05) and delta D > 0.42 (99% percentile)	

T9 **fully deuterated model peptides for HDX workflow quality control**

Data Set	Angiotensin I	Bradykinin
HDX reaction details	20 mM deuterated Tris, 6 M GndHCl, pH 7, 25 °C, labeled for 24 h	
HDX time course (min)	-	
HDX control samples	fully deuterated peptide mix serves as general back exchange control for the standard bottom-up HDX MS workflow	
Back-exchange (mean)	23 ± 1% (FD peptide)	32 ± 2 % (FD peptide)
# of Peptides	1	1
Sequence coverage	100%	100%
Average peptide length / Redundancy	10	9
Replicates (biological or technical)	2 (technical)	2 (technical)
Repeatability (average standard deviation)	0.1	0.08
Significant differences in HDX (delta HDX > X D)	-	

T10 **GII.4 Saga P dimer partially deamidated**

Data Set	unbound	100 mM fucose

HDX reaction details	20 mM Tris, 150 mM NaCl, pH 7, 25 °C	
HDX time course (min)	0.25. 1, 10	
HDX control samples	Fully deuterated protein control, labeled for 24 h, buffer 20 mM Tris, 6 M urea, pH 7 + FD model peptides in T9	
Back-exchange (mean)	37 ± 9 % (FD protein)*	
# of Peptides	78	78
Sequence coverage	97%	97%
Average peptide length / Redundancy	18.2 / 4.59	18.2 / 4.59
Replicates (biological or technical)	3 (technical)	3 (technical)
Repeatability (average standard deviation)	0.084	0.109
Significant differences in HDX (delta HDX > X D)	T test (p<0.05) and delta D > 0.27 (2x pooled average SD)	

2.3 MS-Viewer search keys

Table S 5: MS-Viewer search keys. Annotated fragment ion spectra of identified peptides can be viewed in the MS-Viewer online tool at http://msviewer.ucsf.edu/prospector/cgi-bin/msform.cgi?form=msviewer

**Protein**	**Deamidation status**	**MS-Viewer search key**
GII.4 MI001 P domain	wildtype	widghx5vtx
GII.4 MI001 P domain	partially deamidated	svwlky0tqx
GII.4 Saga P domain	partially deamidated	adolnt1njh
GII.10 Vietnam 026	wildtype	ycj8werllz
GII.17 Kawasaki 308	wildtype	9n5zuotk1a

2.4 Deuterium uptake plots

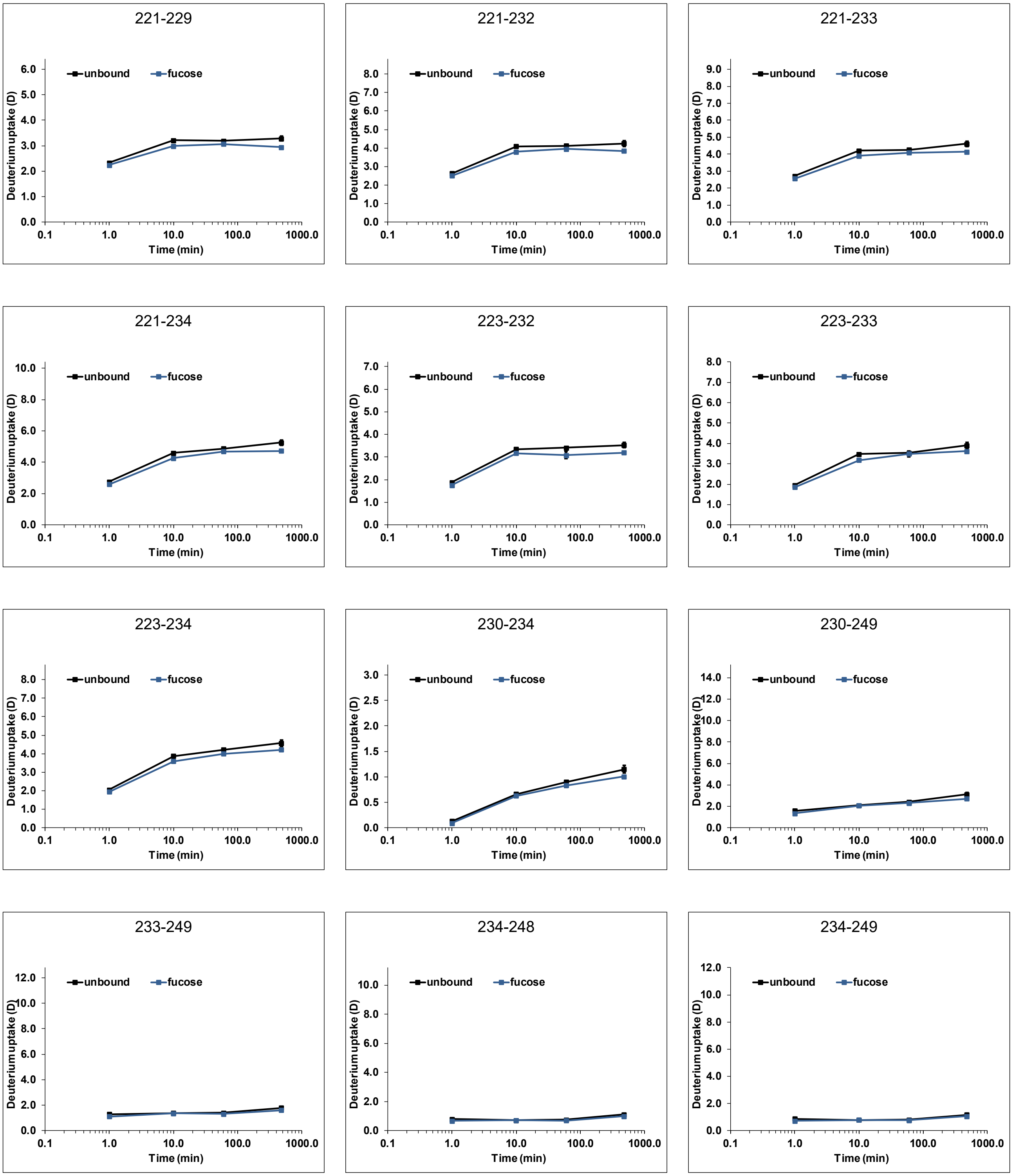

Figure S 26: Centroid deuterium uptake plots for Vietnam P dimers (black) with 100 mM fucose (blue) (dataset T1). Time points 1 min, 10 min, and 1 h were performed in triplicate, the 8 h time point with fucose represents a single measurement. Error bars indicate the standard deviation (SD) of each triplicate analysis. Deuteration values can be falsely low in centroid analysis of bimodal spectra. Compare Figure S 13 and Table S 3 for assessment of statistically significant deuteration differences.

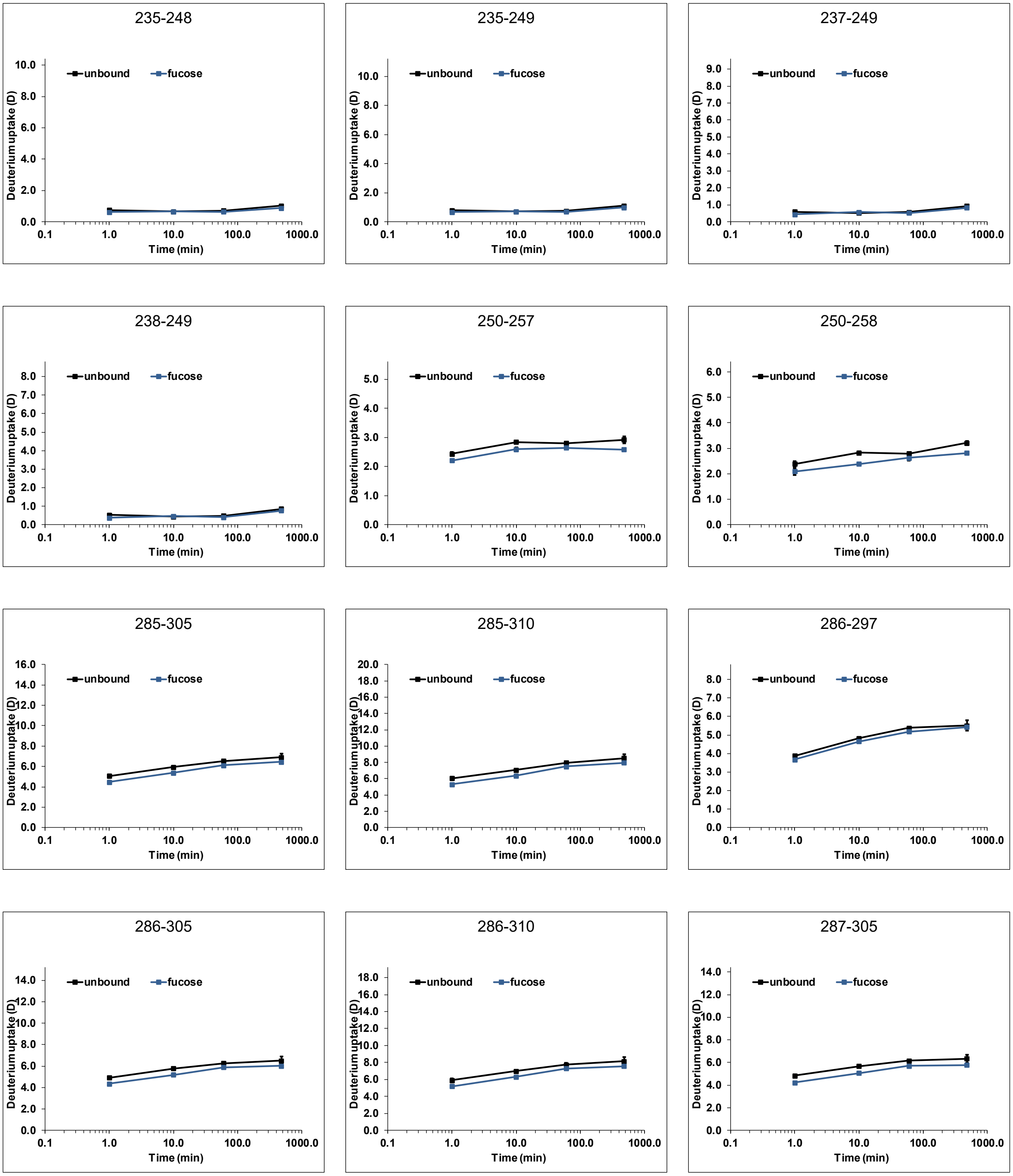

Figure S 26: continued

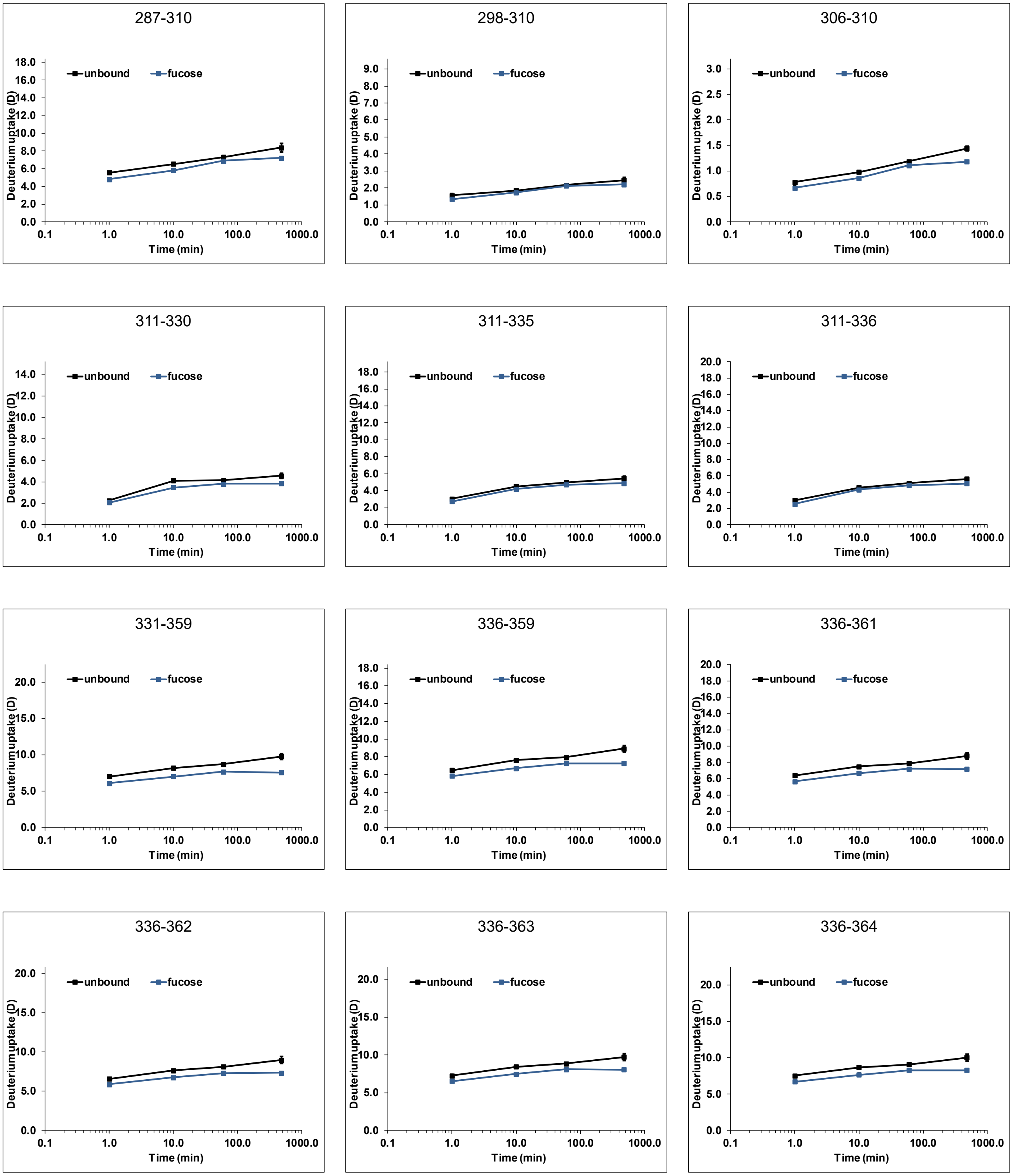

Figure S 26: continued

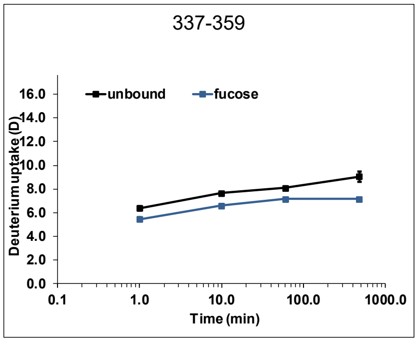

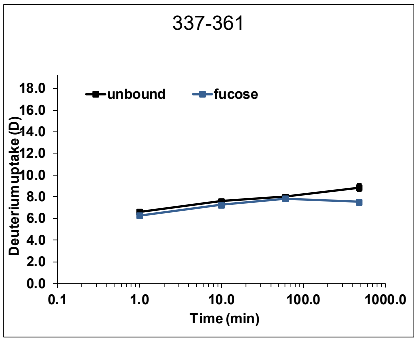

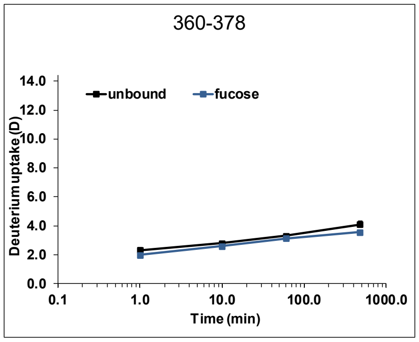

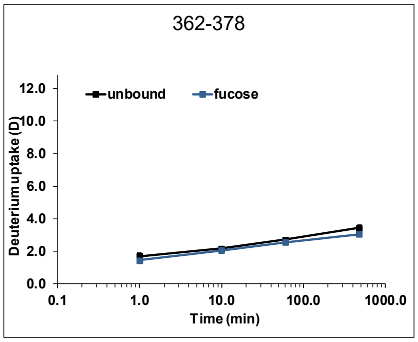

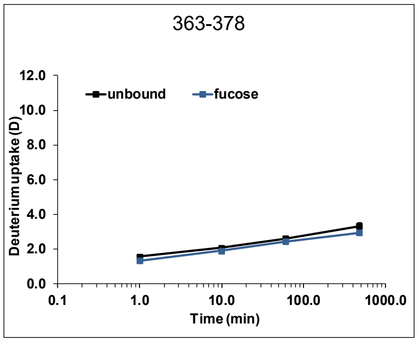

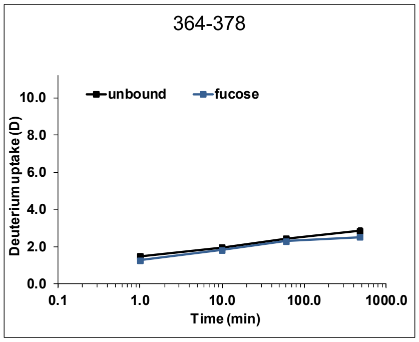

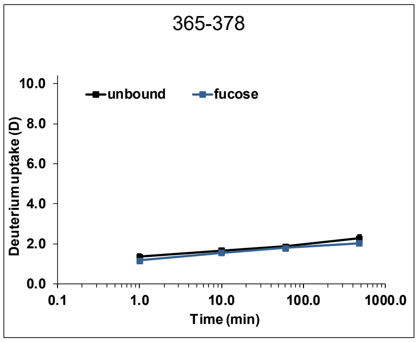

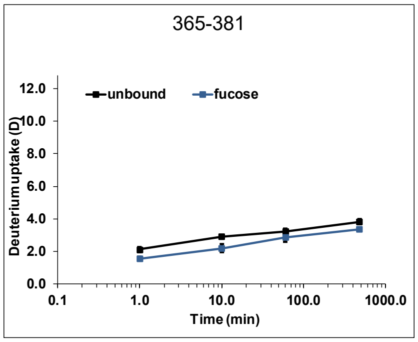

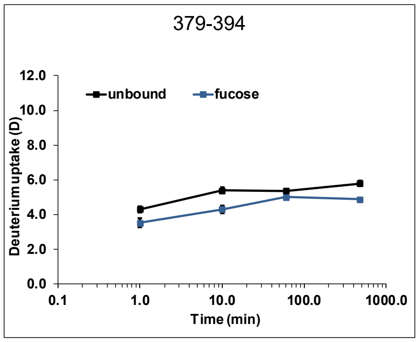

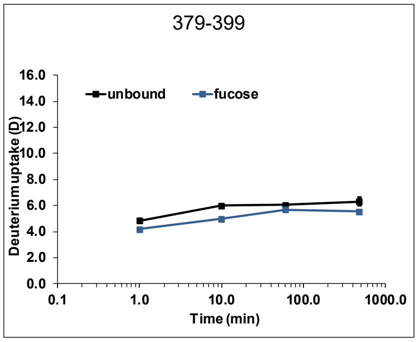

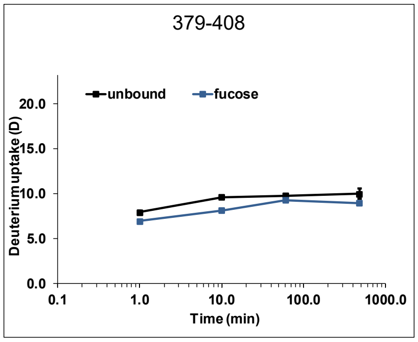

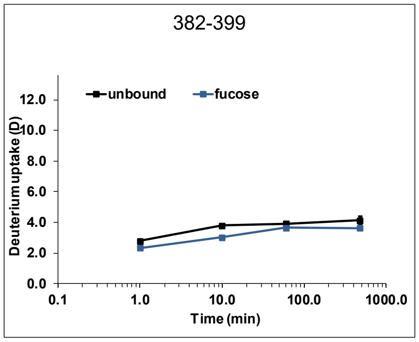

Figure S 26: continued

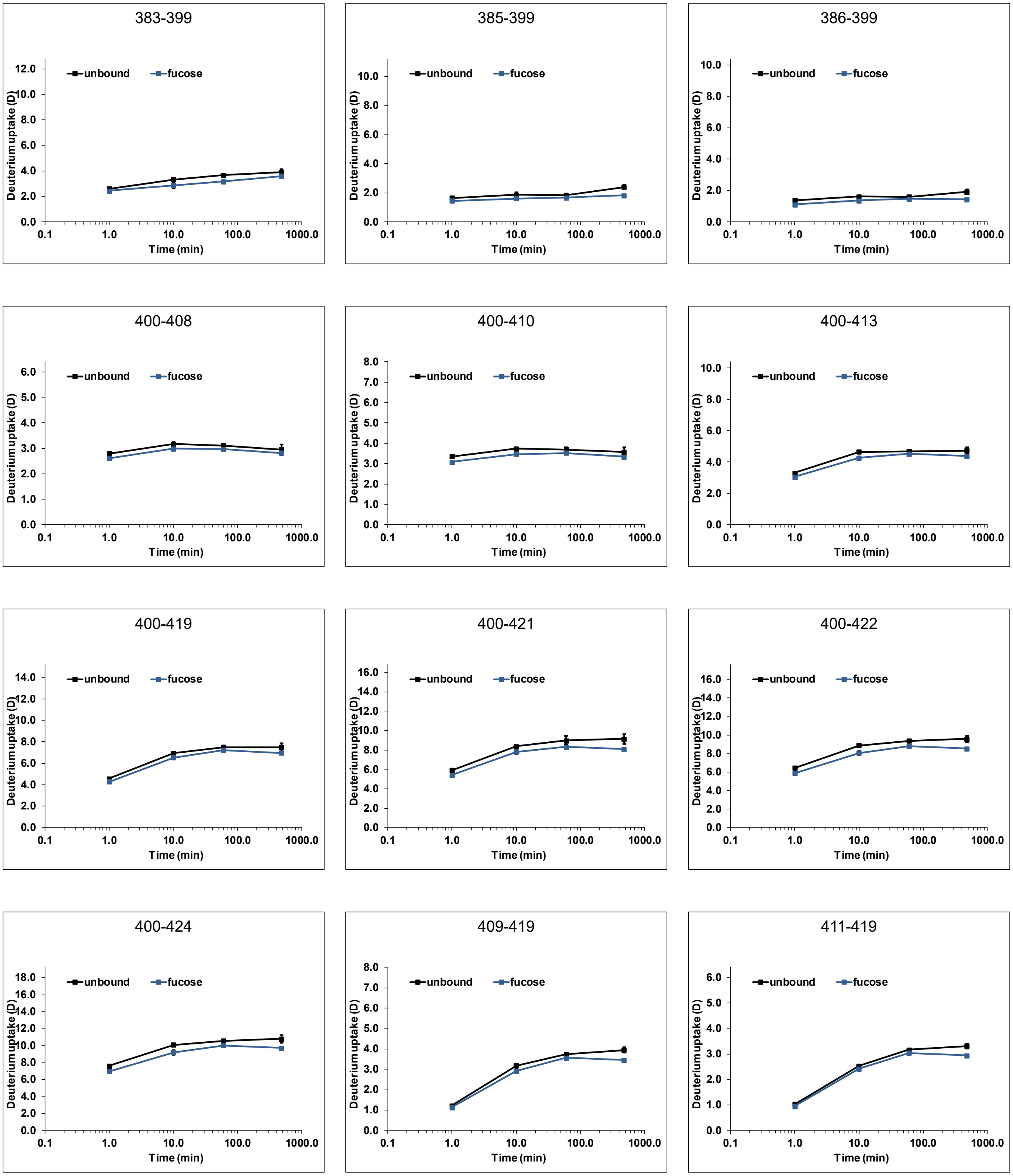

Figure S 26: continued

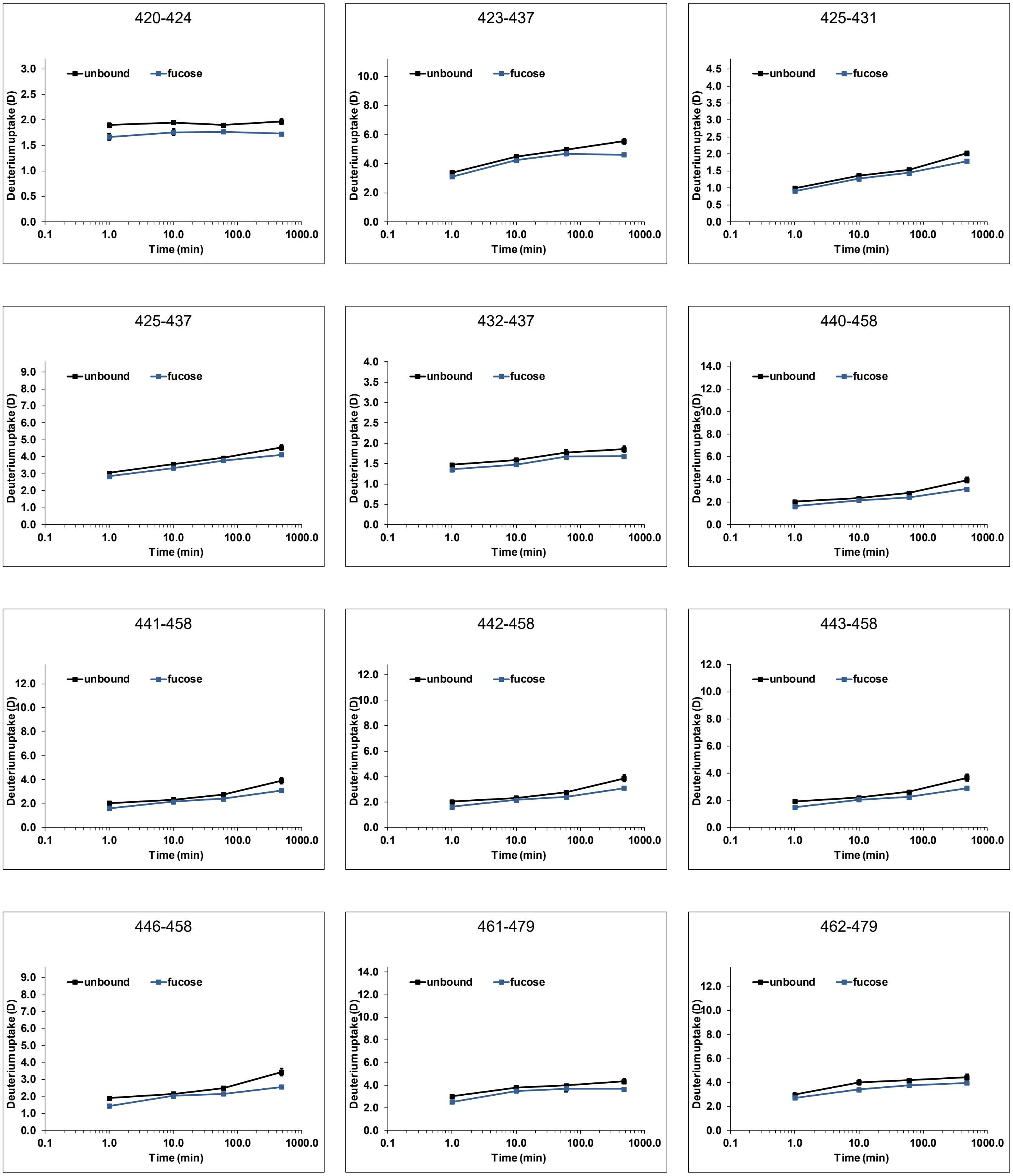

Figure S 26: continued

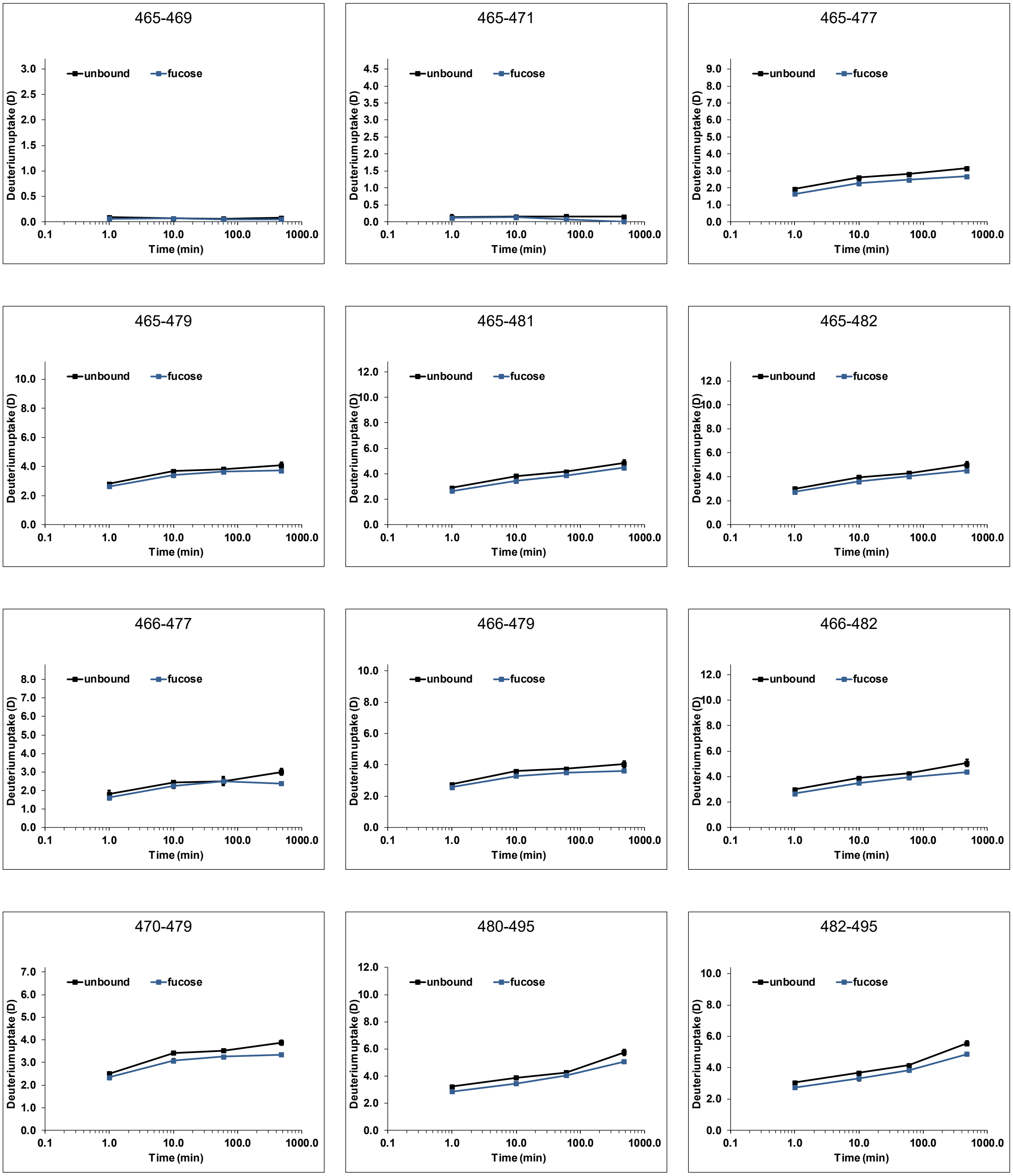

Figure S 26: continued

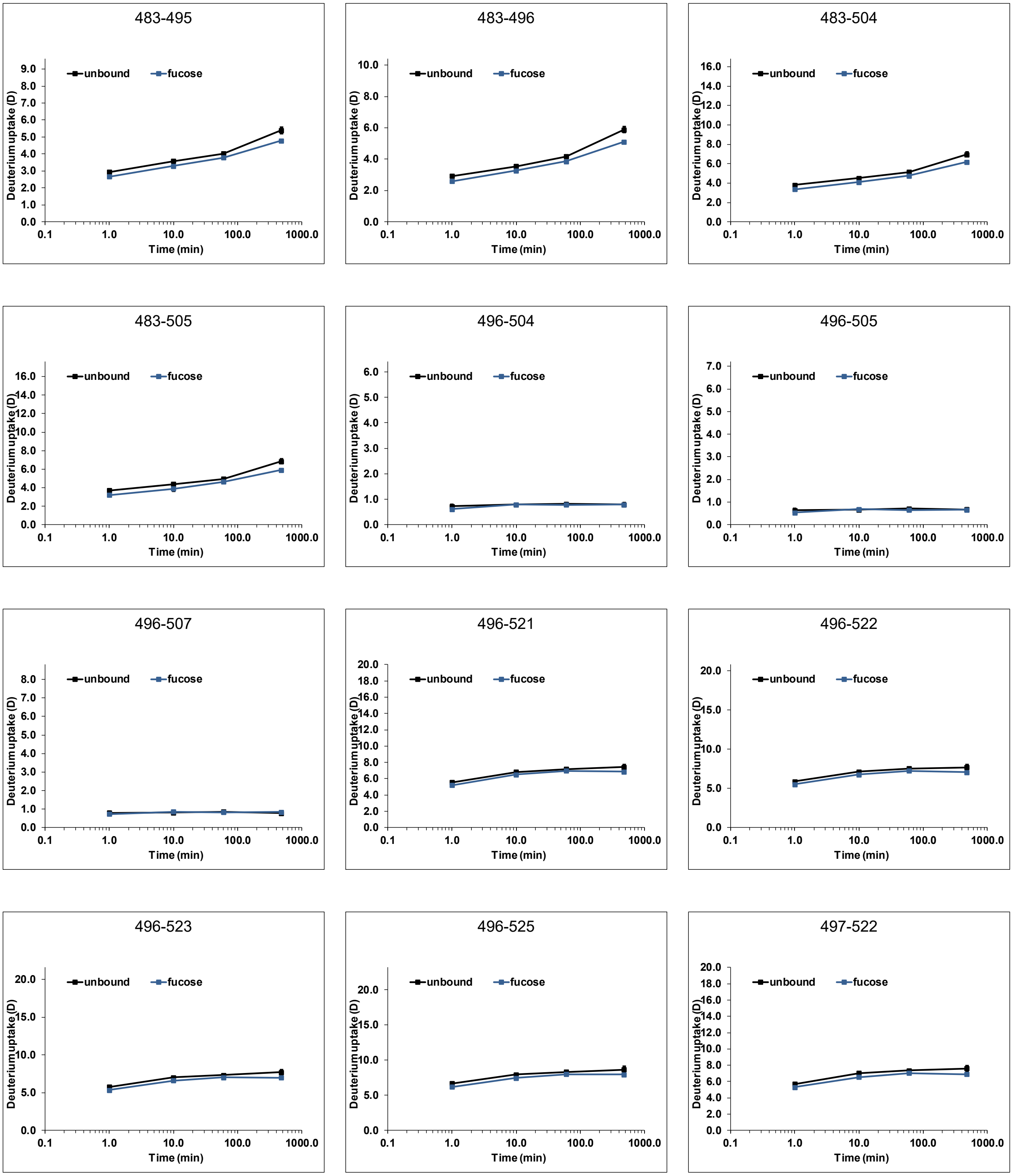

Figure S 26: continued

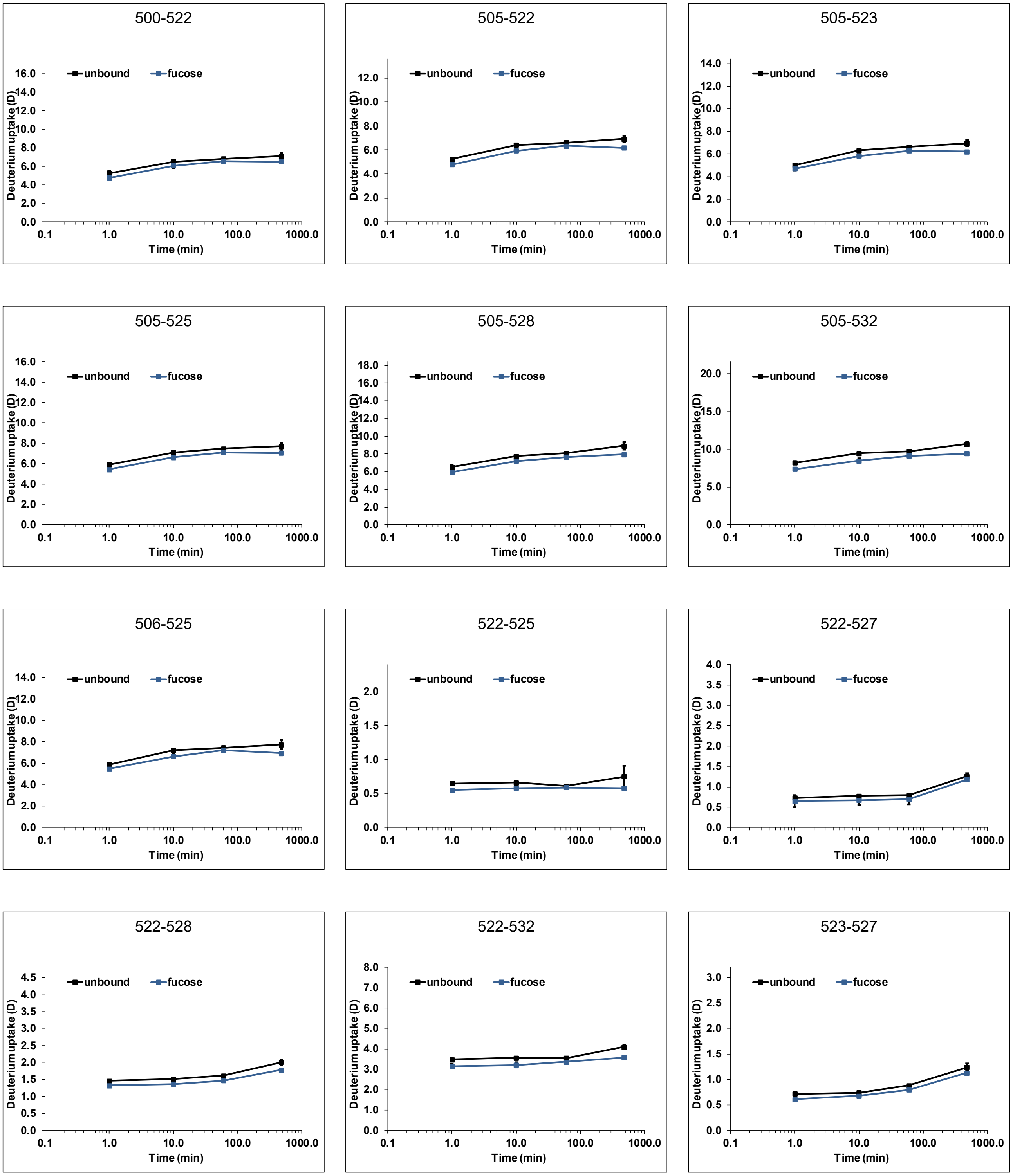

Figure S 26: continued

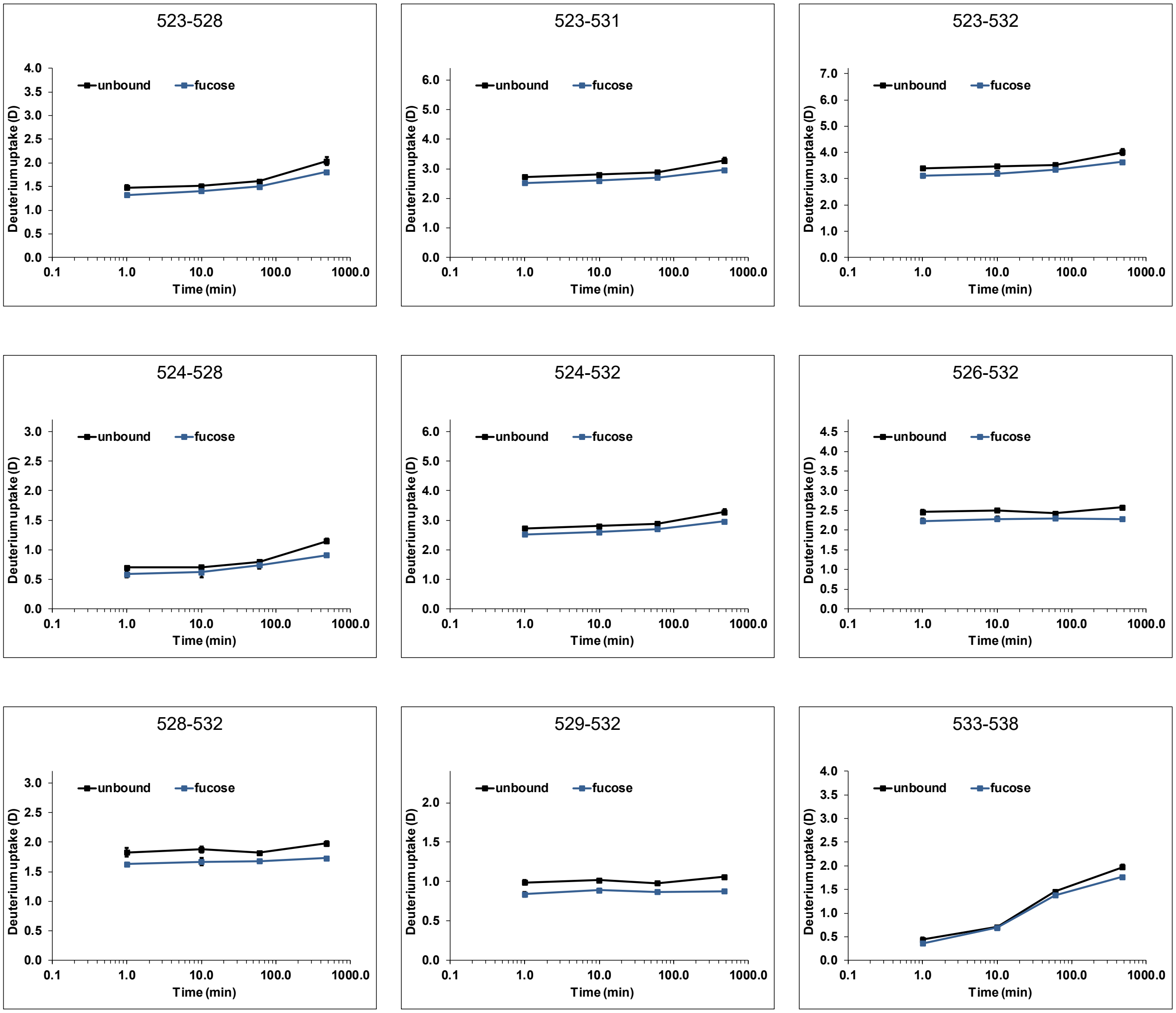

Figure S 26: continued

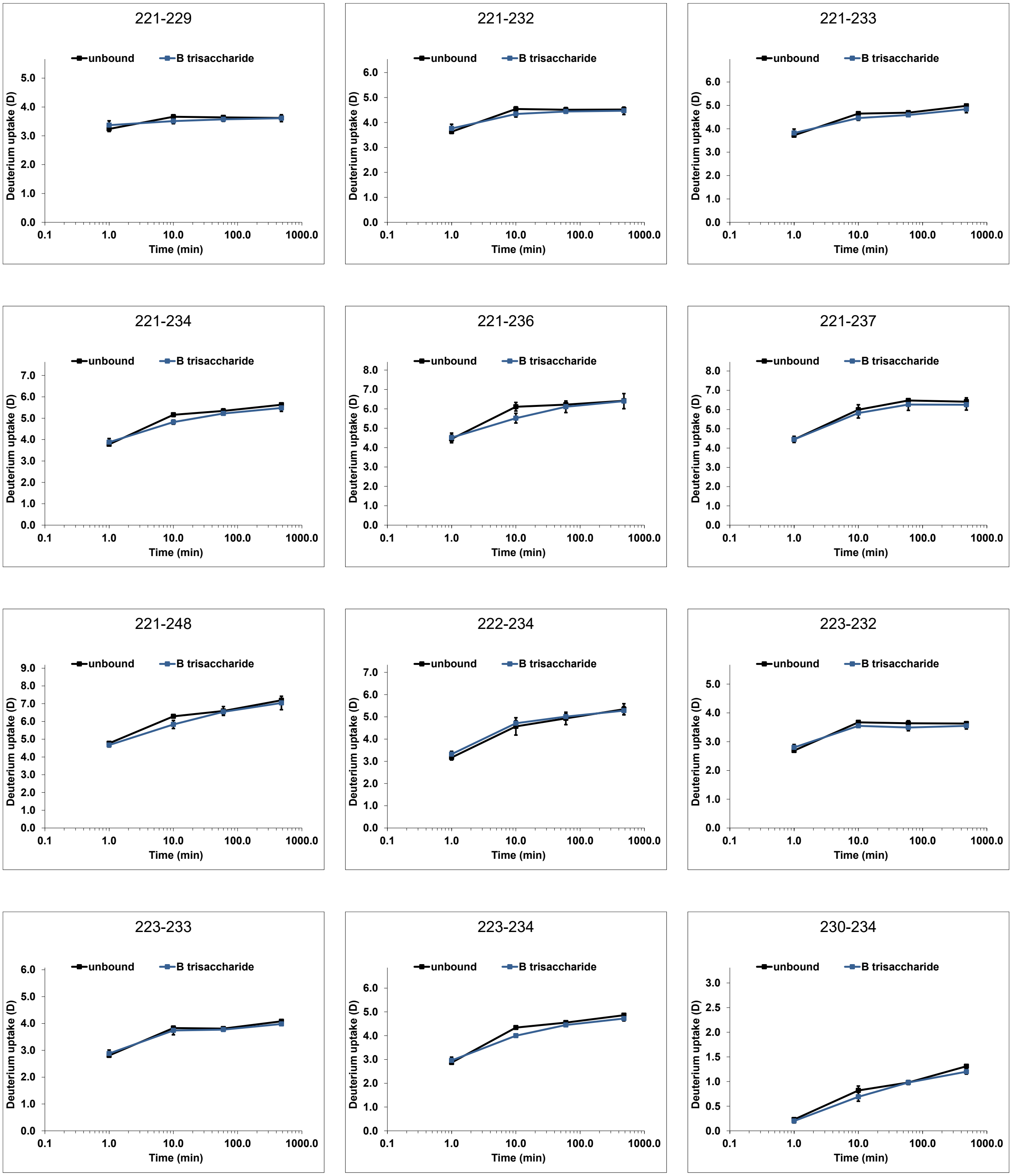

Figure S 27: Centroid deuterium uptake plots for Vietnam P dimers (black) with 10 mM HBGA B trisaccharide (blue) (dataset T2). All time points (1 min, 10 min, 1 h and 8 h) were performed in triplicate. Error bars indicate the standard deviation (SD). Deuteration values can be falsely low in centroid analysis of bimodal spectra. Compare Figure S 13 and Table S 3 for assessment of statistically significant deuteration differences.

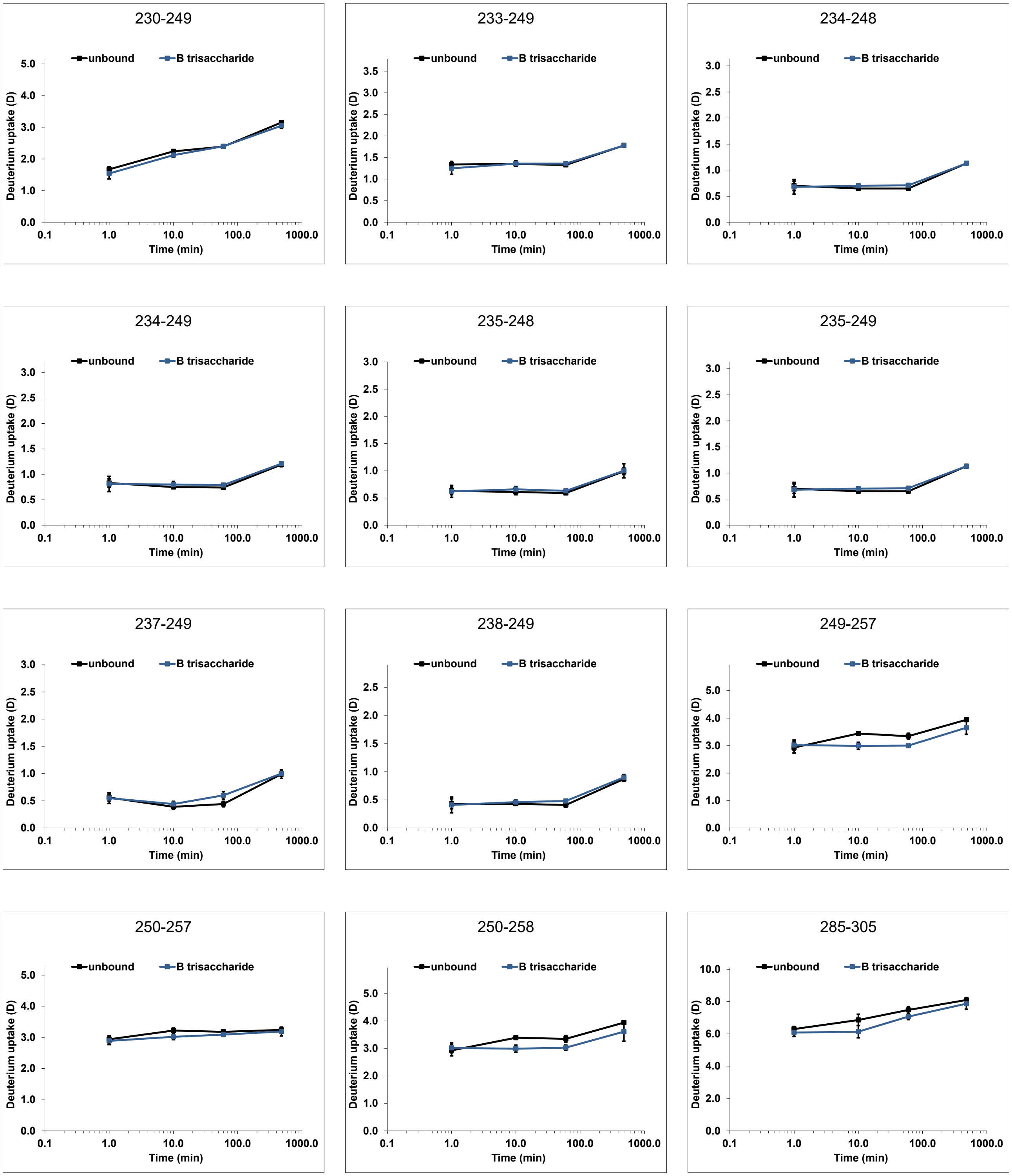

Figure S 27: continued

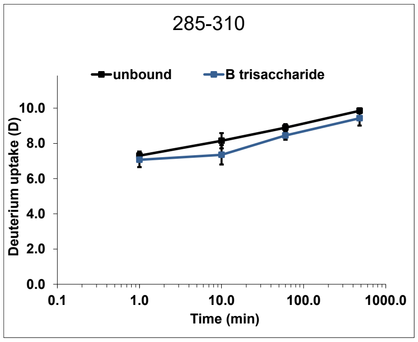

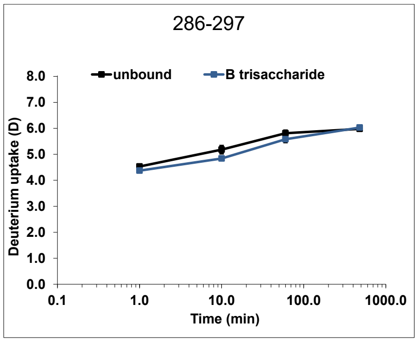

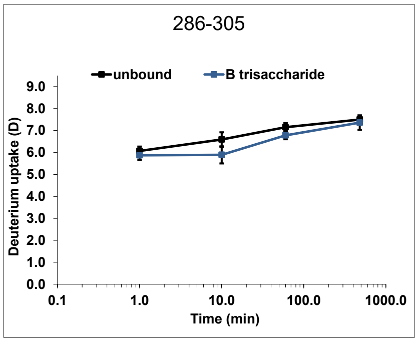

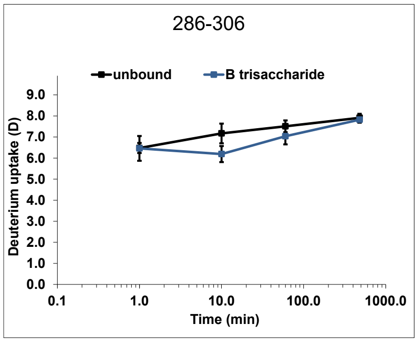

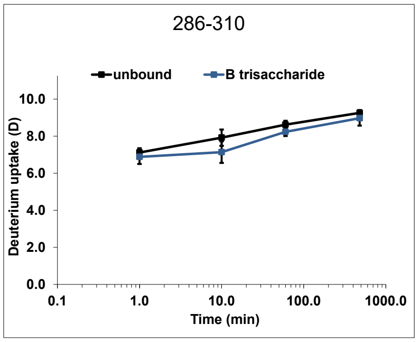

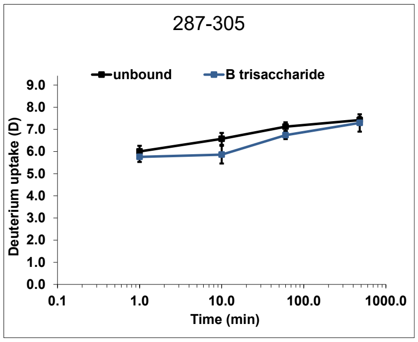

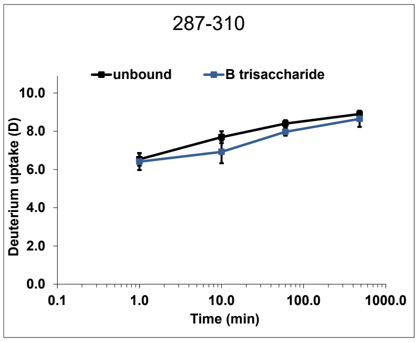

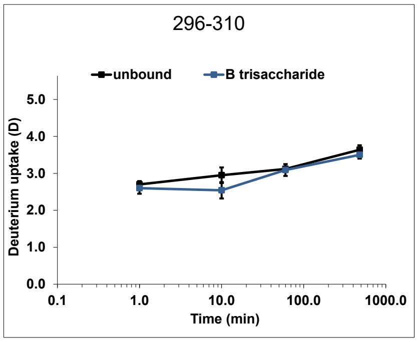

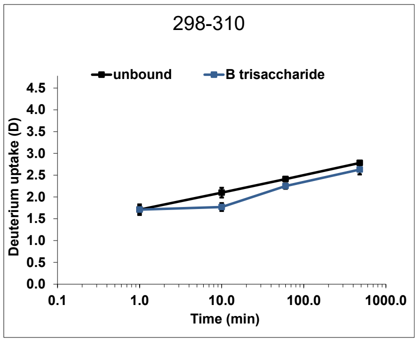

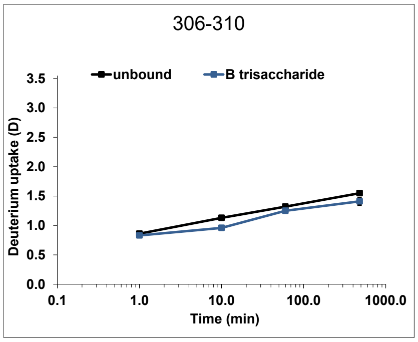

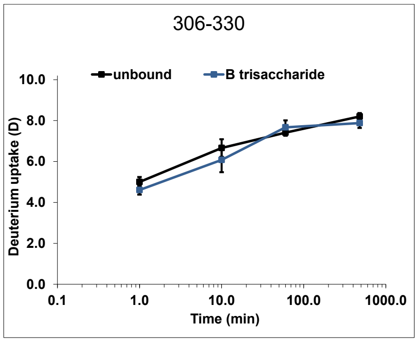

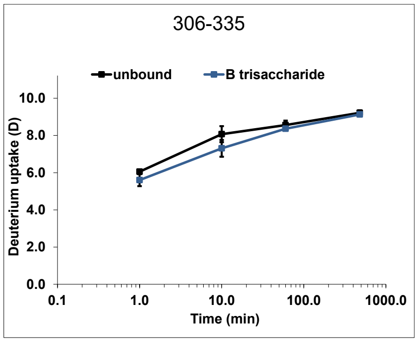

Figure S 27: continued

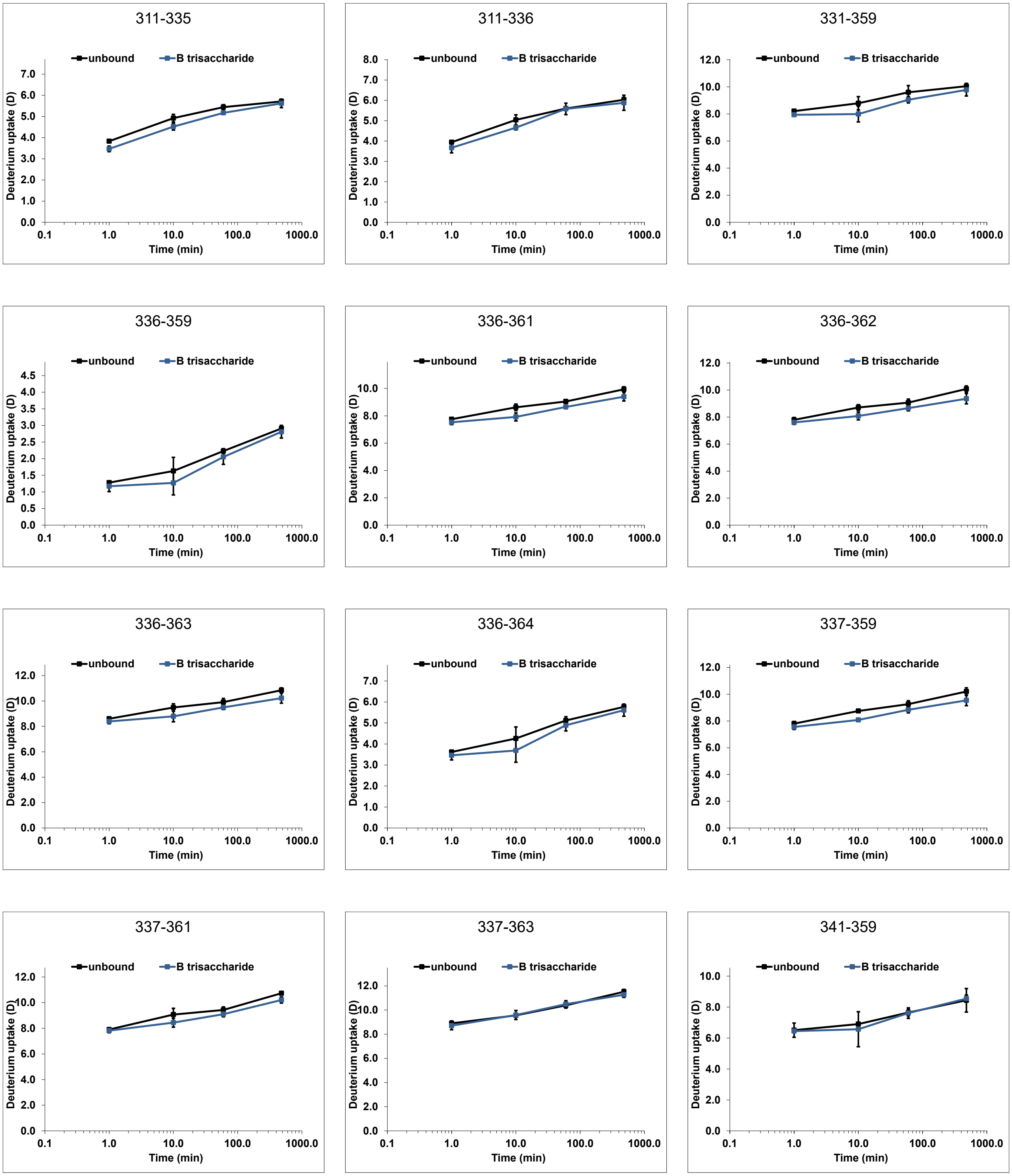

Figure S 27: continued

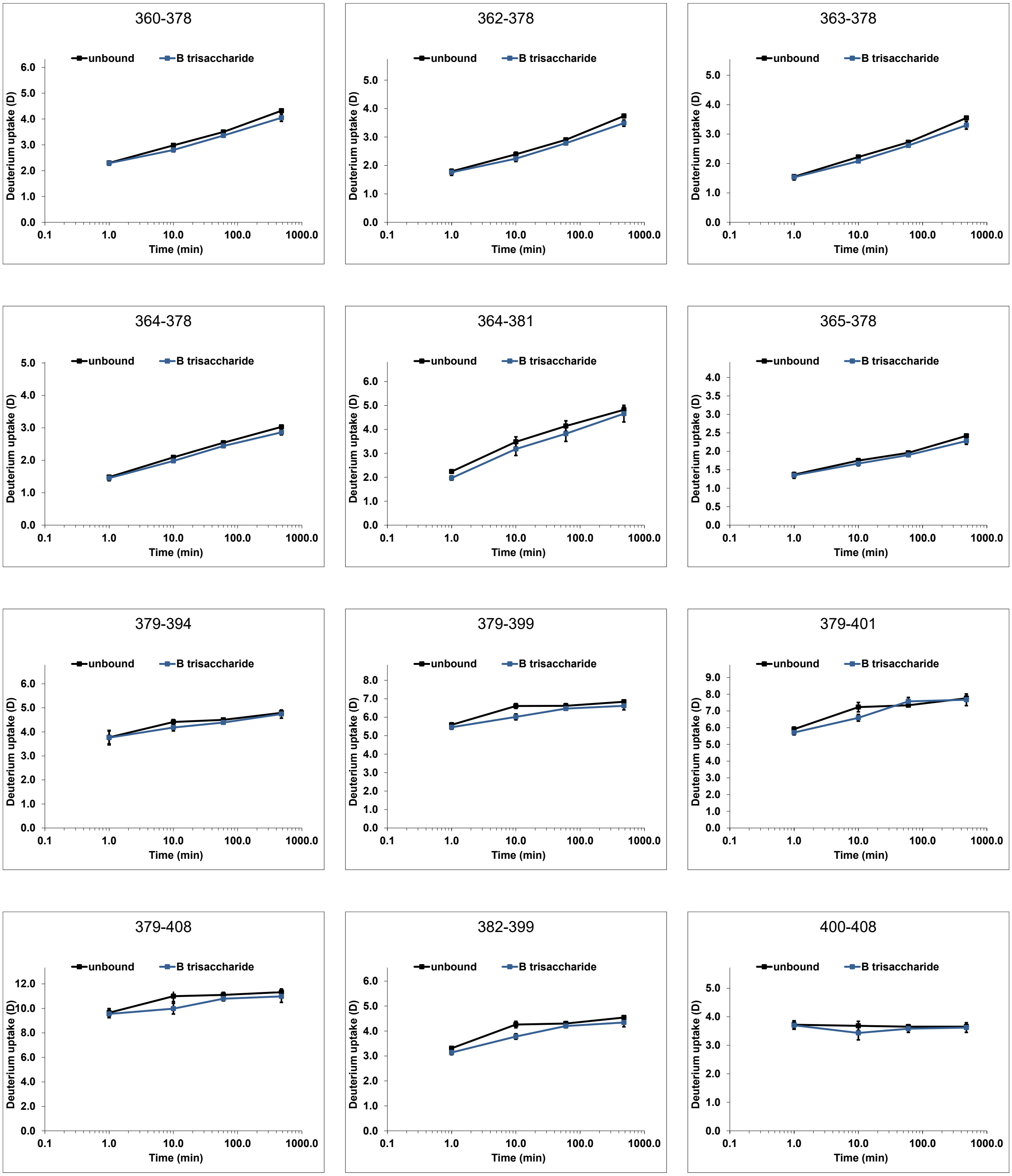

Figure S 27: continued

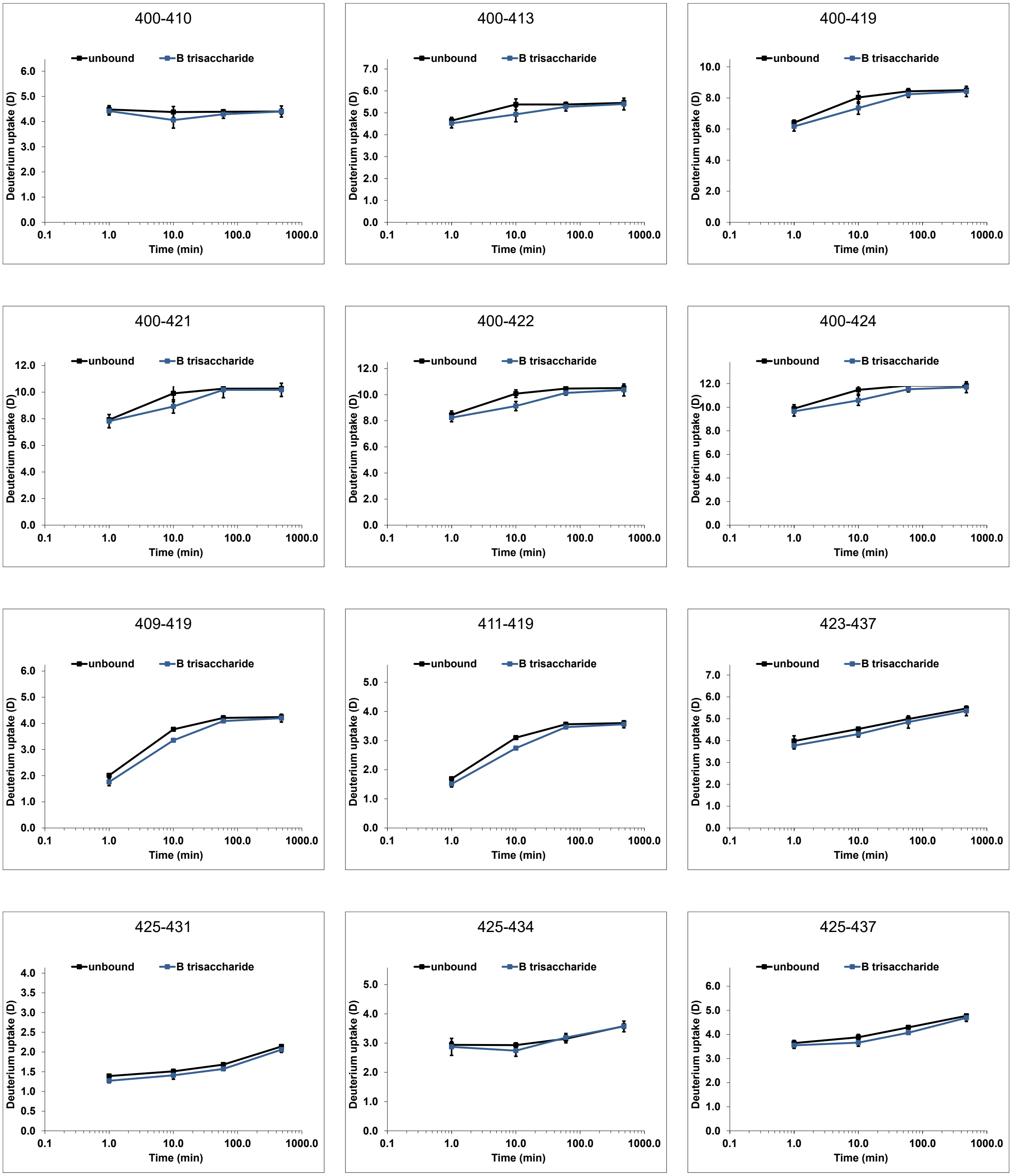

Figure S 27: continued

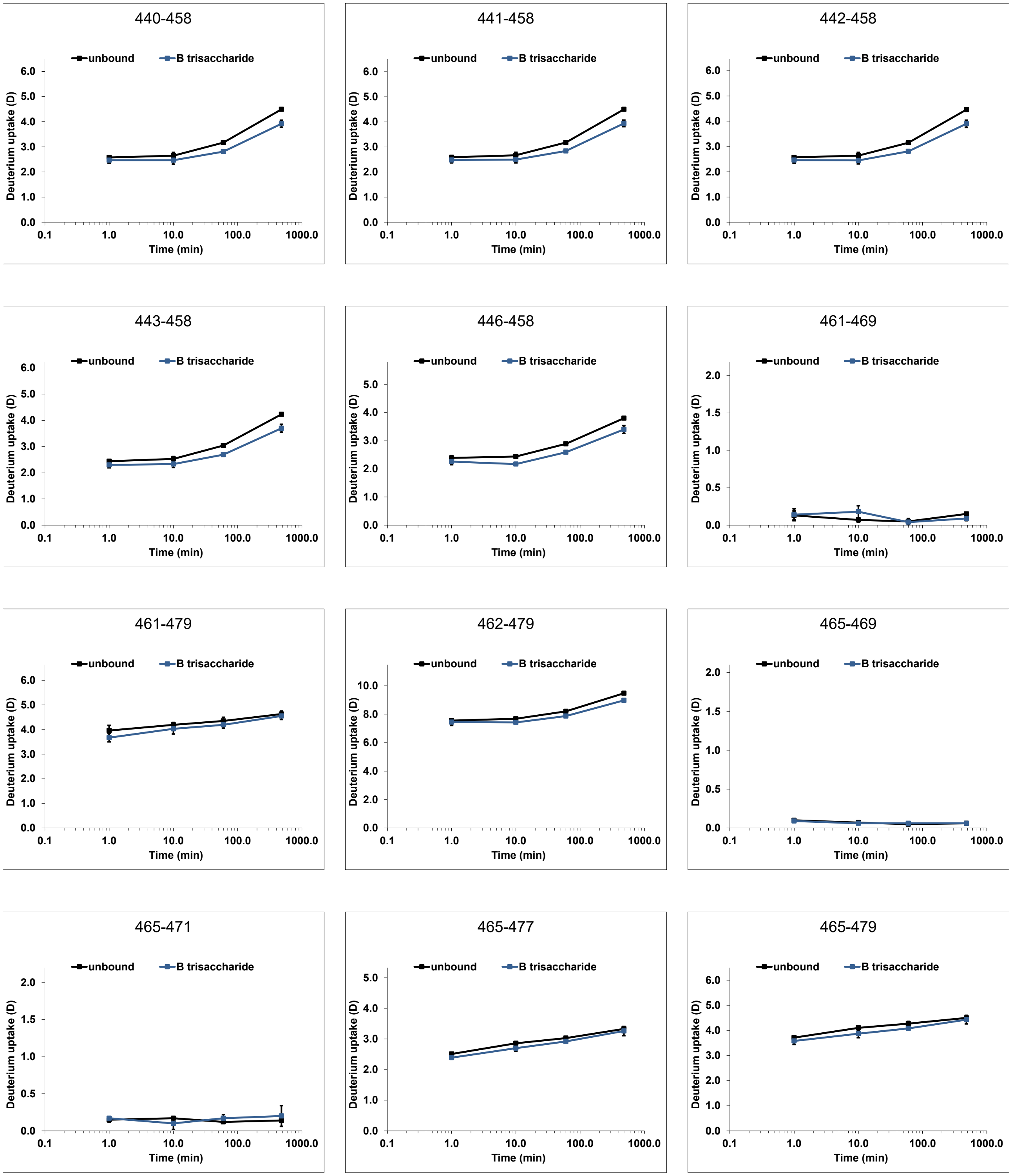

Figure S 27: continued

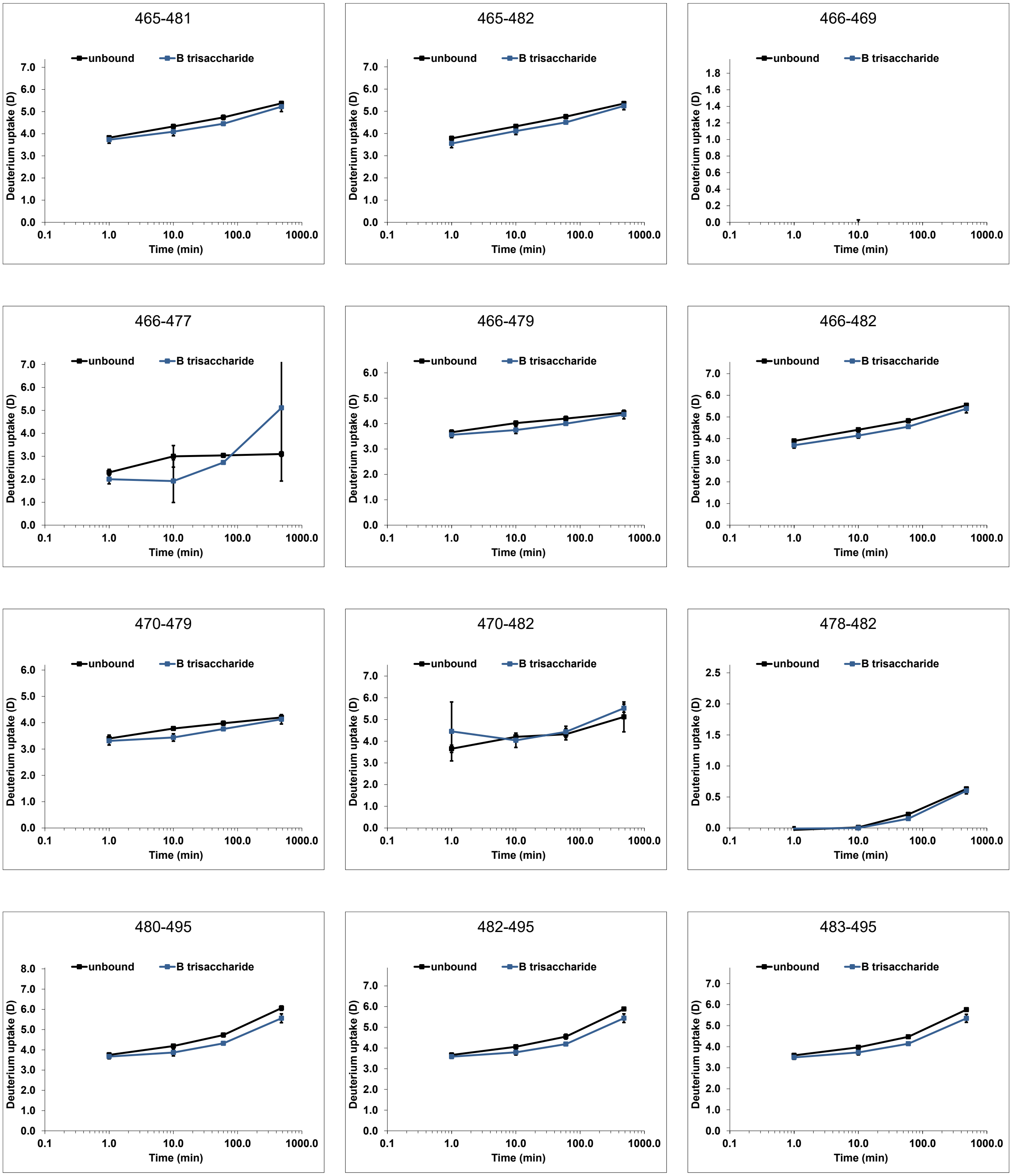

Figure S 27: continued

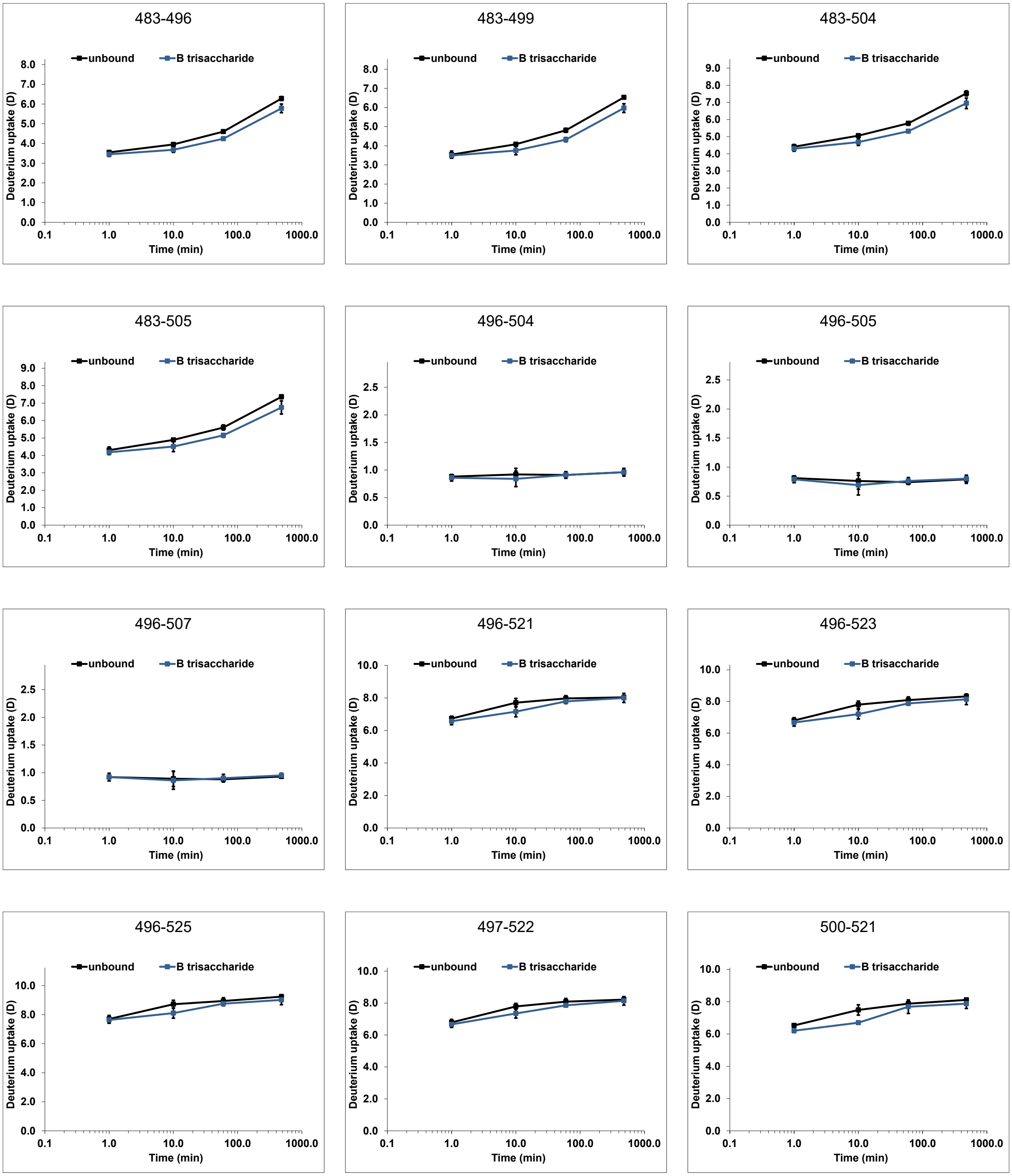

Figure S 27: continued

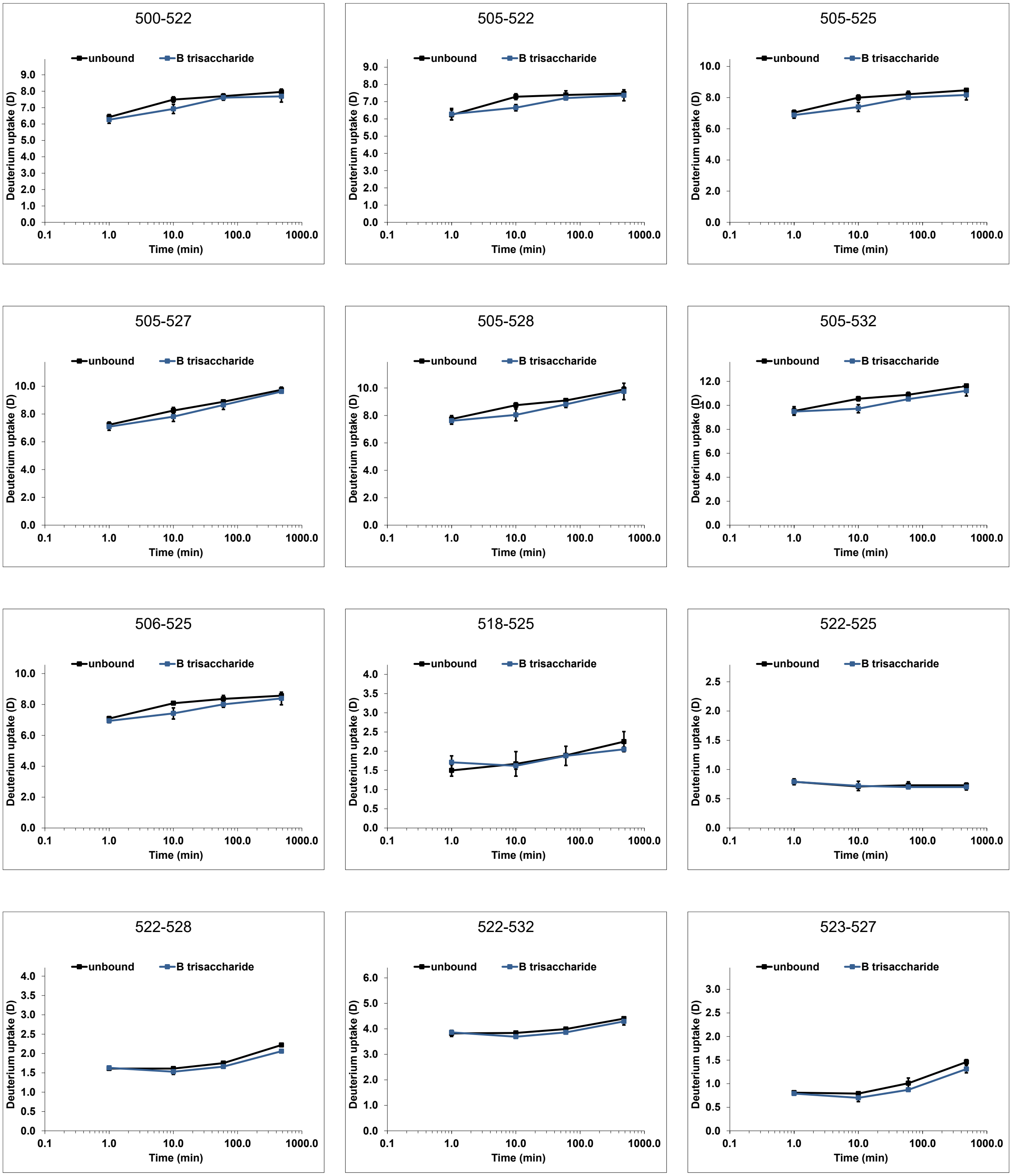

Figure S 27: continued

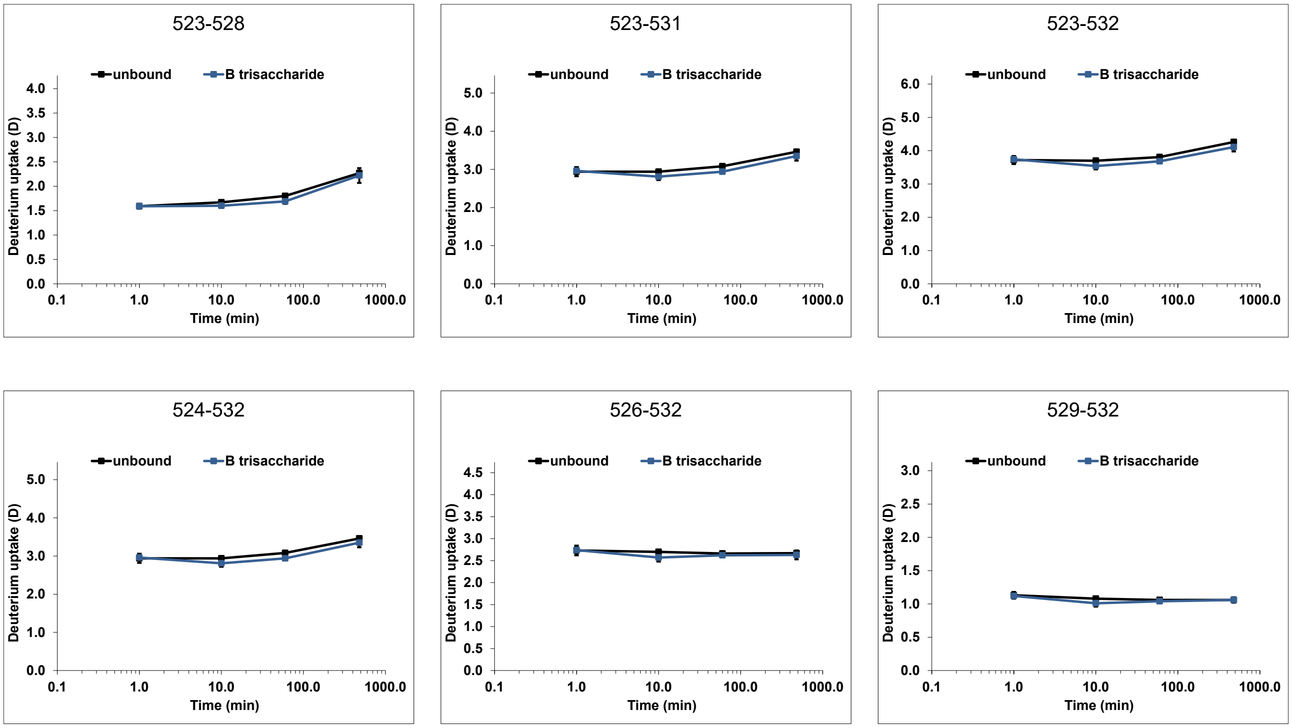

Figure S 27: continued

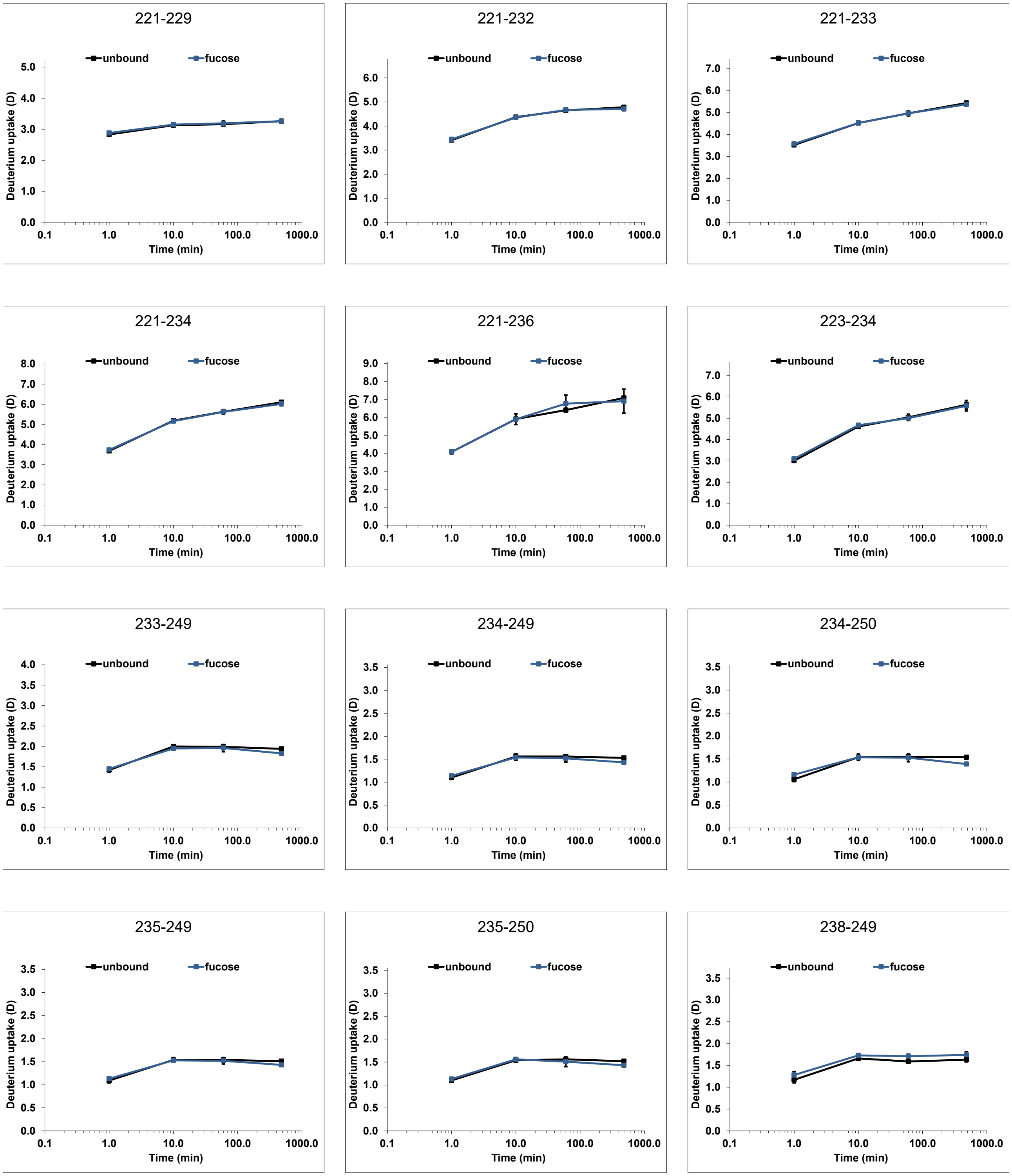

Figure S 28: Centroid deuterium uptake plots for Kawasaki P dimers (black) with 100 mM fucose (blue) (dataset T3). All time points (1 min, 10 min, 1 h and 8 h) were performed in triplicate. Error bars indicate the standard deviation (SD). Deuteration values can be falsely low in centroid analysis of bimodal spectra. Compare Figure S 13 and Table S 3 for assessment of statistically significant deuteration differences.

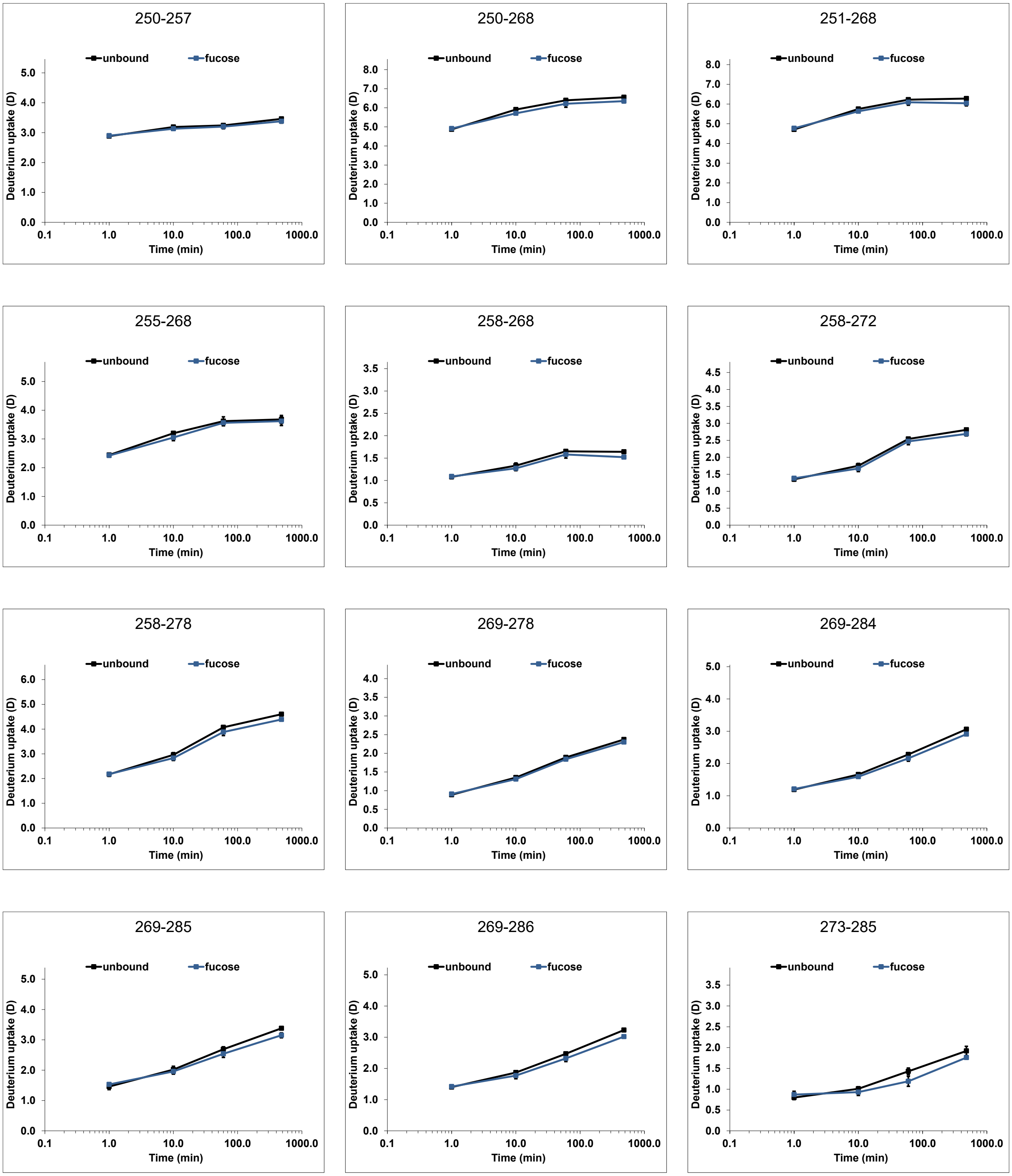

Figure S 28: continued

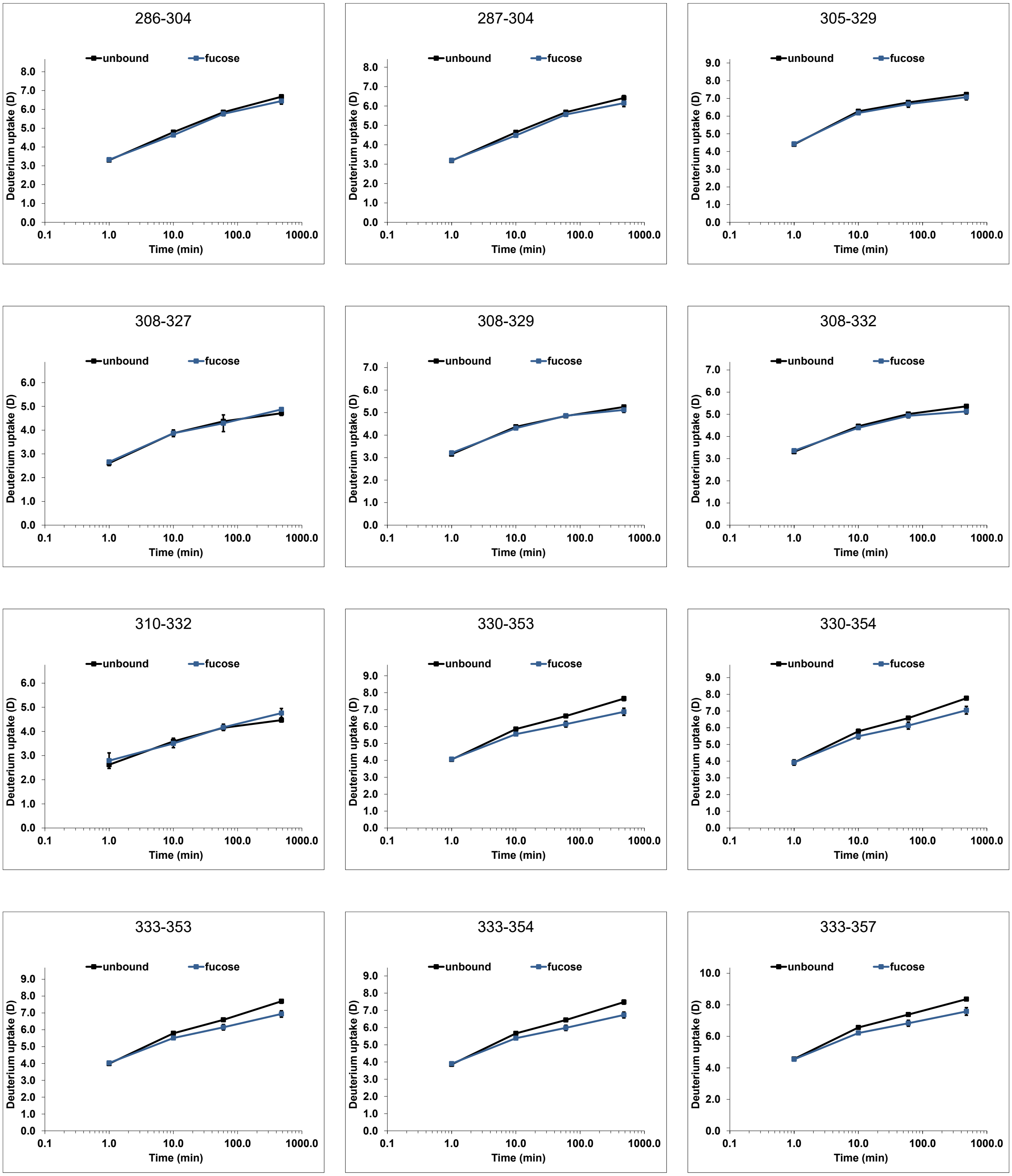

Figure S 28: continued

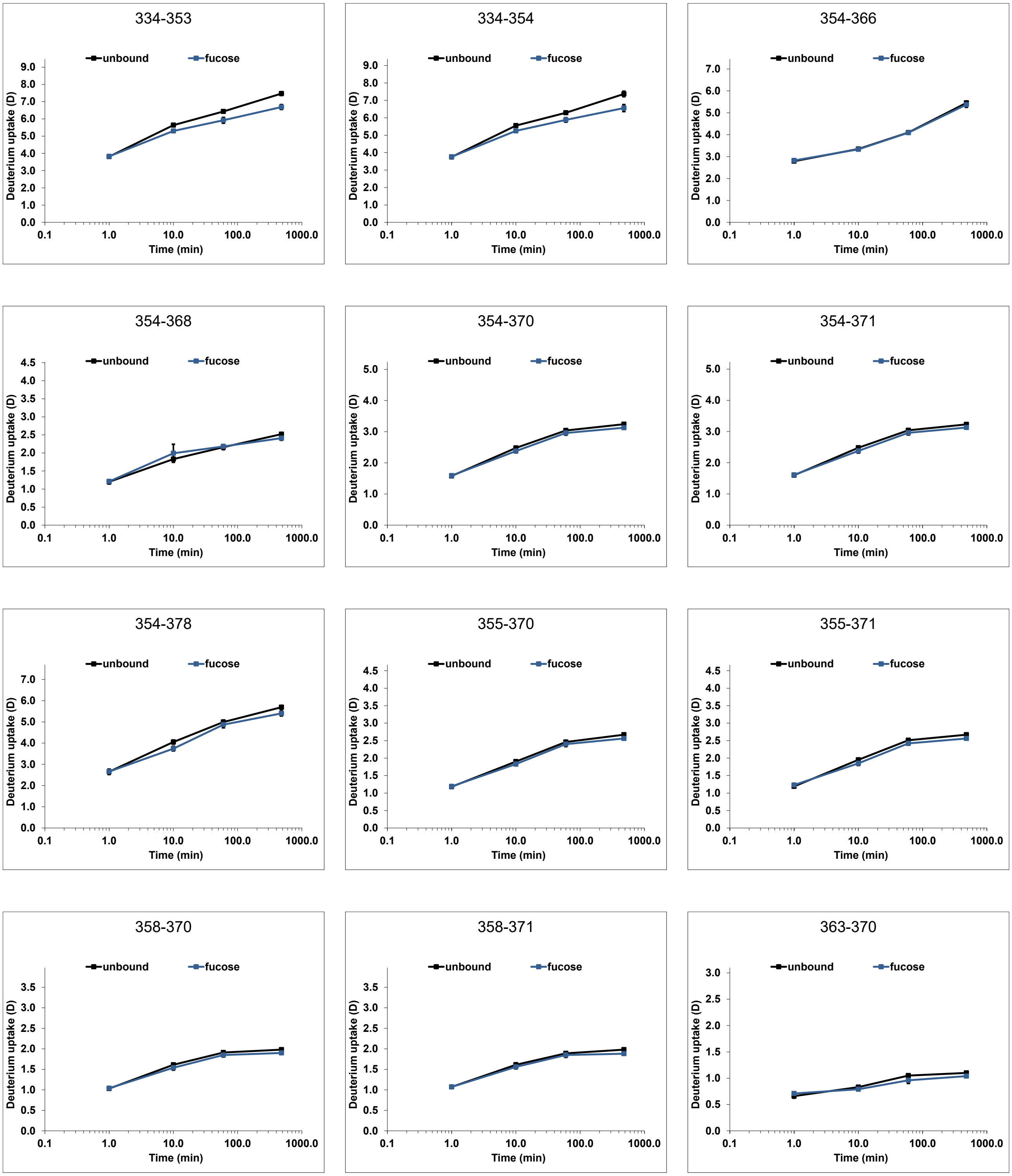

Figure S 28: continued

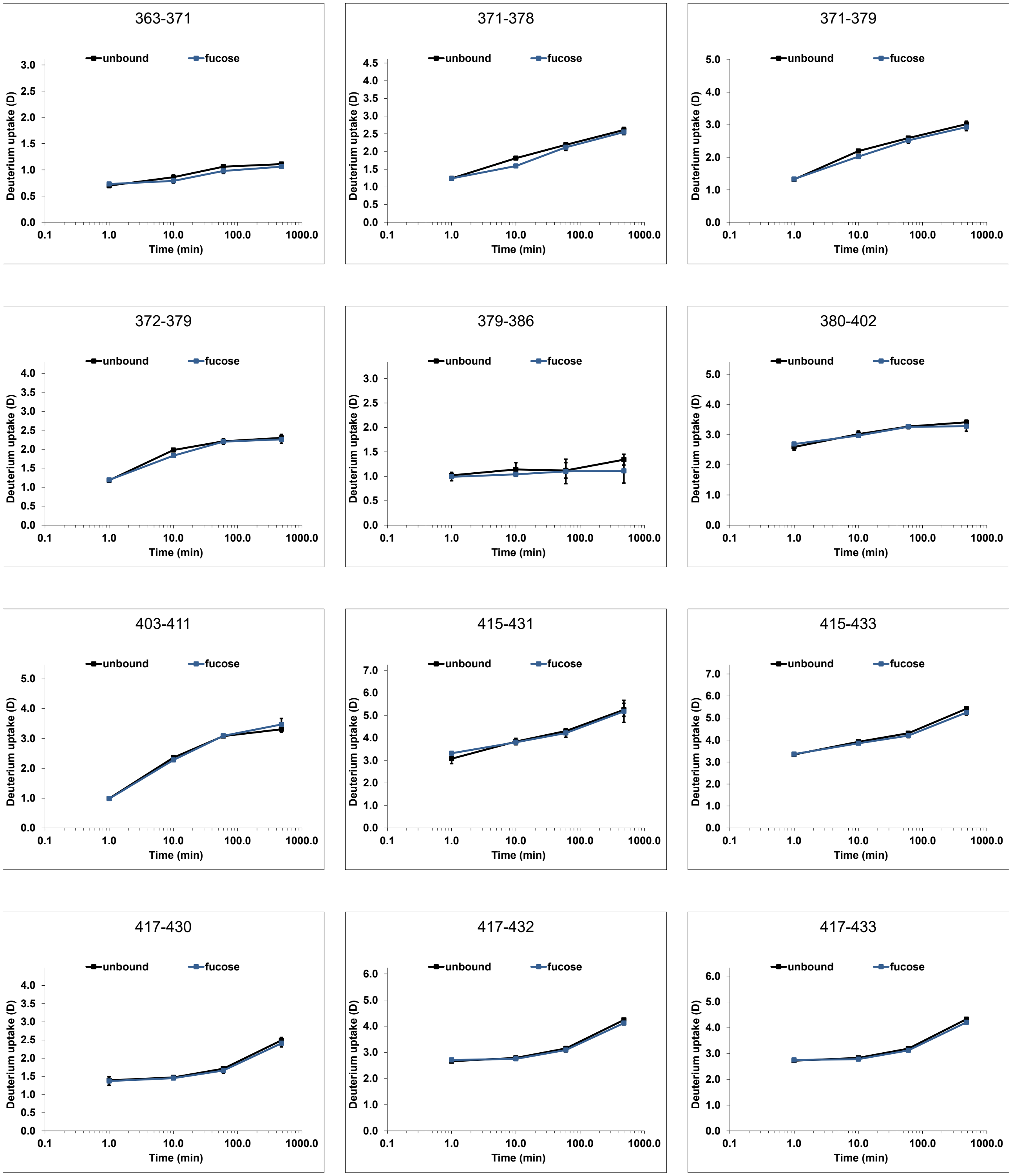

Figure S 28: continued

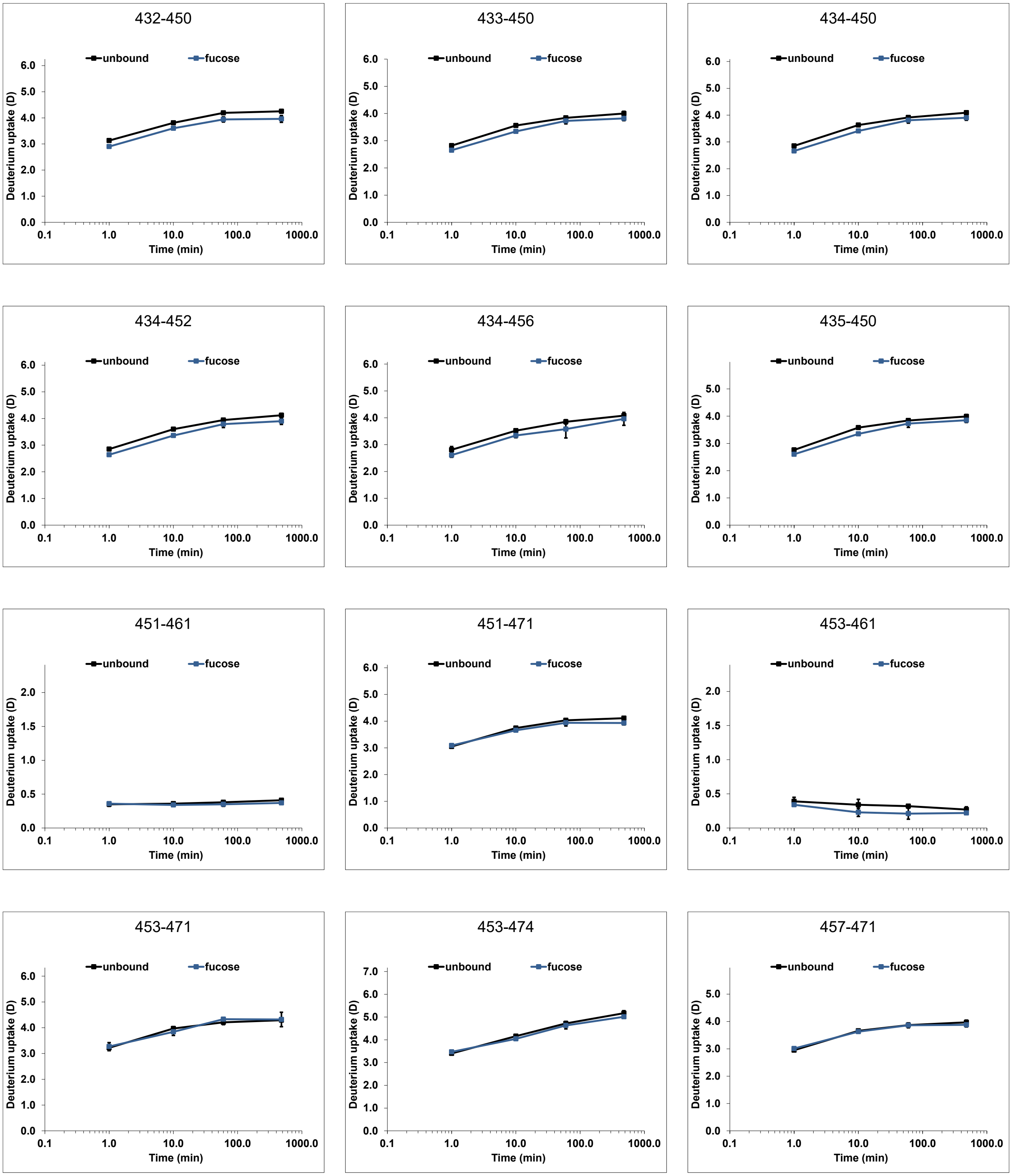

Figure S 28: continued

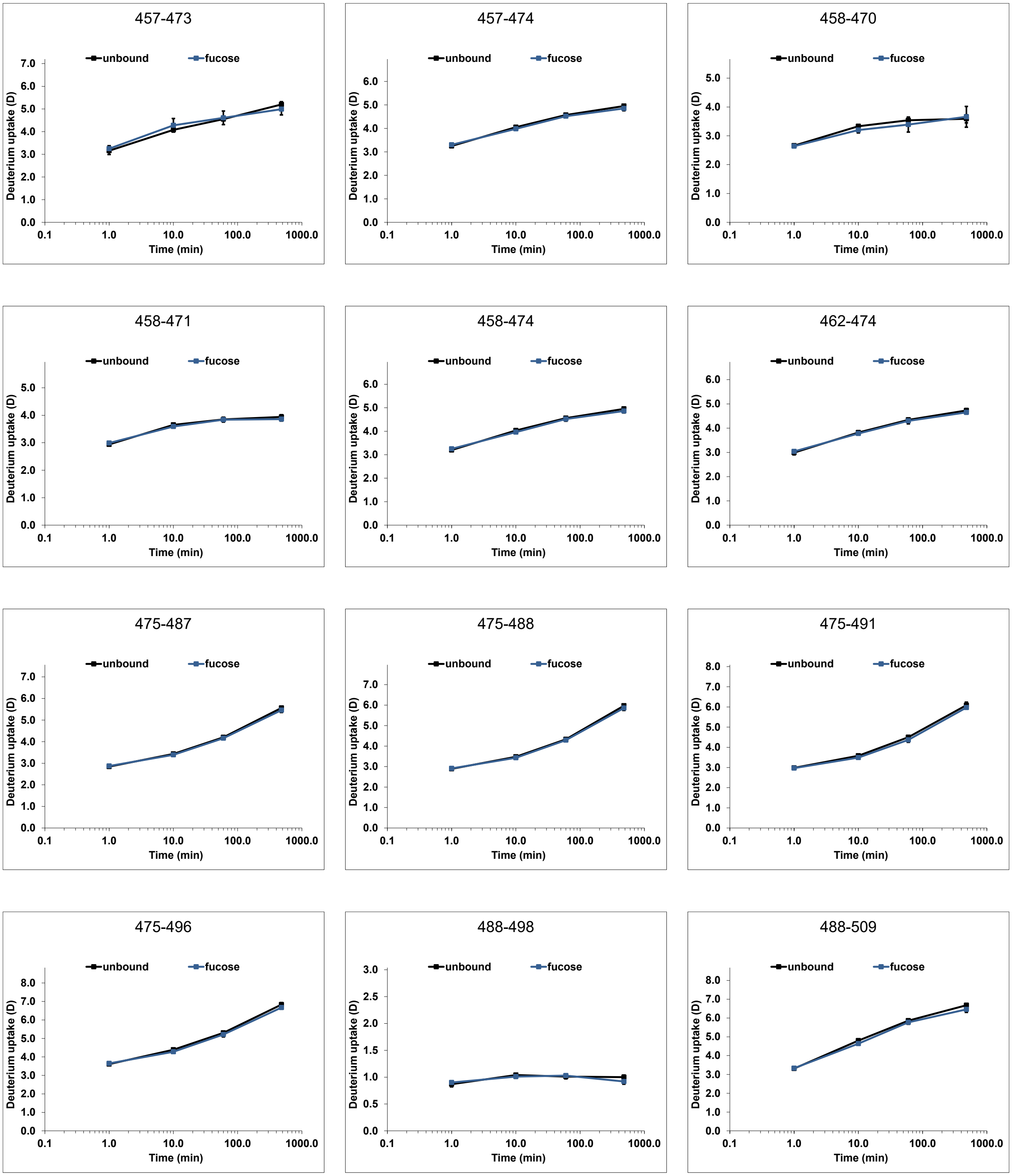

Figure S 28: continued

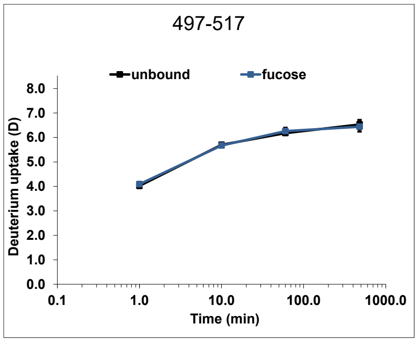

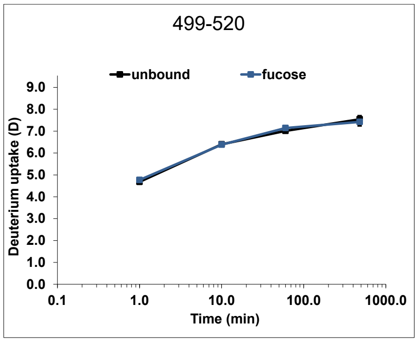

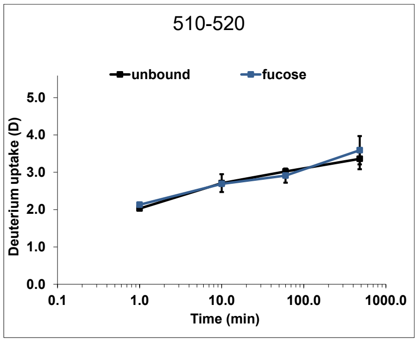

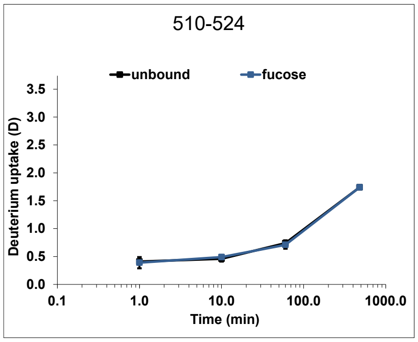

Figure S 28: continued

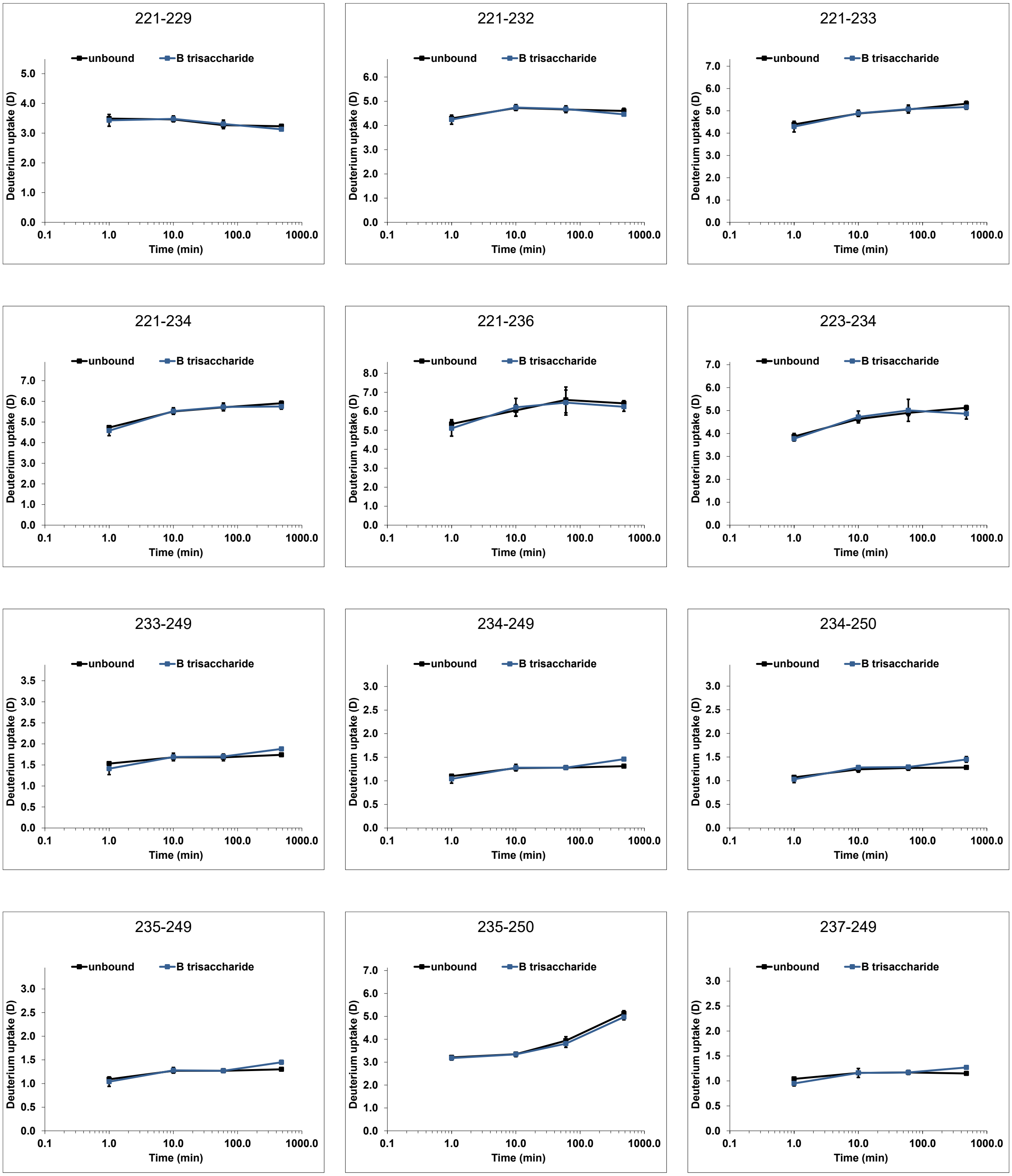

Figure S 29: Centroid deuterium uptake plots for Kawasaki P dimers (black) with 10 mM HBGA B trisaccharide (blue) (dataset T4). All time points (1 min, 10 min, 1 h and 8 h) were performed in triplicate. Error bars indicate the standard deviation (SD). Deuteration values can be falsely low in centroid analysis of bimodal spectra. Compare Figure S 13 and Table S3 for assessment of statistically significant deuteration differences.

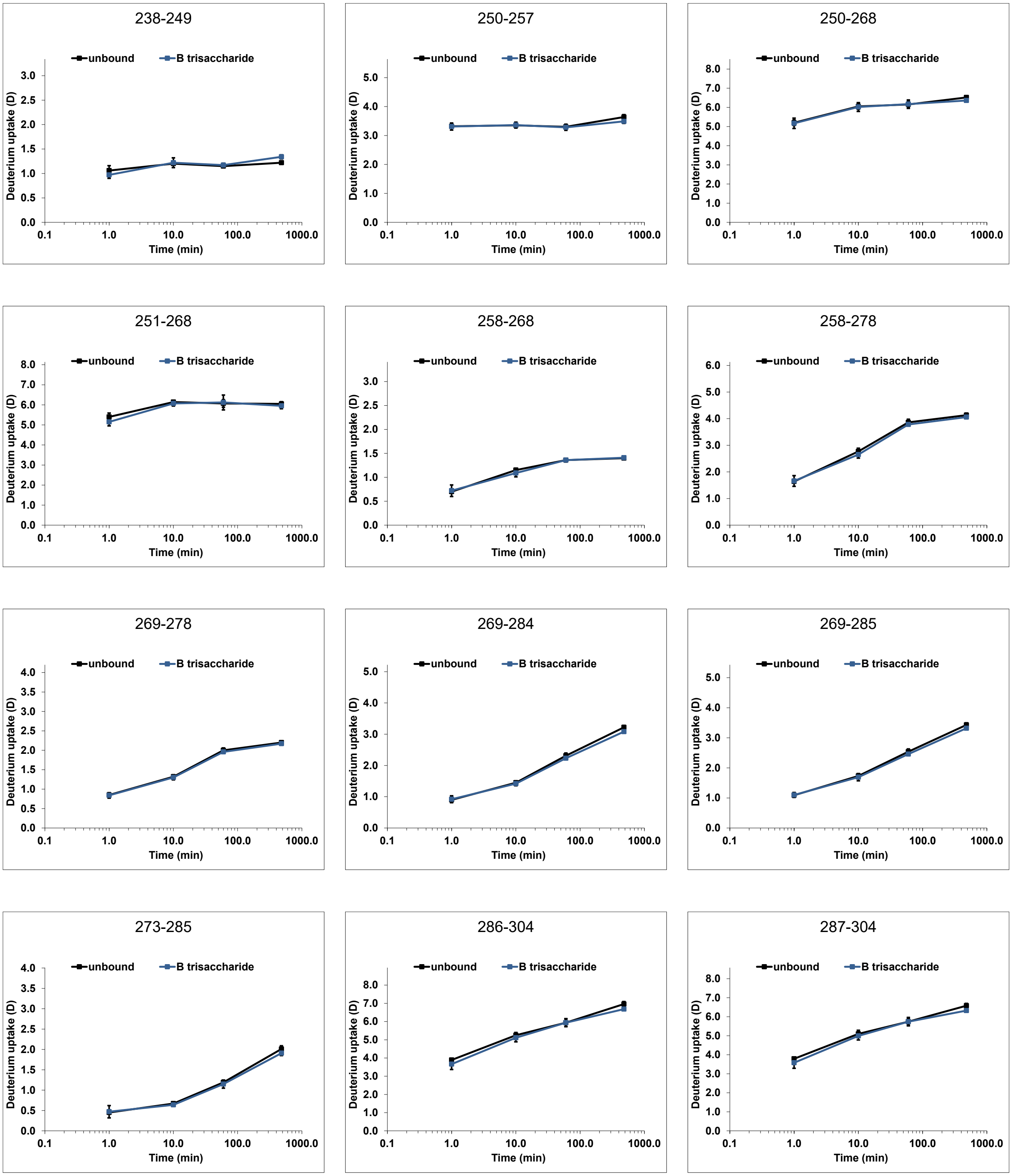

Figure S 29: continued

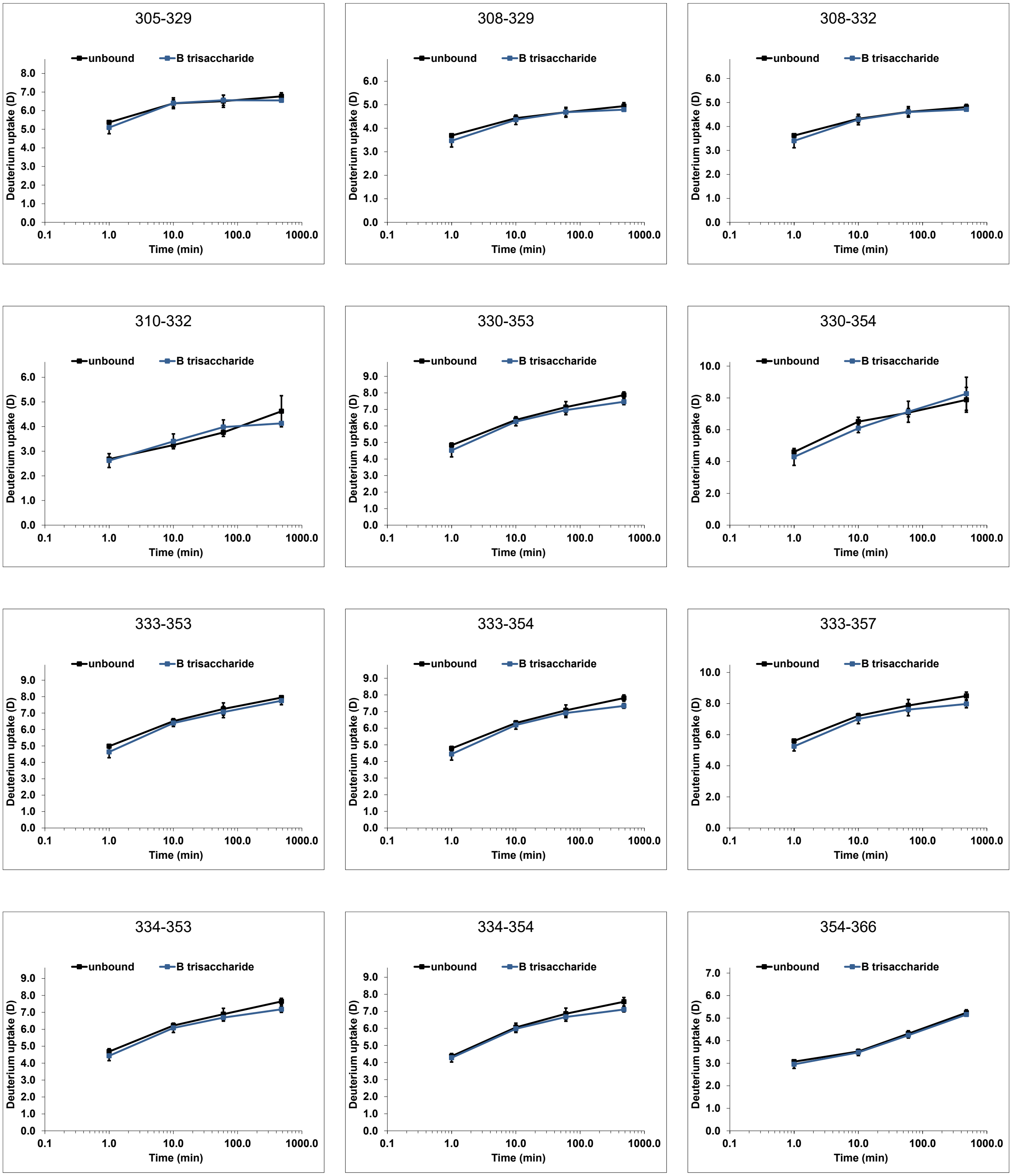

Figure S 29: continued

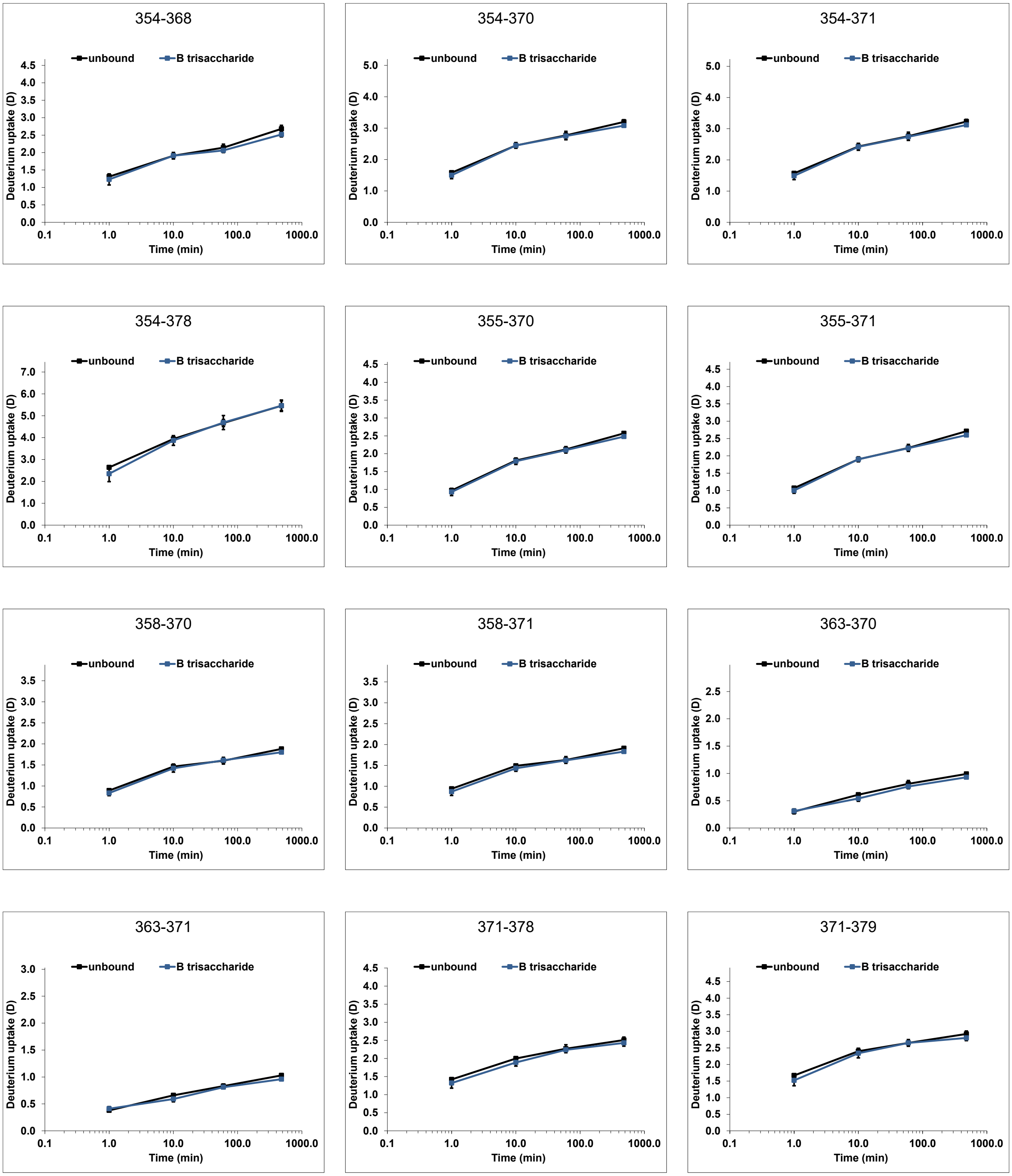

Figure S 29: continued

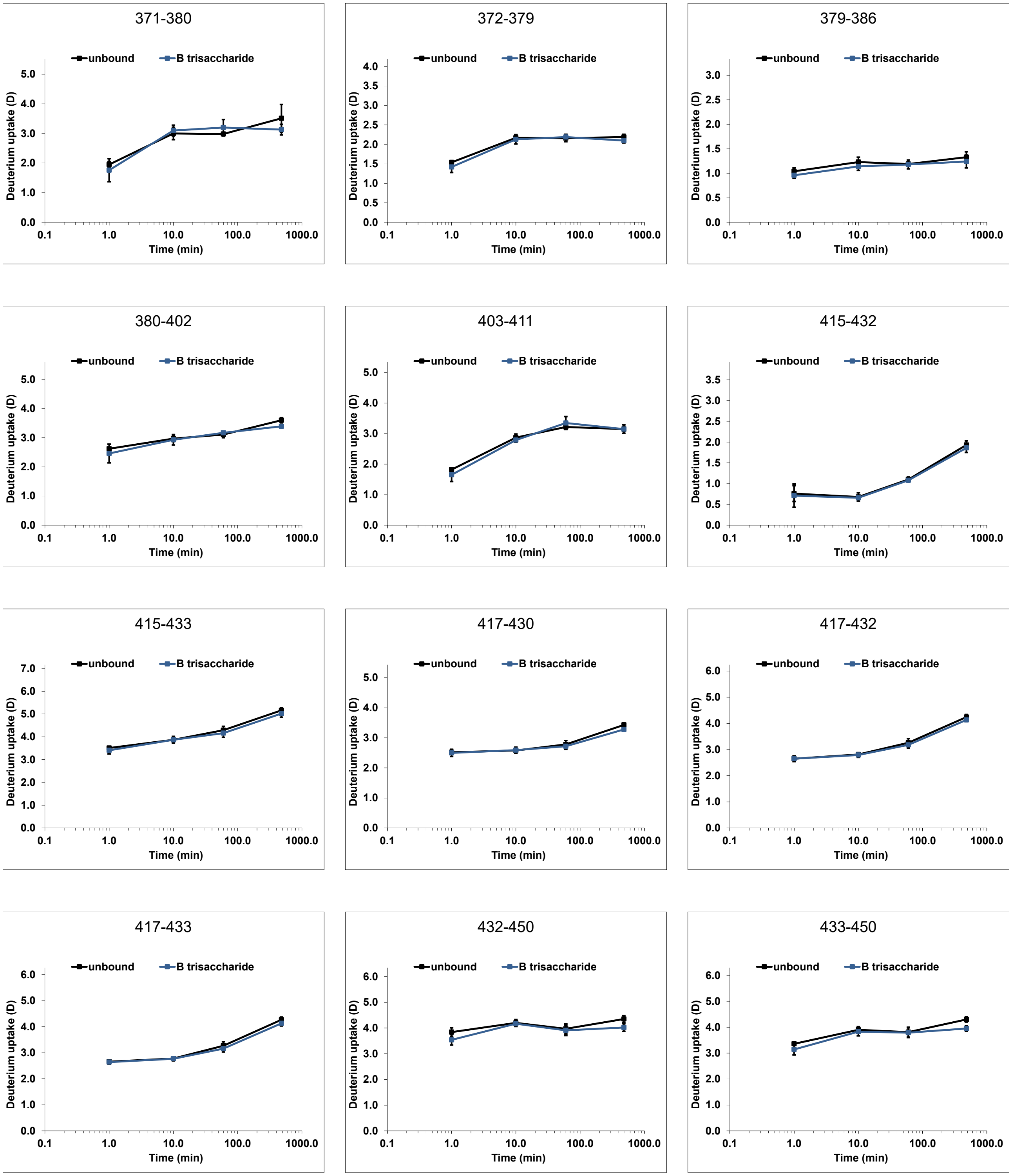

Figure S 29: continued

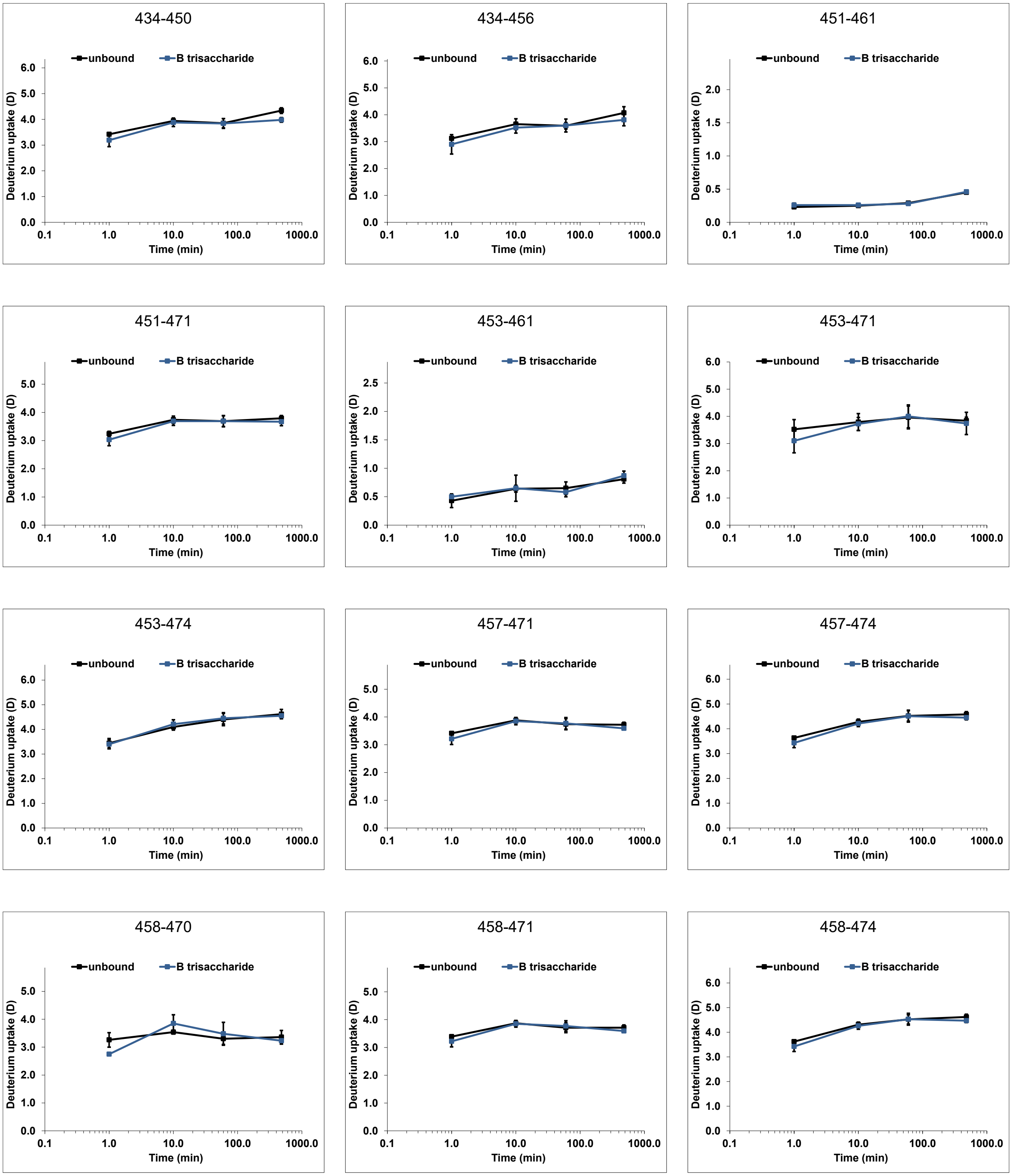

Figure S 29: continued

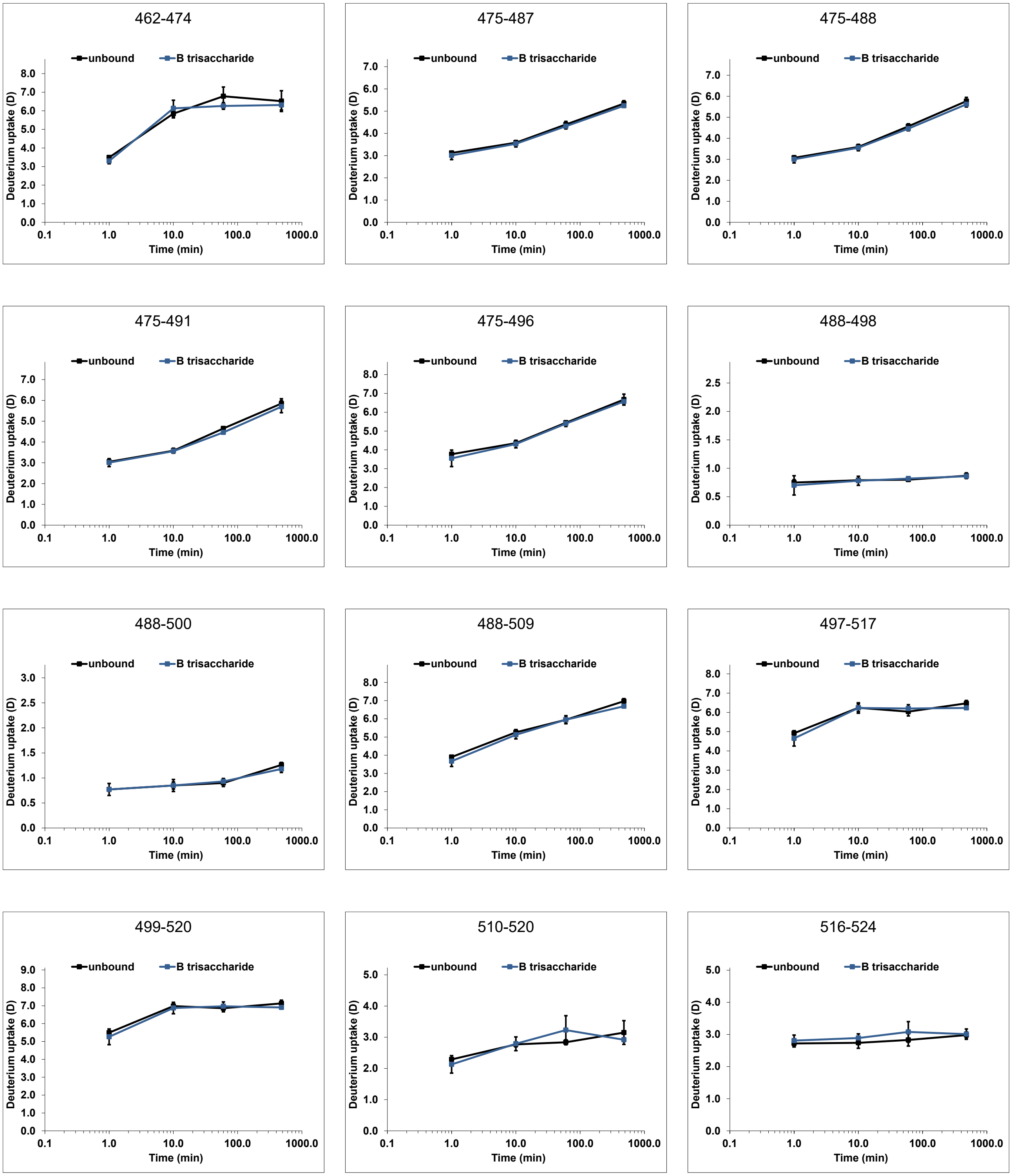

Figure S 29: continued

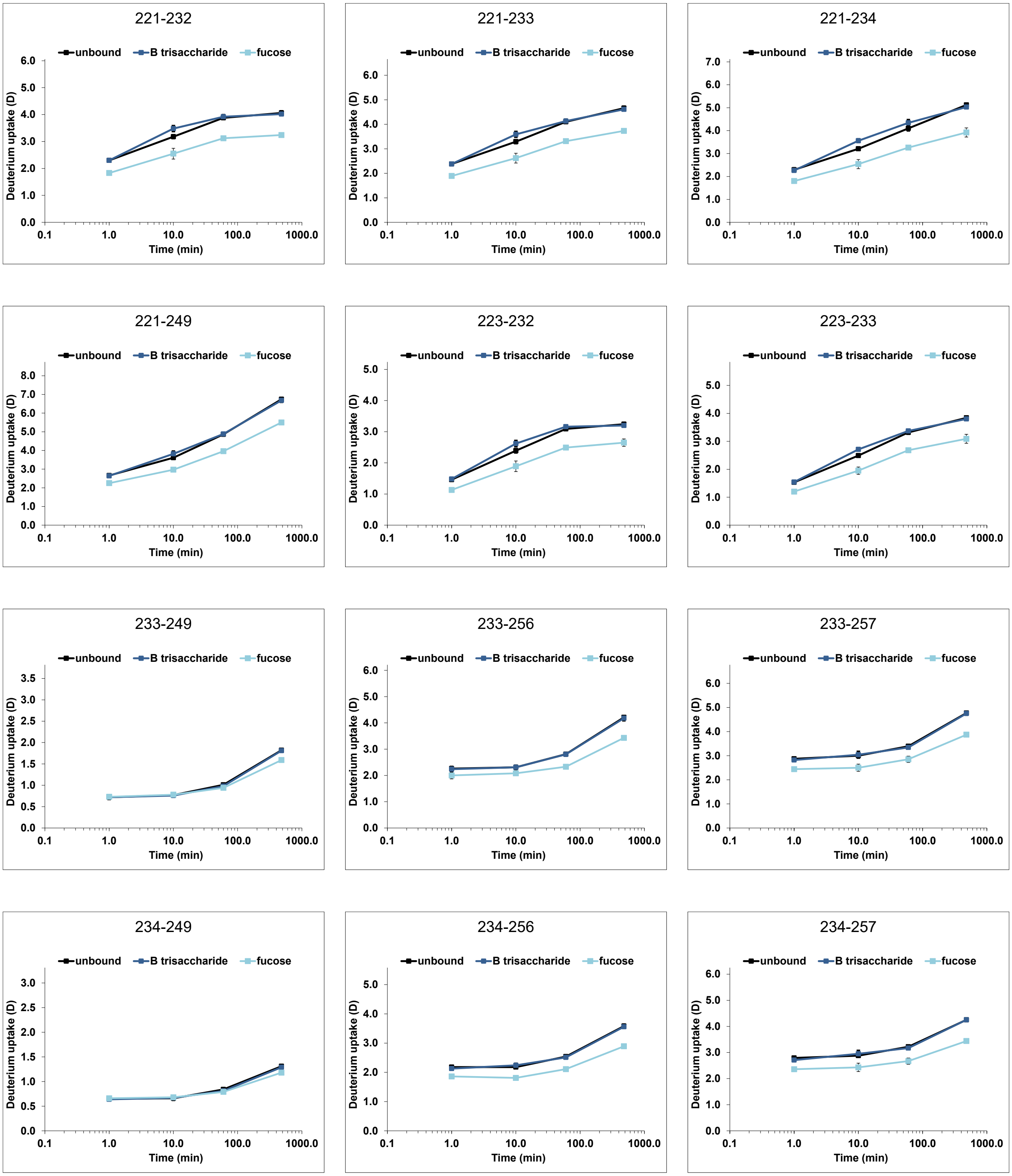

Figure S 30: Centroid deuterium uptake plots for wildtype MI001 P dimers (black) with 10 mM HBGA B trisaccharide (blue) and 100 mM fucose (light blue) (dataset T5). All time points (1 min, 10 min, 1 h and 8 h) were performed in triplicate. Error bars indicate the standard deviation (SD). Deuteration values can be falsely low in centroid analysis of bimodal spectra, especially in the fucose state. Compare Figure S 13 and Table S3 for assessment of statistically significant deuteration differences.

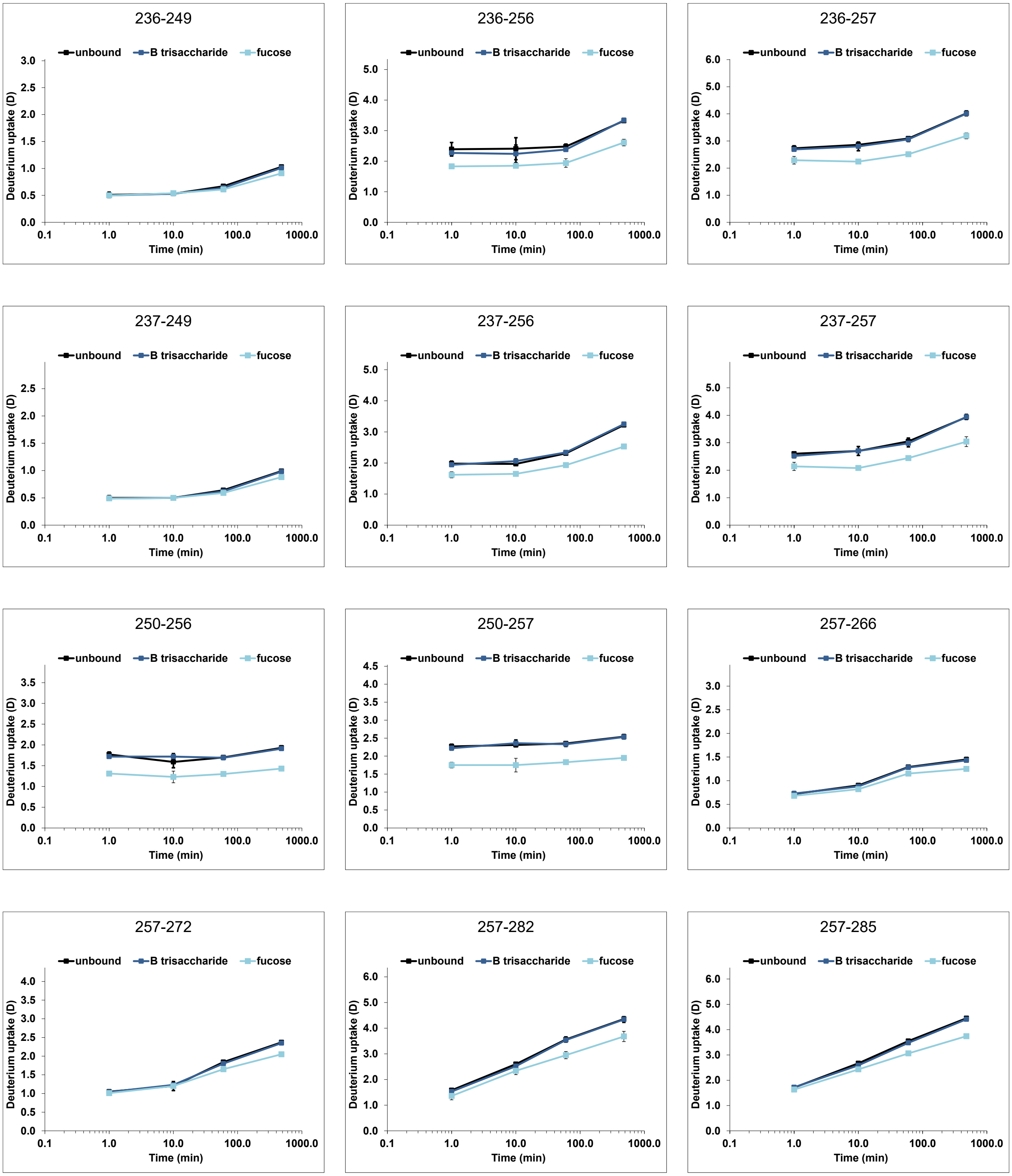

Figure S 30: continued

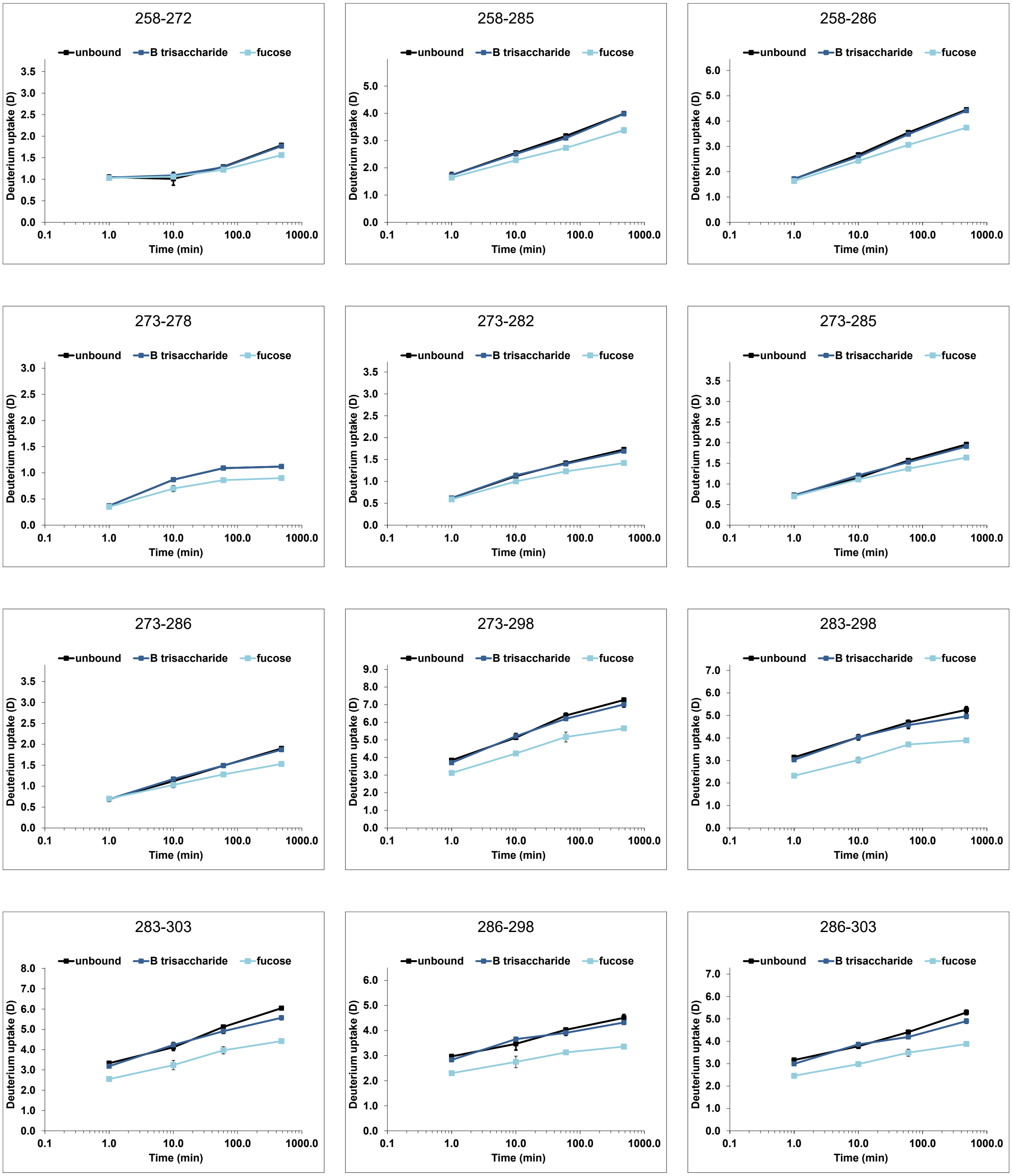

Figure S 30: continued

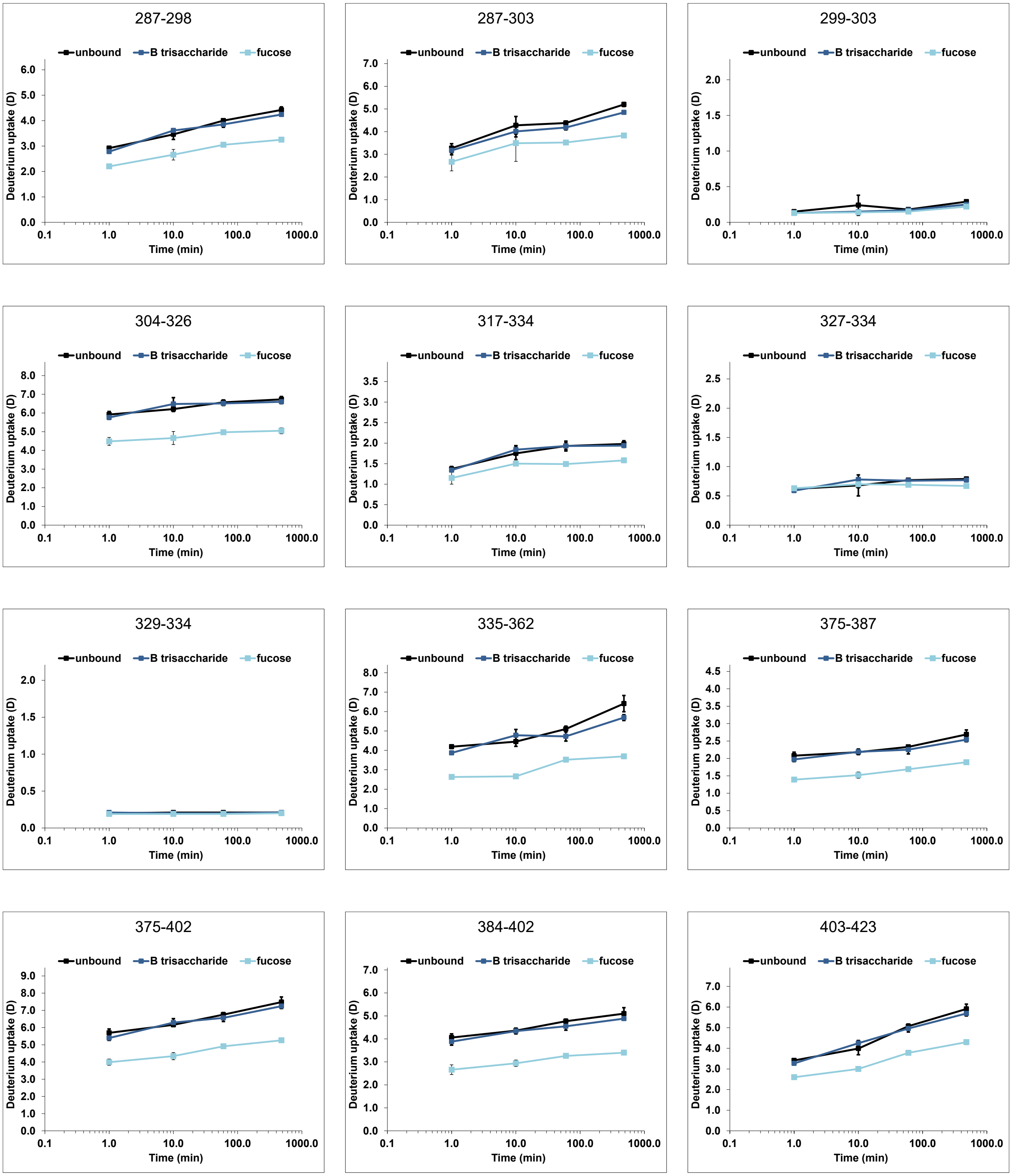

Figure S 30: continued

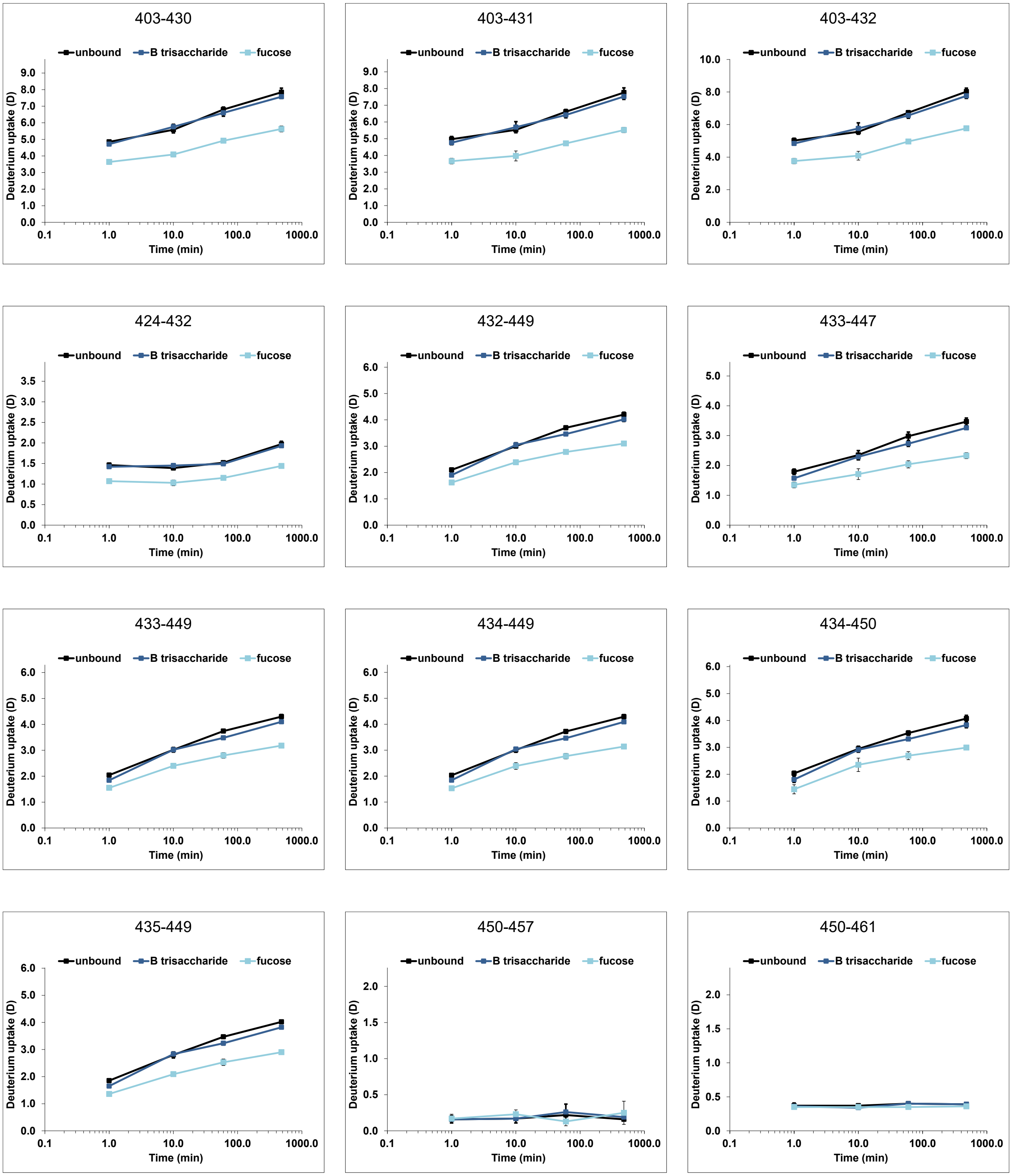

Figure S 30: continued

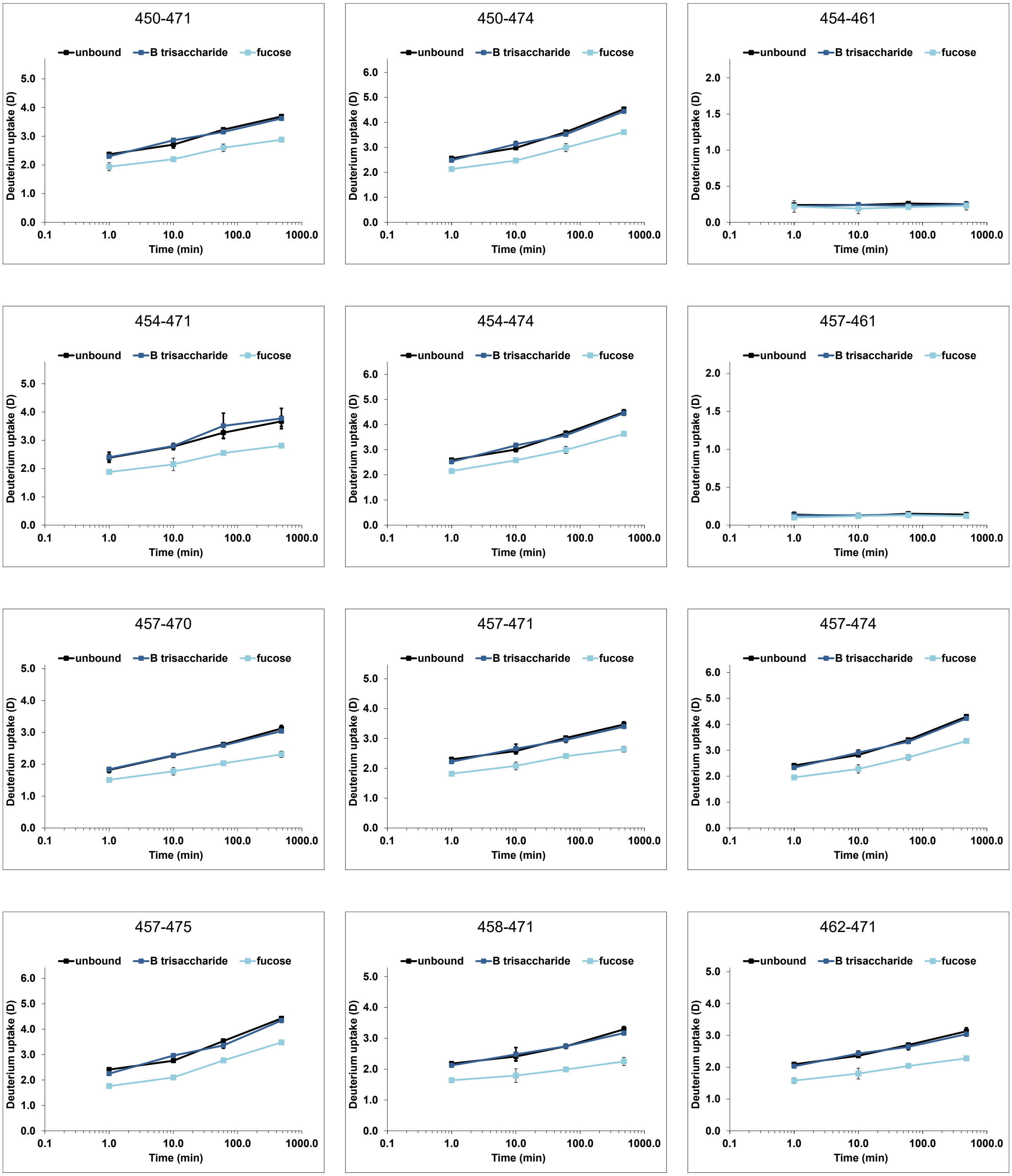

Figure S 30: continued

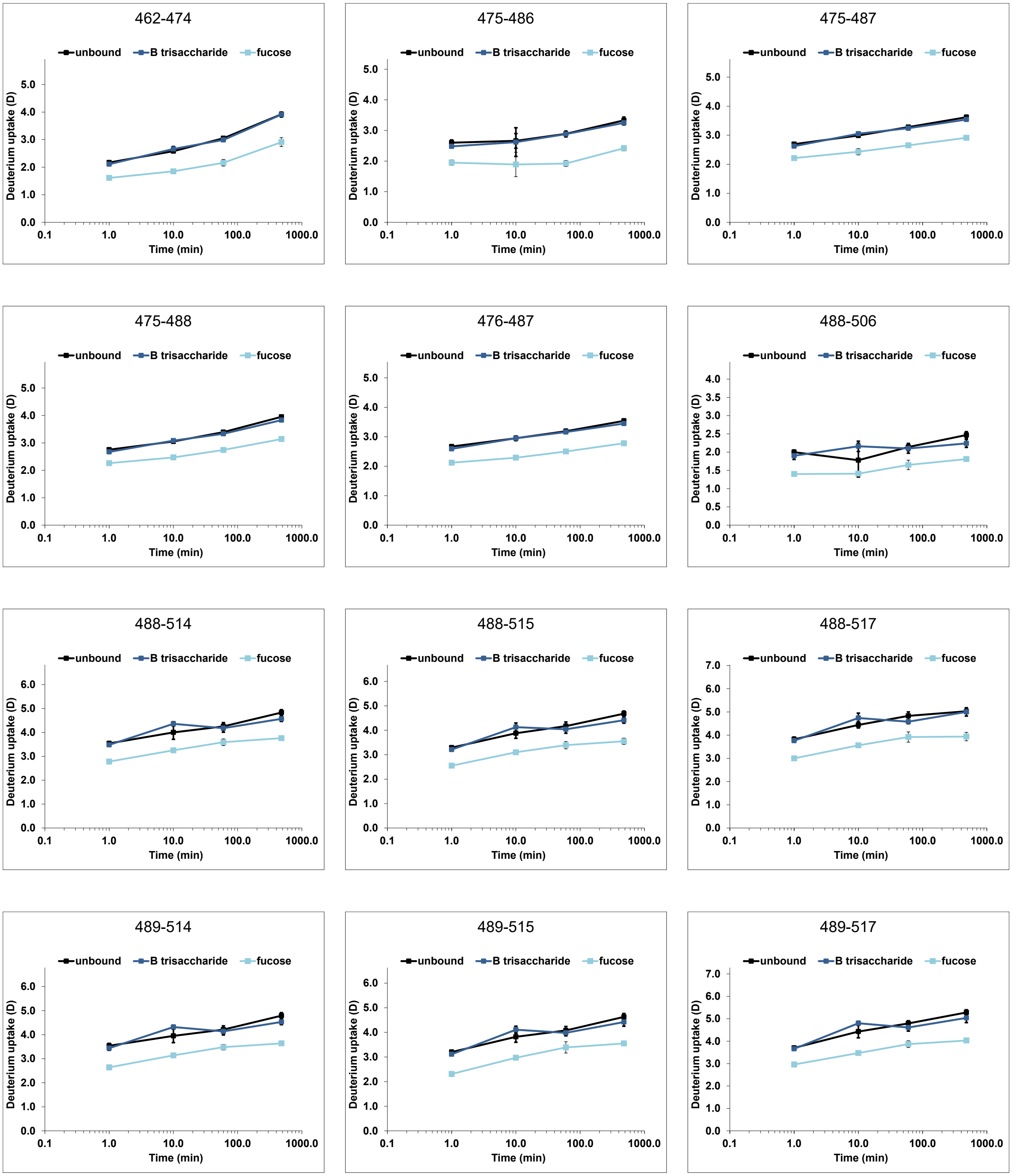

Figure S 30: continued

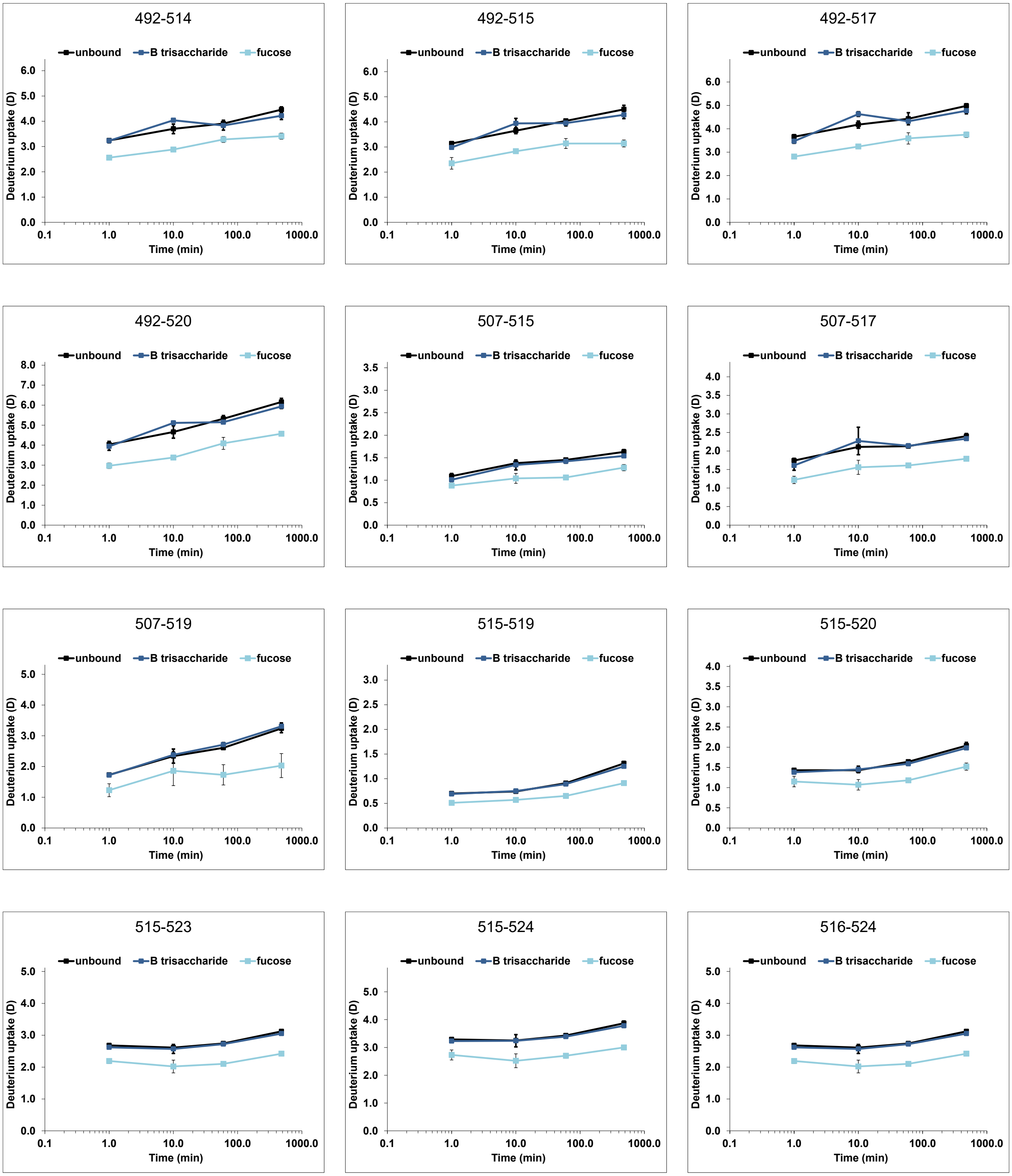

Figure S 30: continued

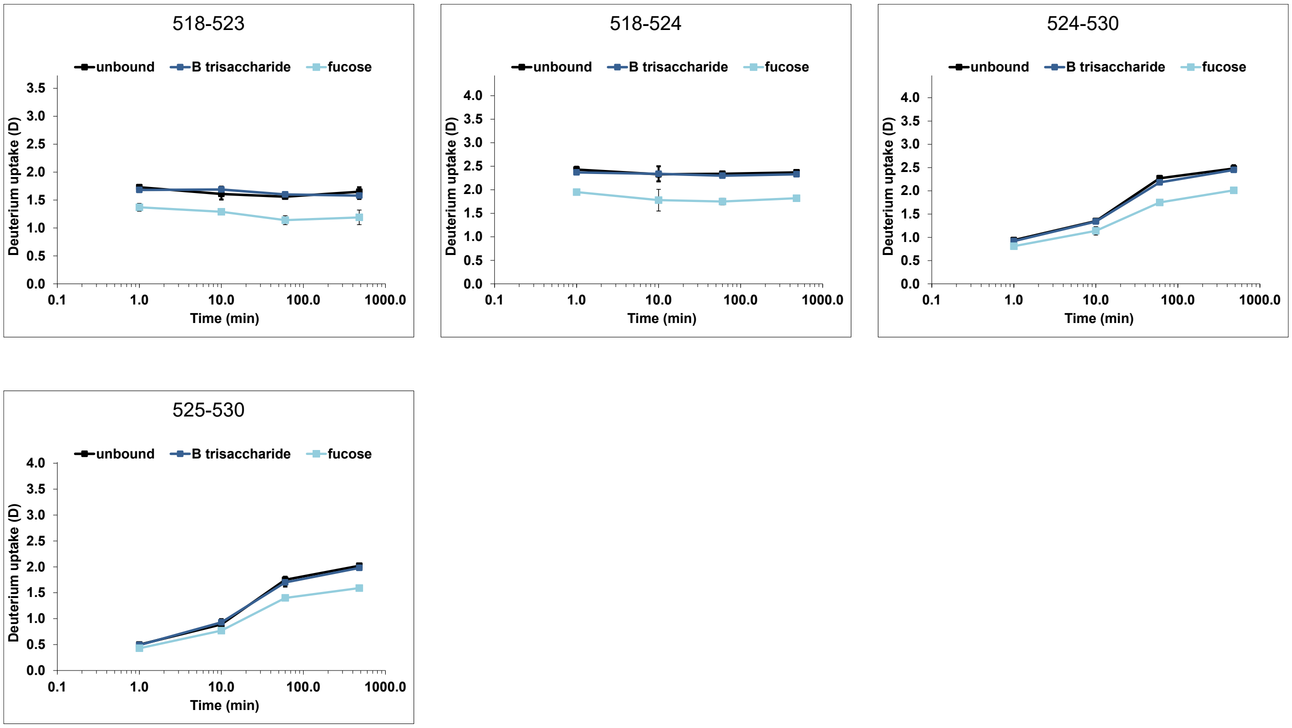

Figure S 30: continued

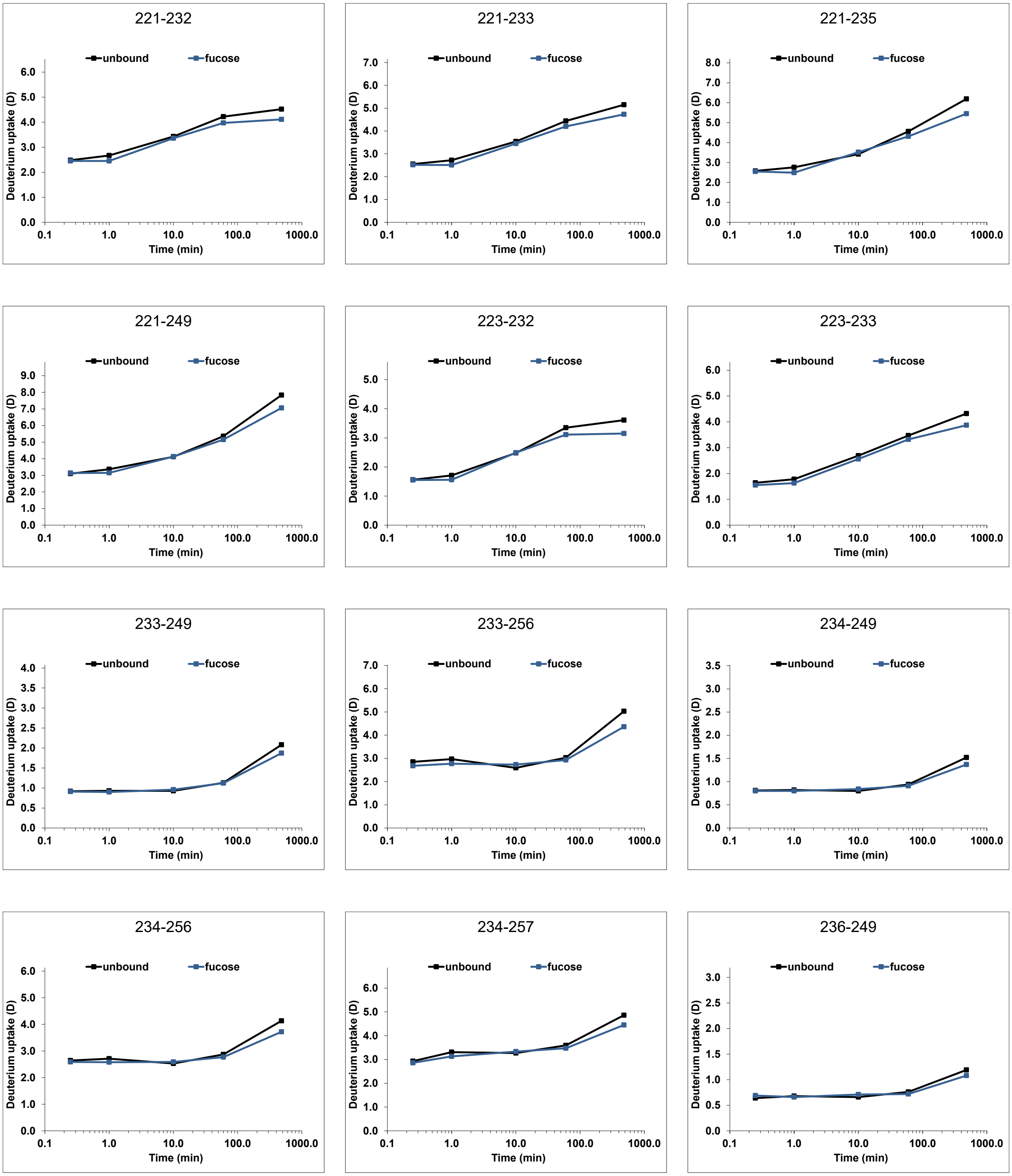

Figure S 31: Centroid deuterium uptake plots for wildtype MI001 P dimers (black) with 100 mM fucose (blue) (dataset T6, extended pepsin column wash). All time points (15s, 1 min, 10 min, 1 h and 8 h) were performed in single measurements. Error bars indicate the standard deviation (SD). Deuteration values can be falsely low in centroid analysis of bimodal spectra. Compare Figure S 13 and Table S 3 for assessment of statistically significant deuteration differences.

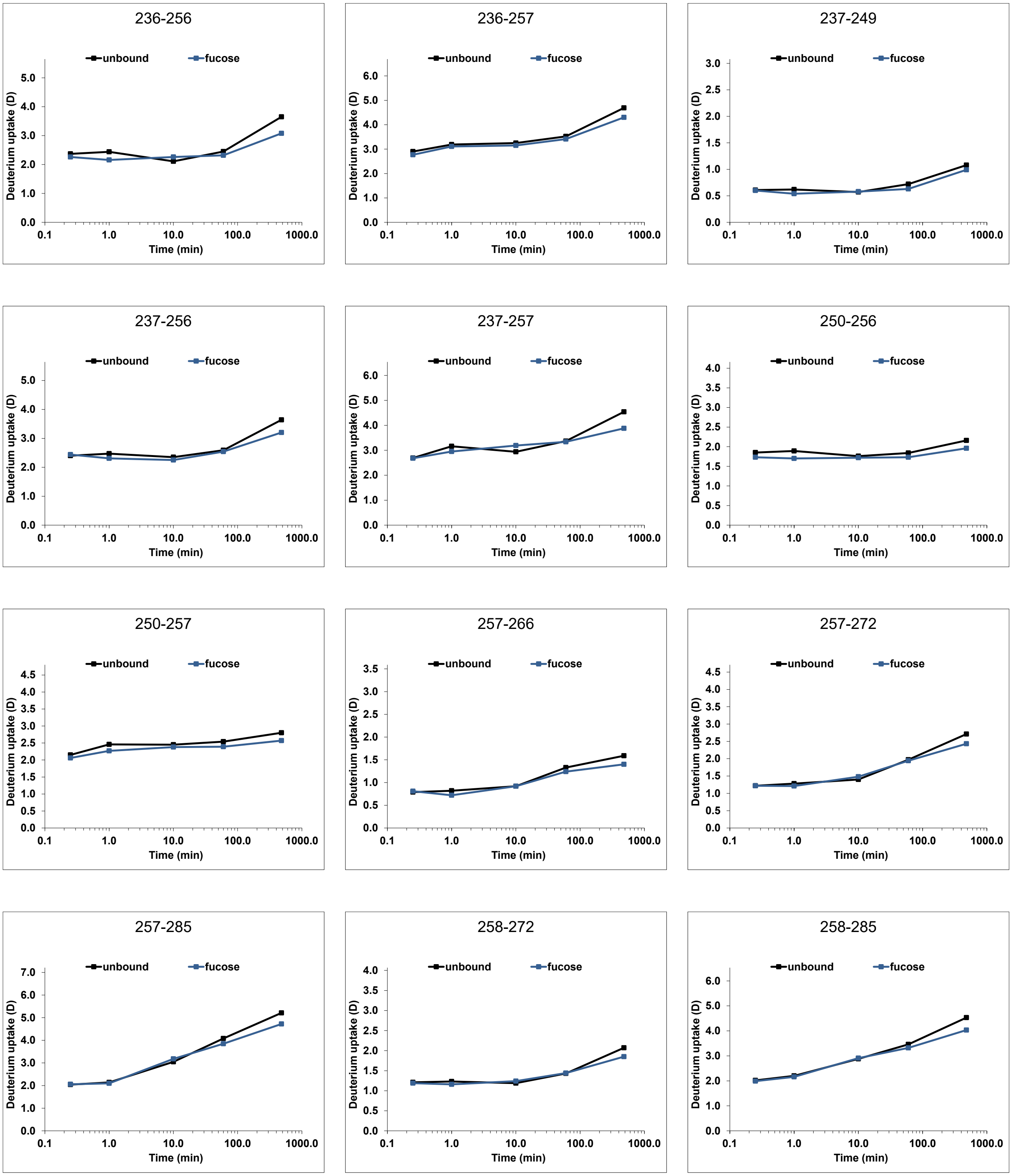

Figure S 31: continued

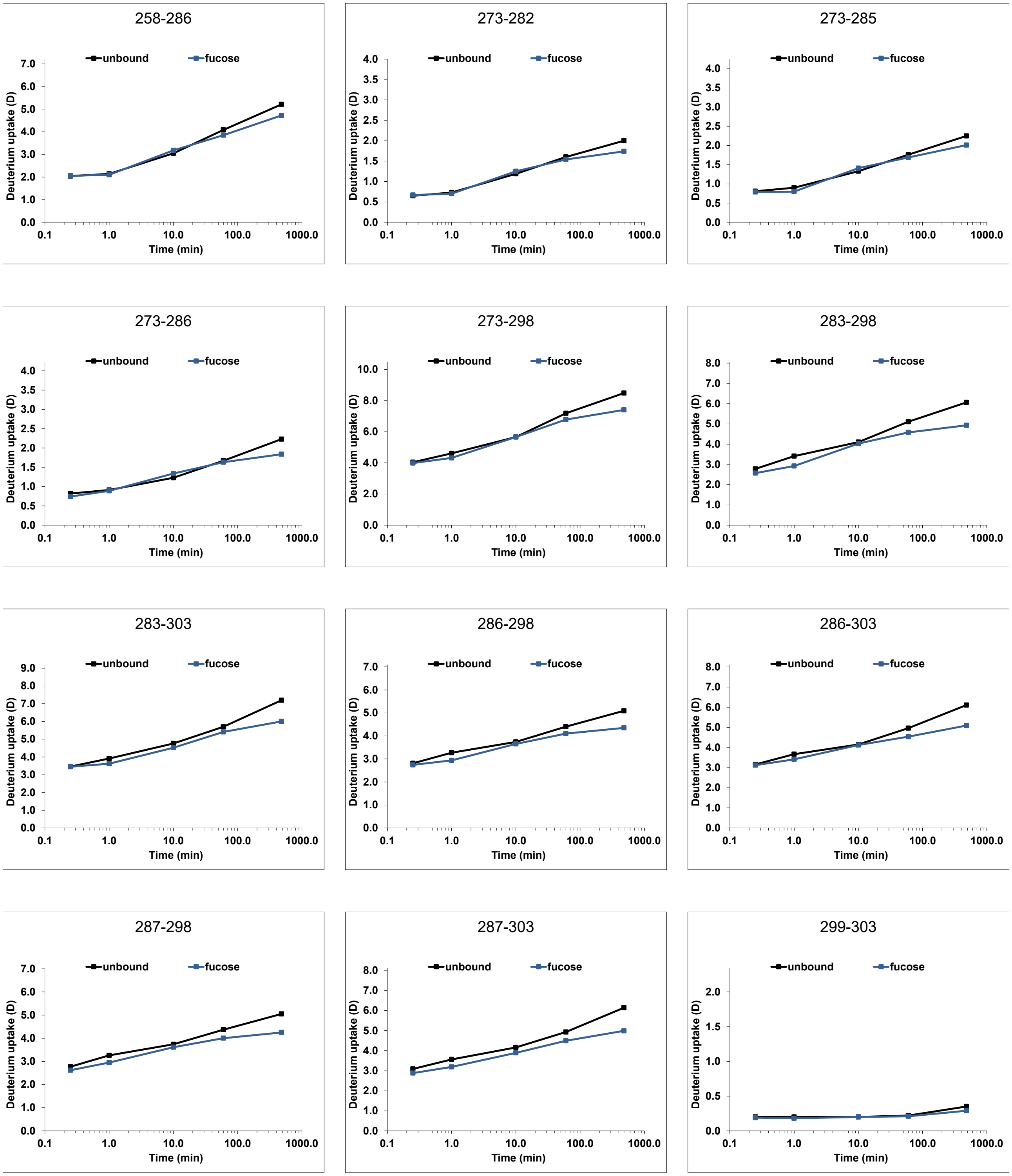

Figure S 31: continued

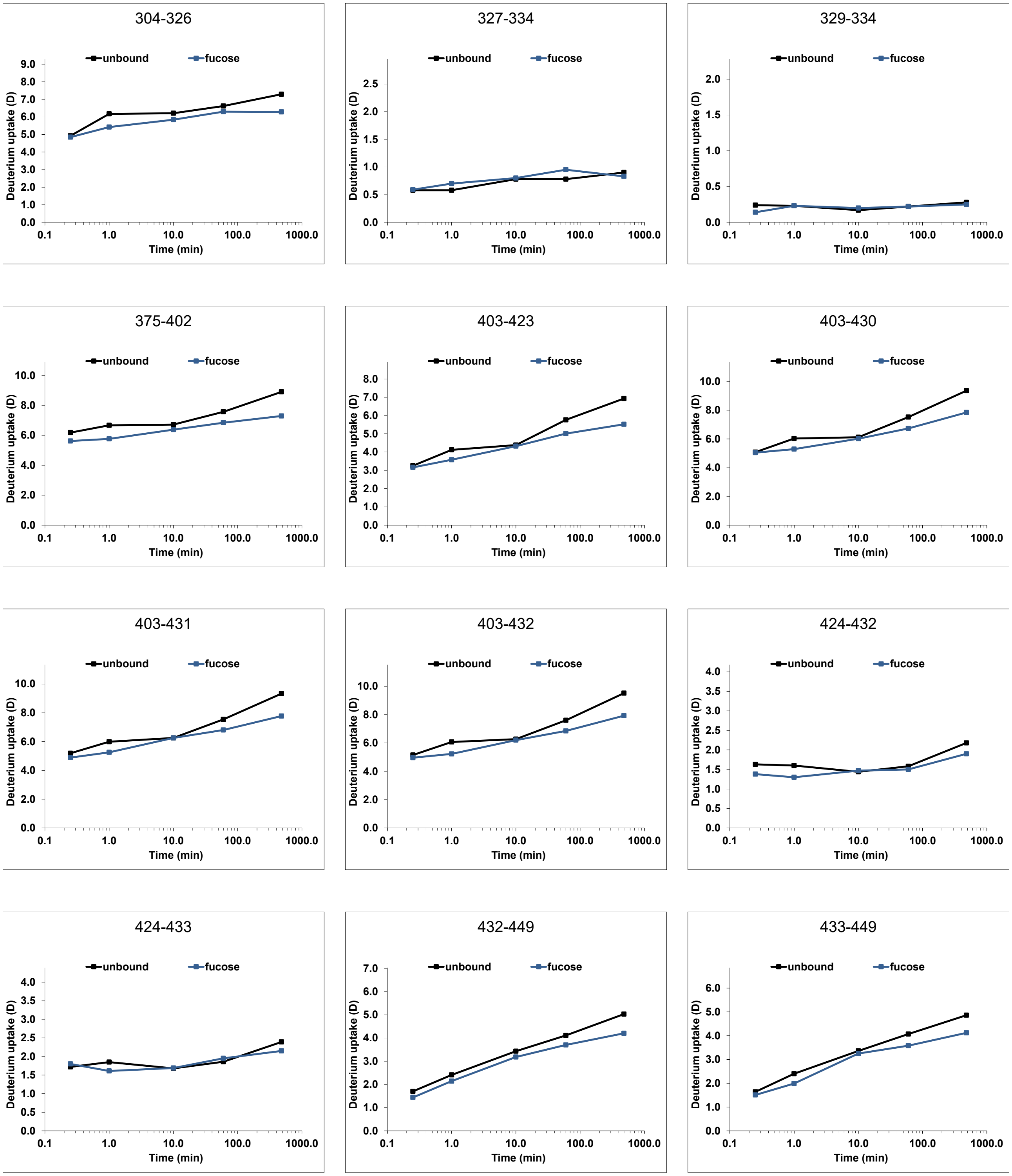

Figure S 31: continued

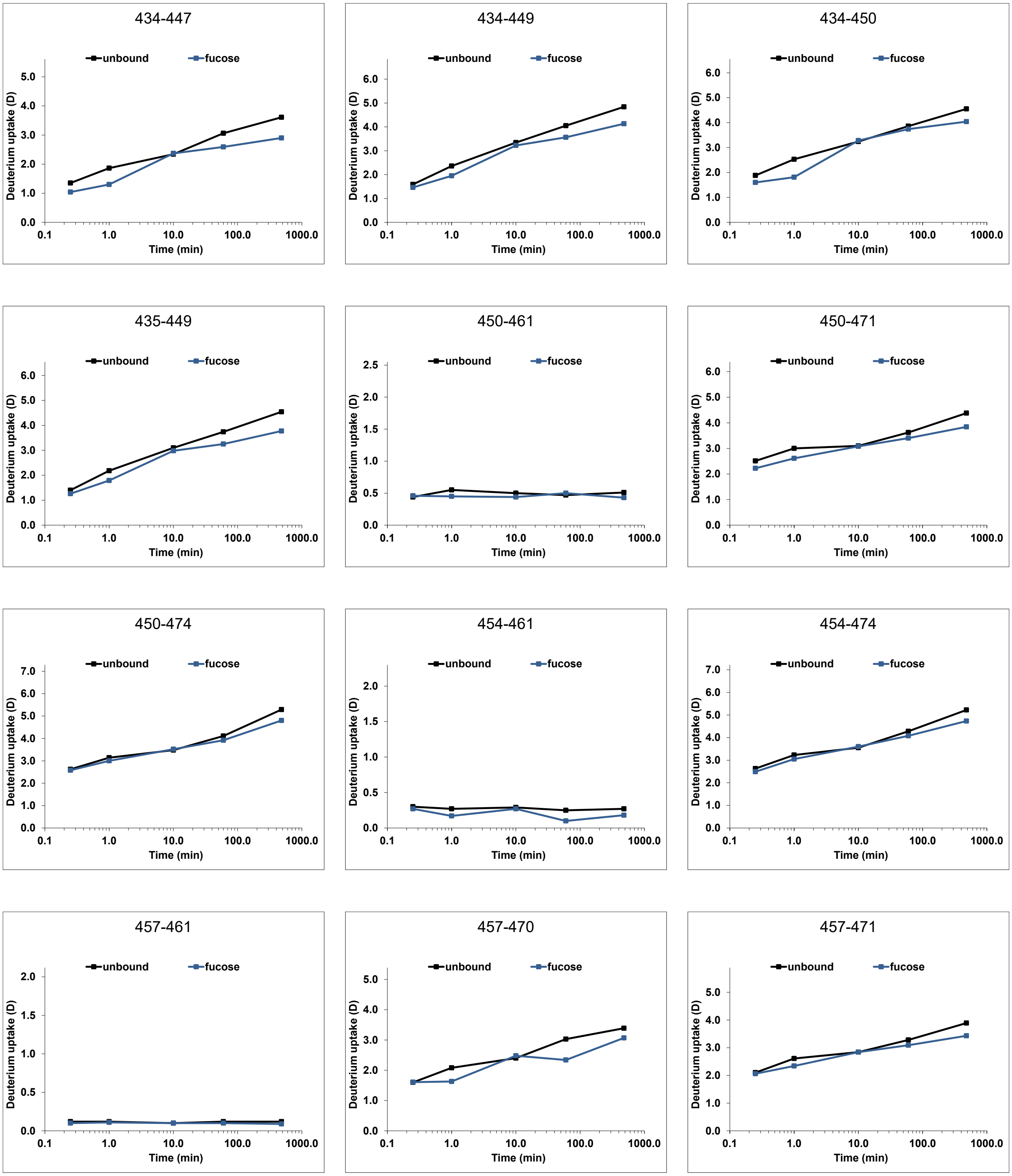

Figure S 31: continued

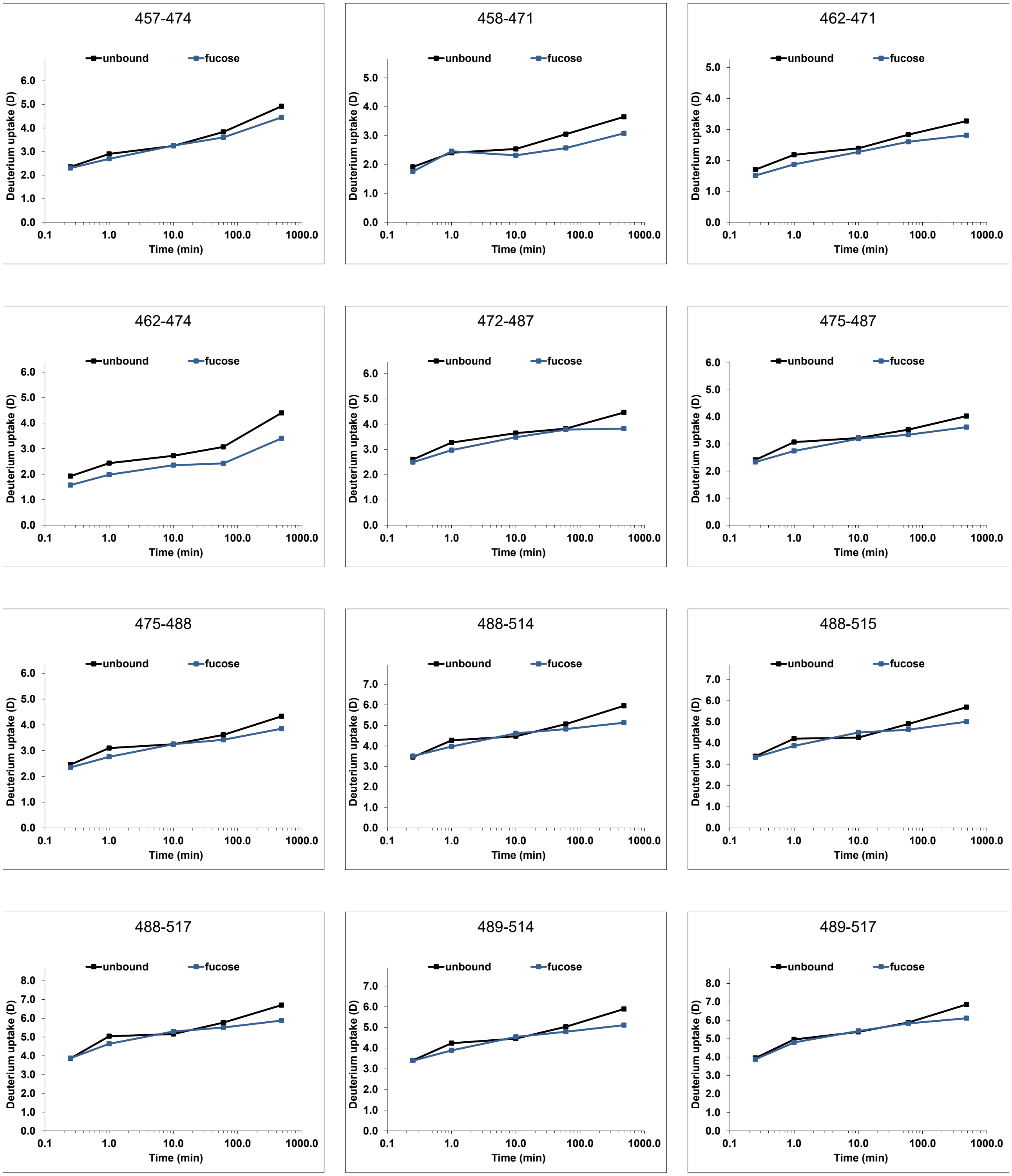

Figure S 31: continued

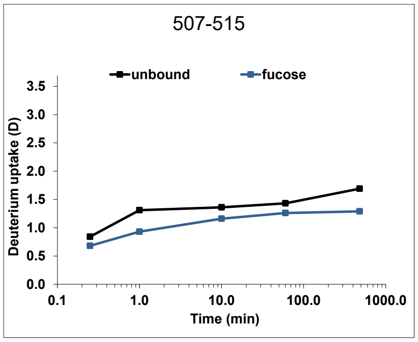

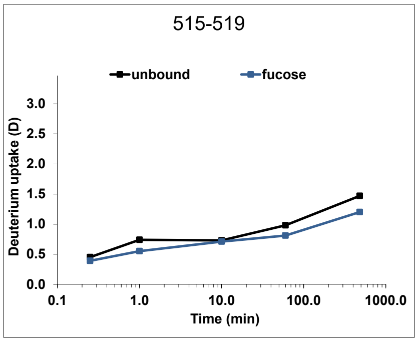

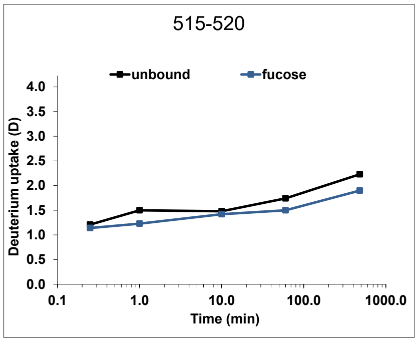

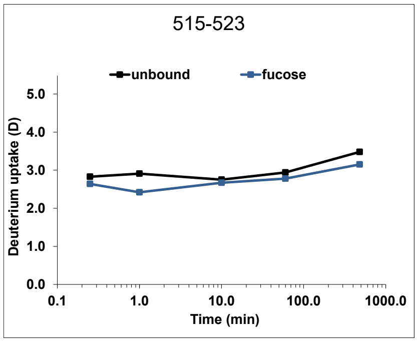

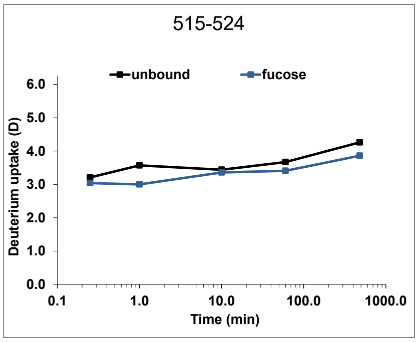

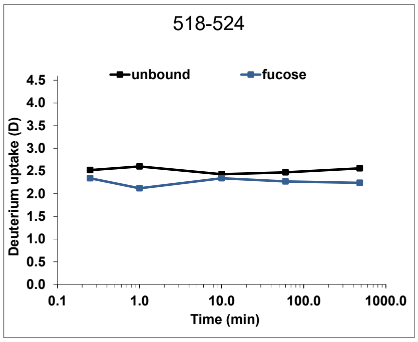

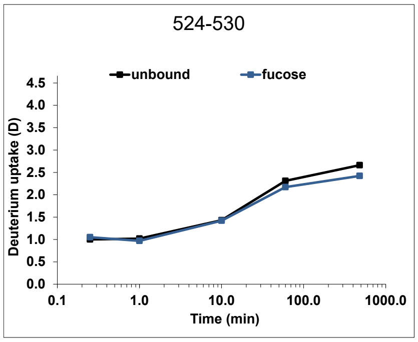

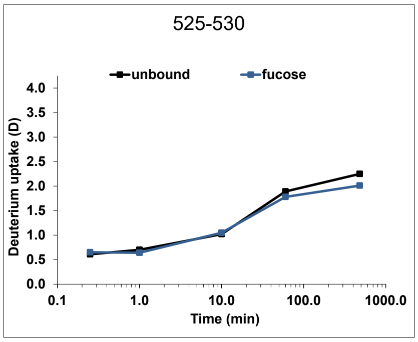

Figure S 31: continued

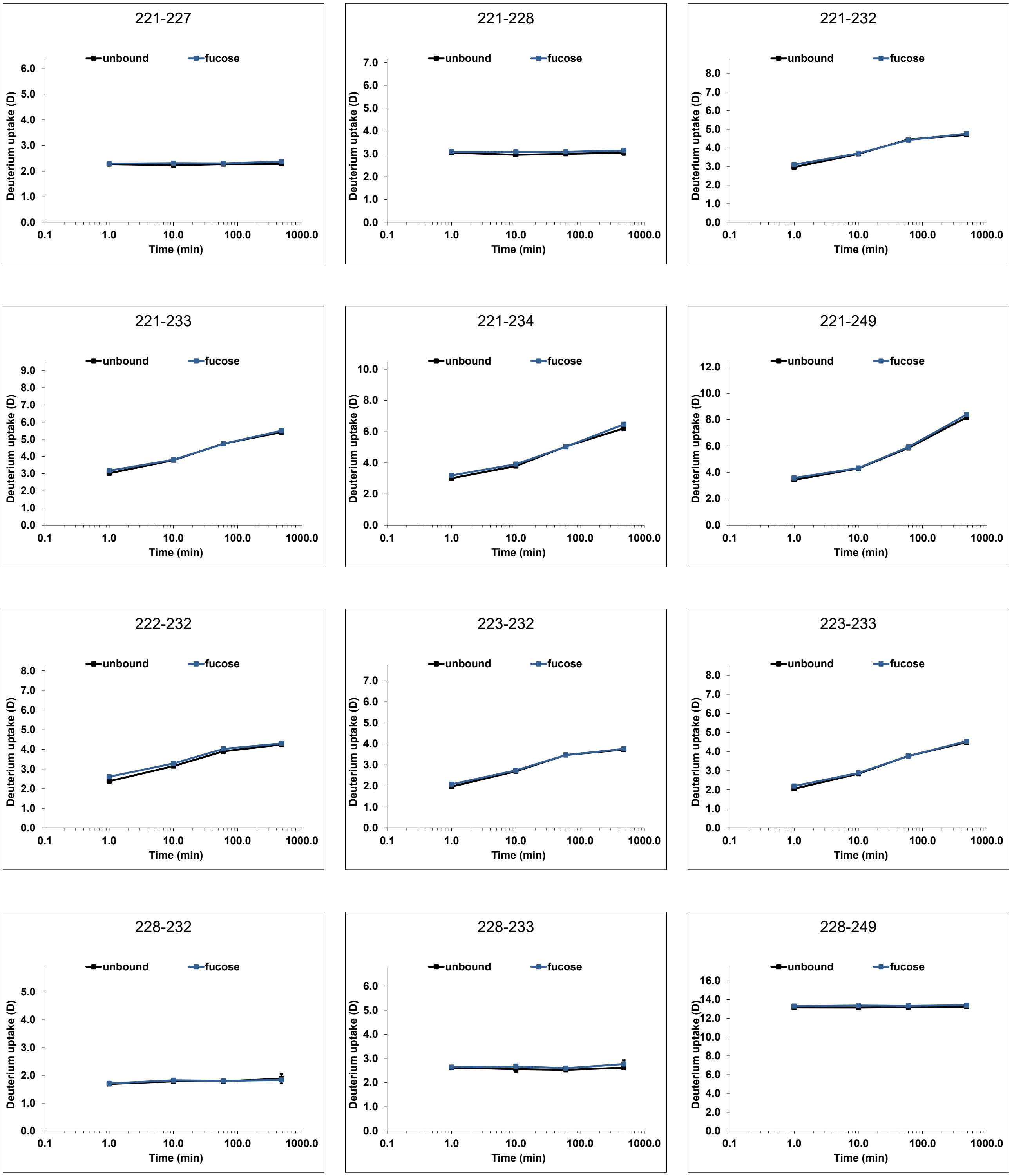

Figure S 32: Centroid deuterium uptake plots for partially deamidated MI001 P dimers (black) with 100 mM fucose (blue) (dataset T7). All time points (1 min, 10 min, 1 h and 8 h) were performed in triplicate. Error bars indicate the standard deviation (SD). Deuteration values can be falsely low in centroid analysis of bimodal spectra. Compare Figure S 12 and Table S 3 for assessment of statistically significant deuteration differences.

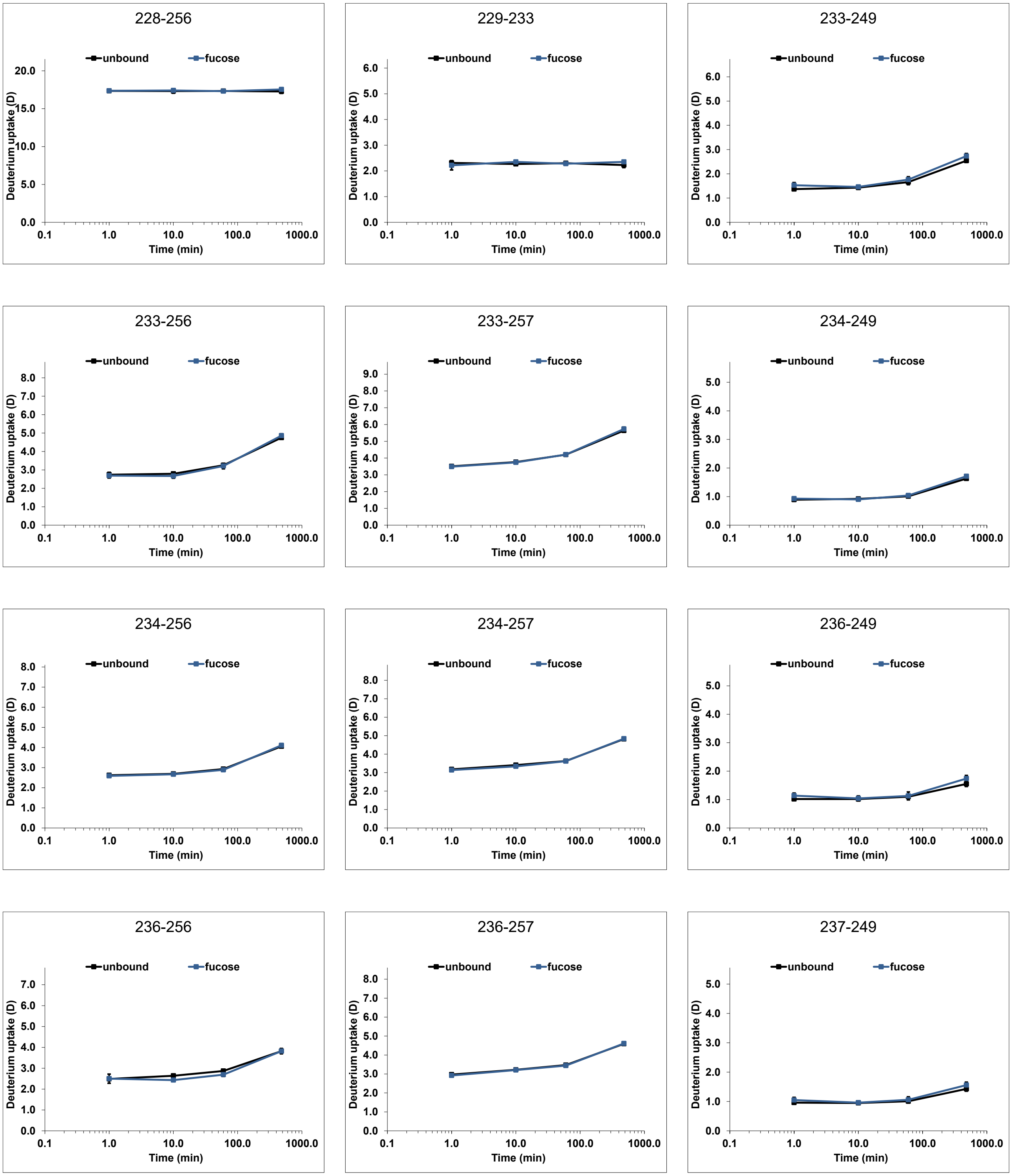

Figure S 32: continued

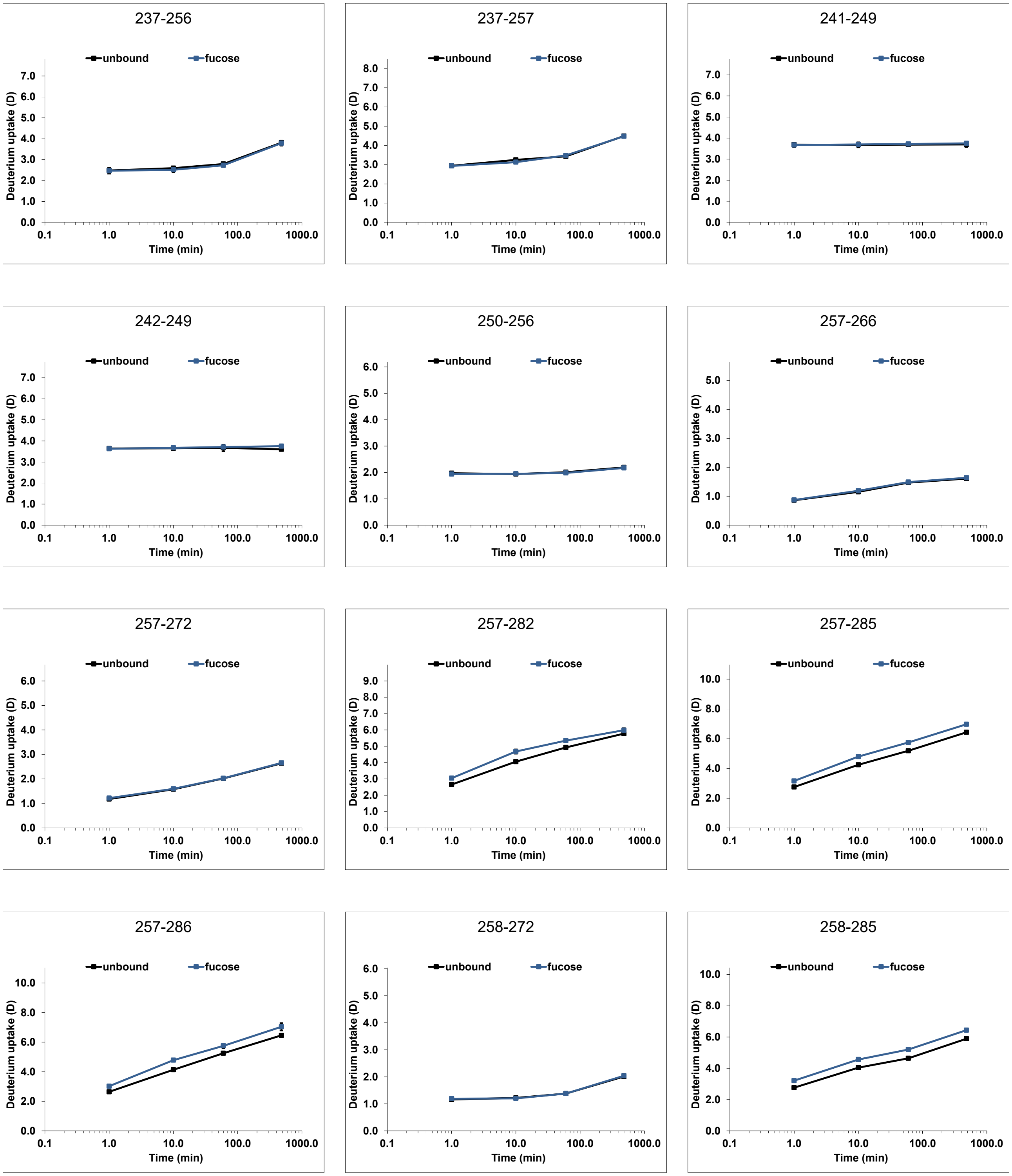

Figure S 32: continued

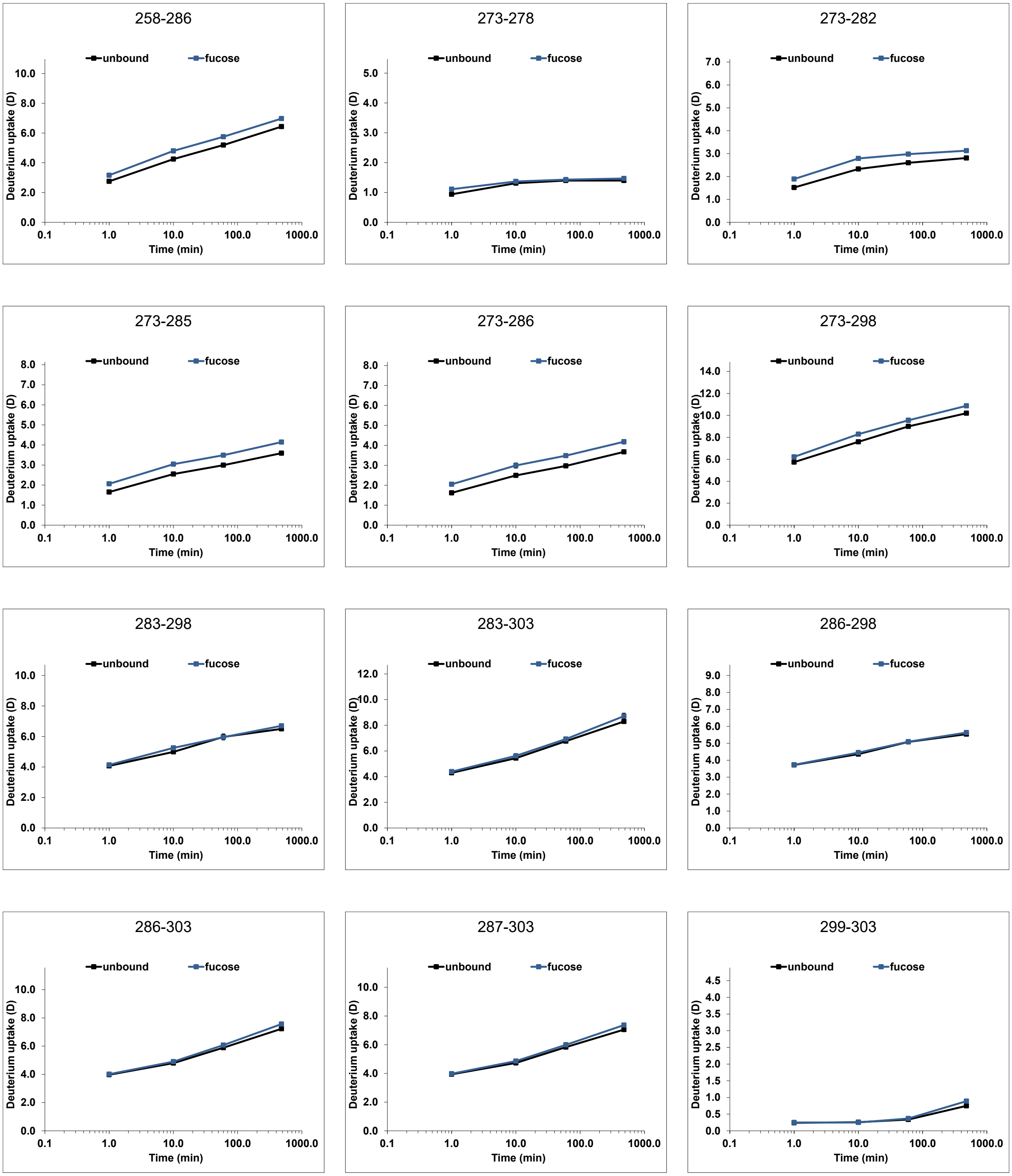

Figure S 32: continued

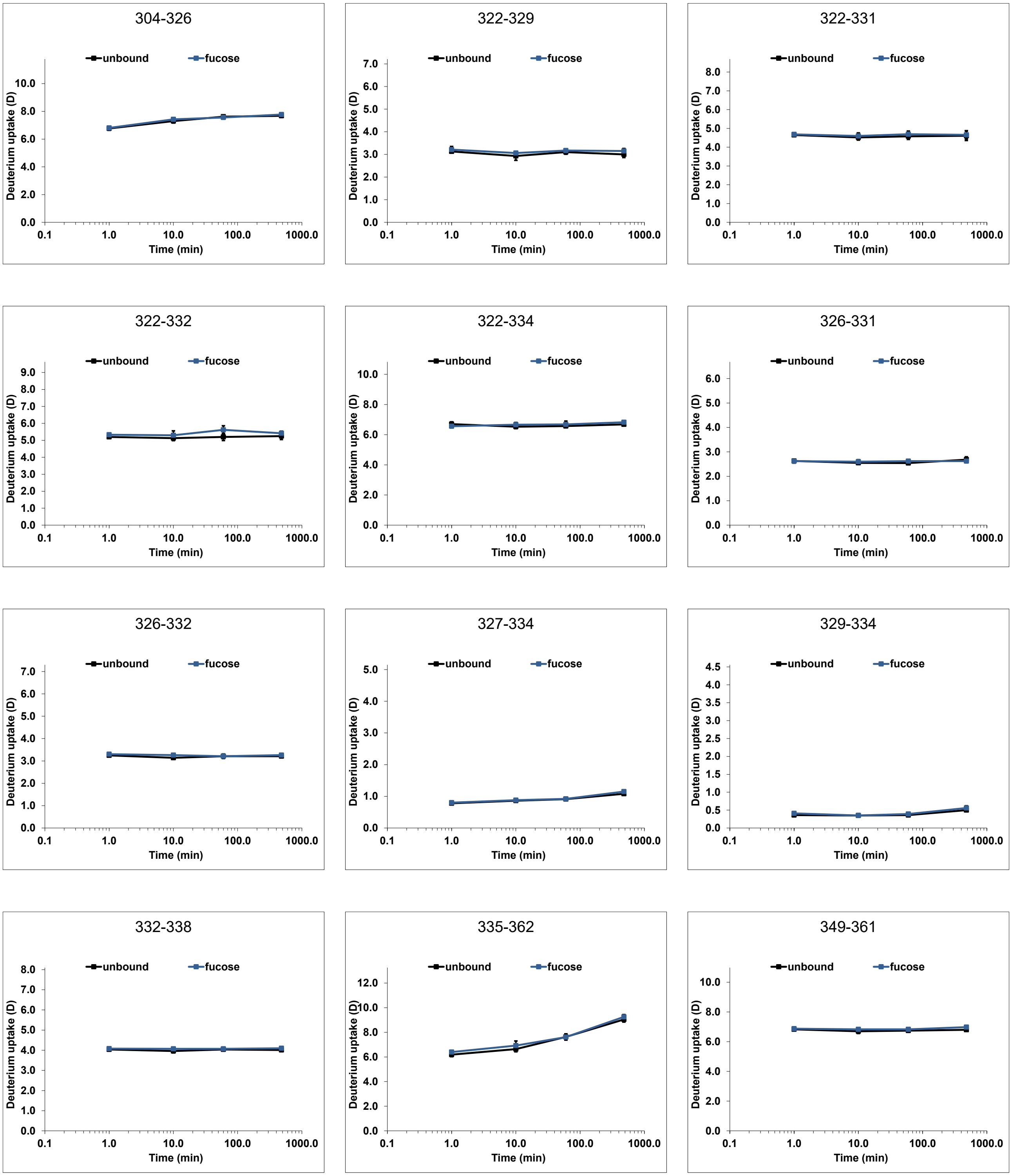

Figure S 32: continued

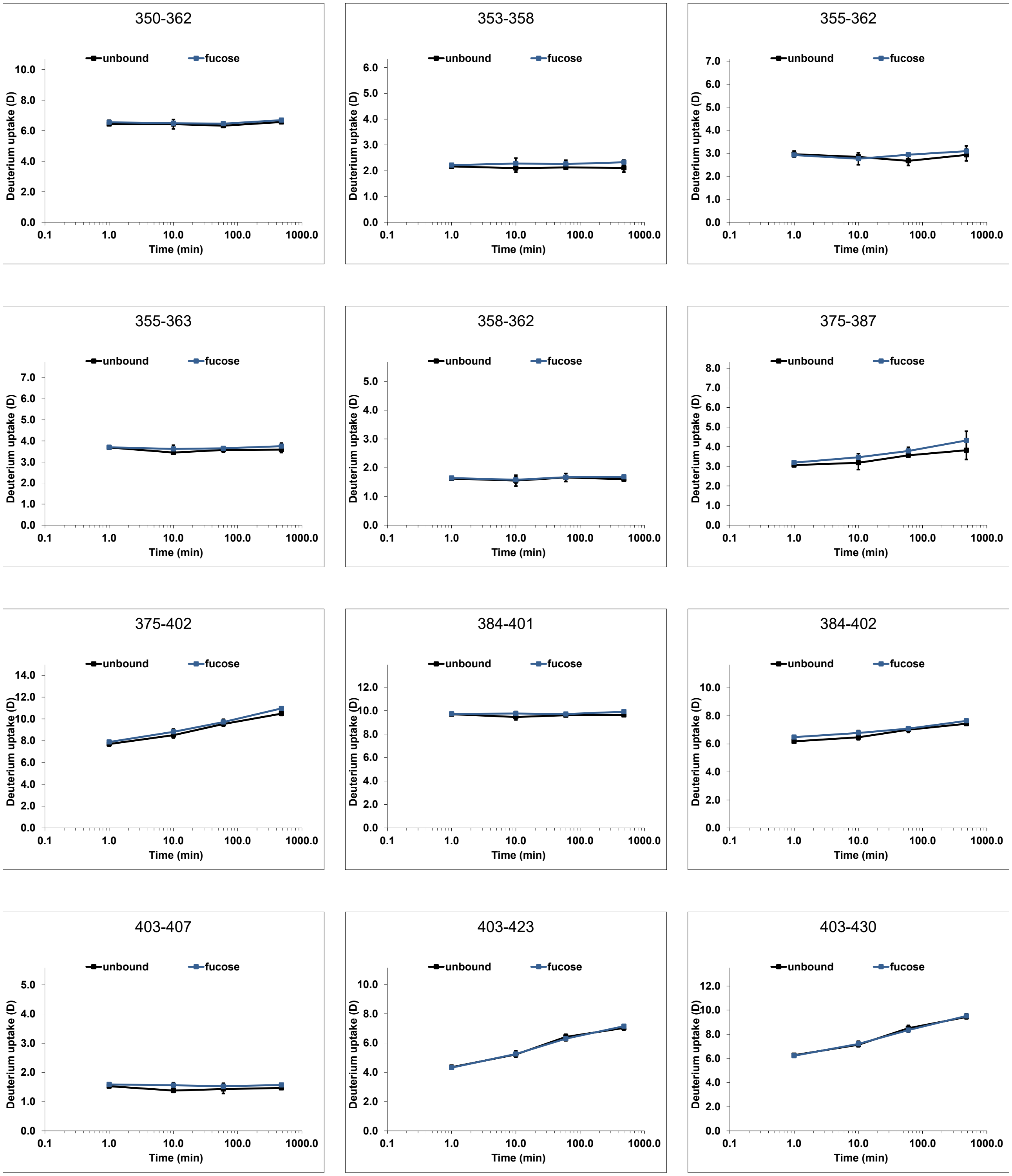

Figure S 32: continued

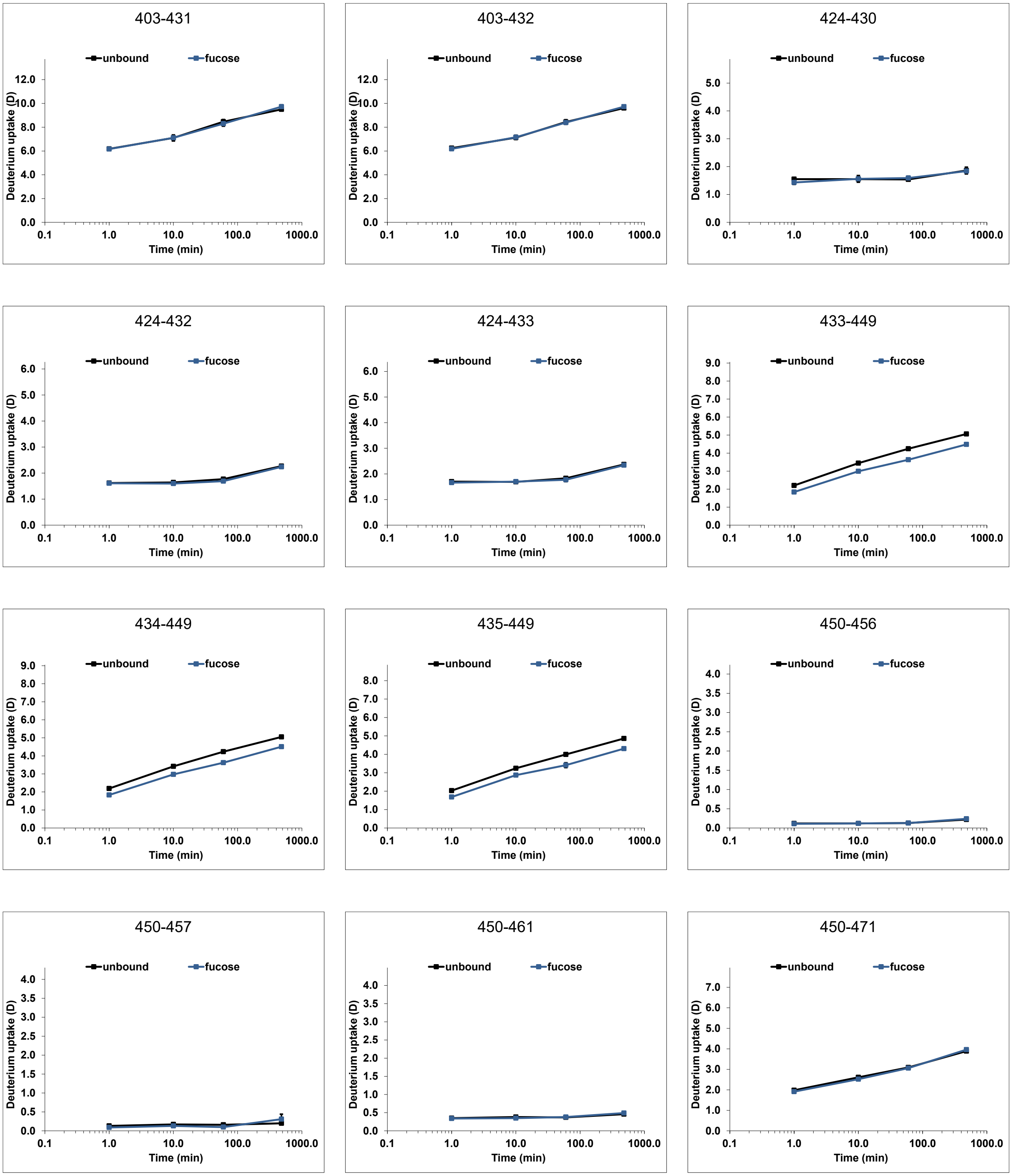

Figure S 32: continued

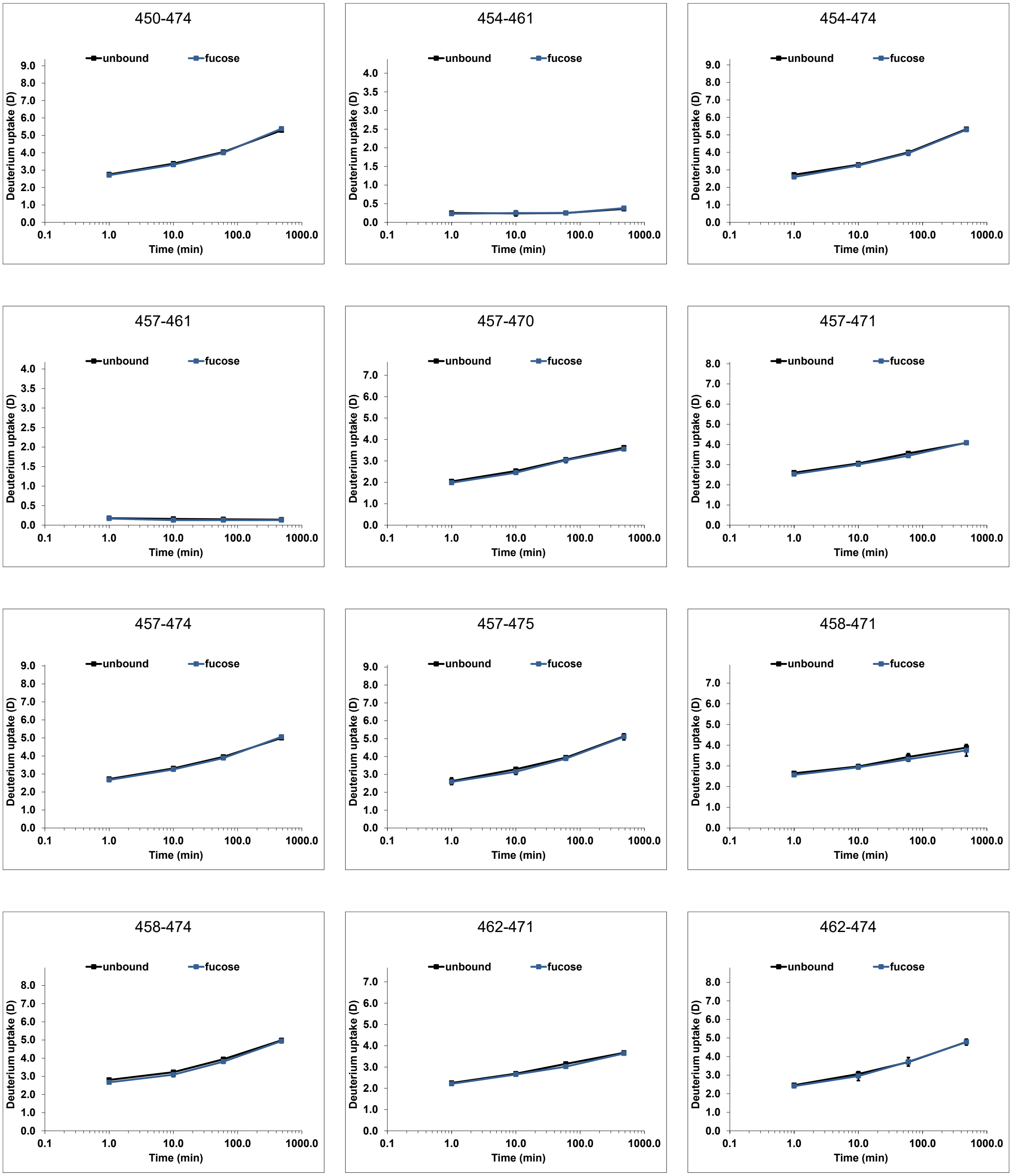

Figure S 32: continued

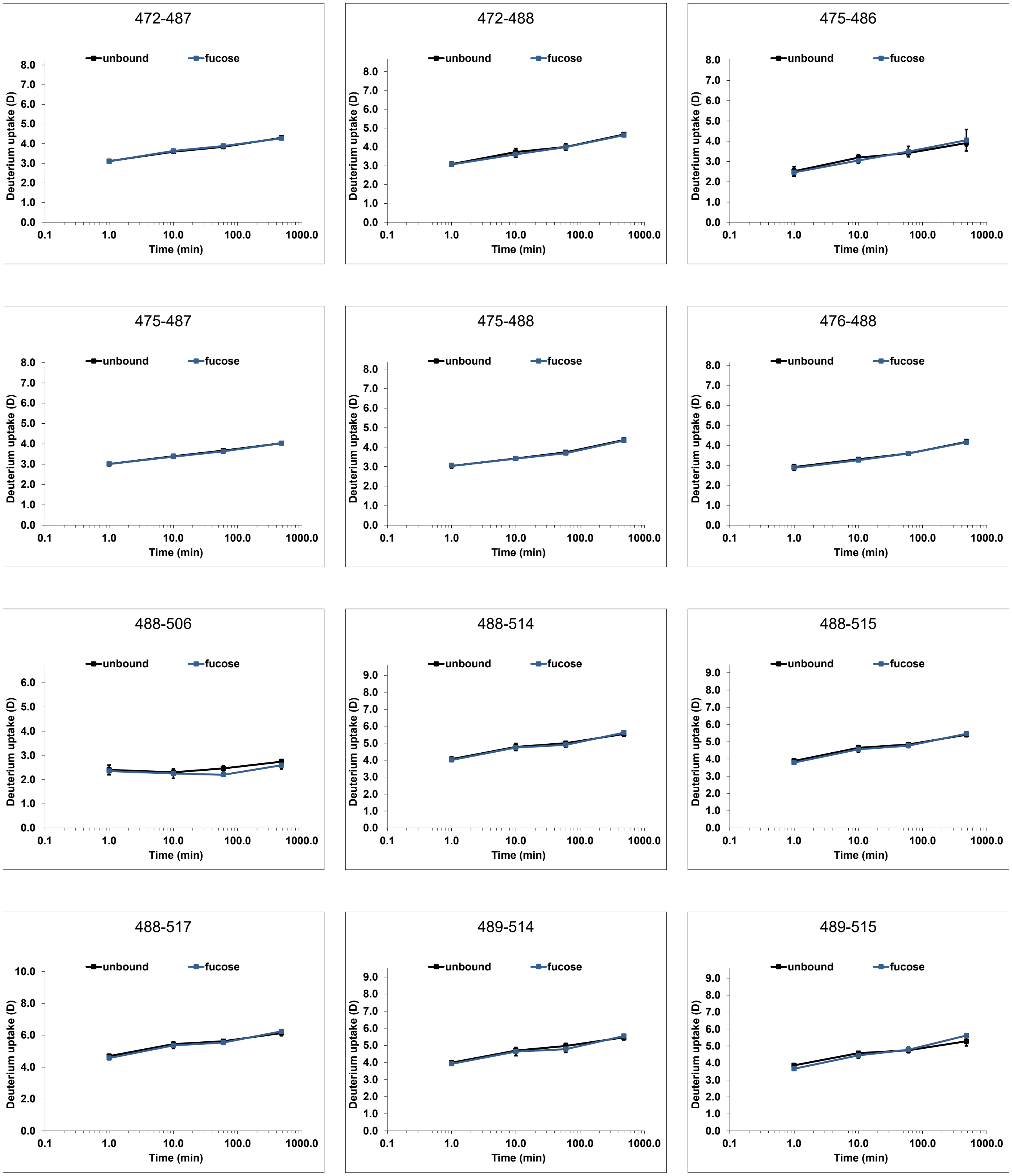

Figure S 32: continued

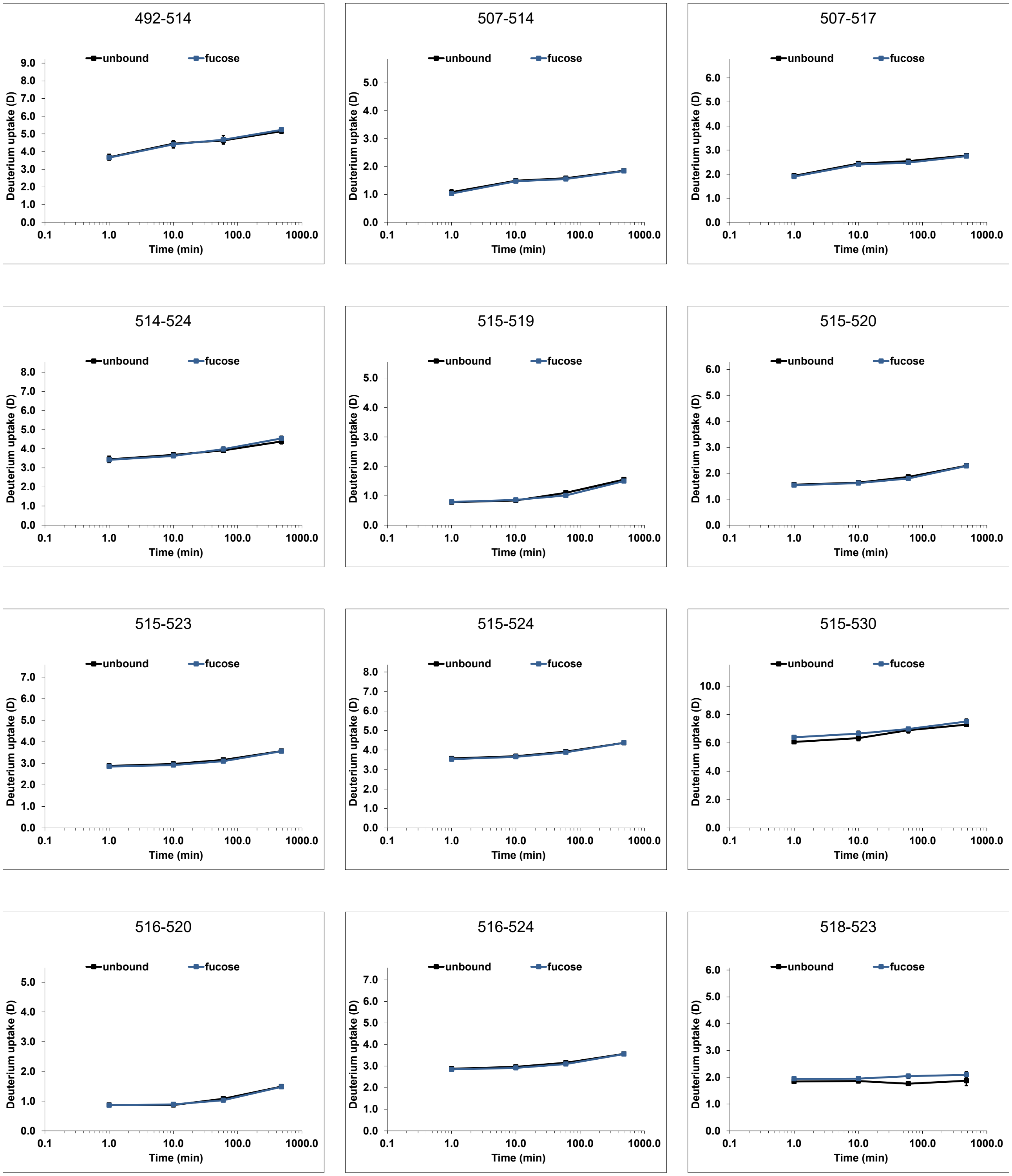

Figure S 32: continued

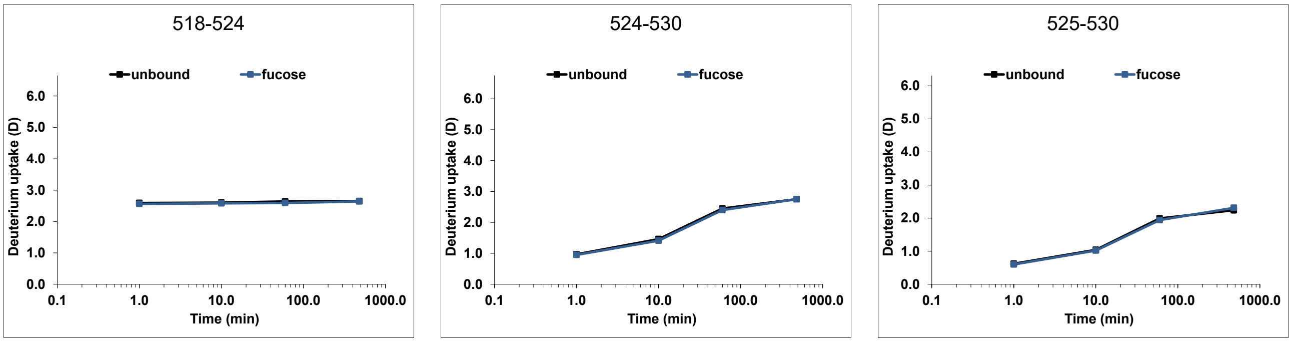

Figure S 32: continued

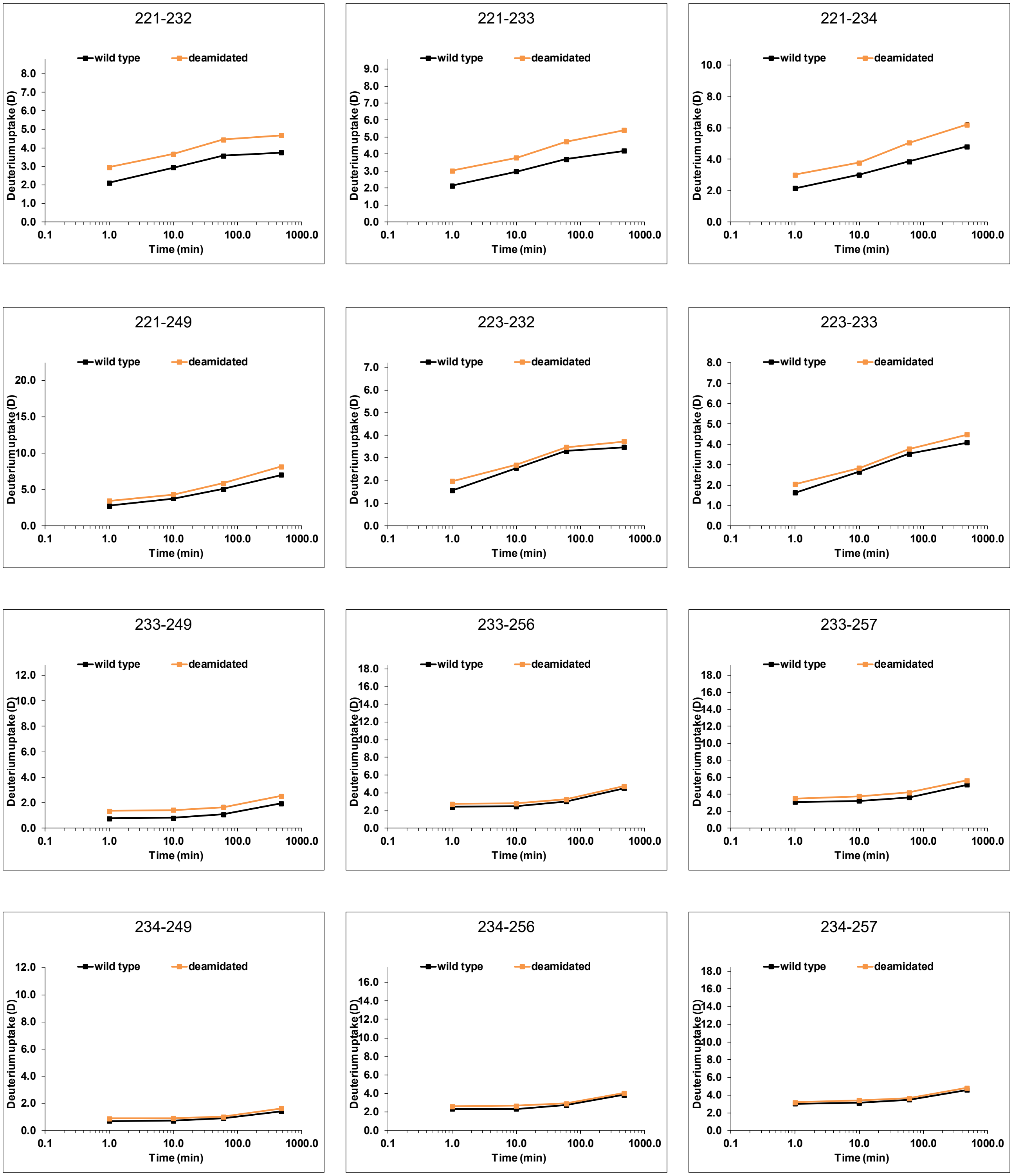

Figure S 33: Centroid deuterium uptake plot comparison for wild type (black) and partially deamidated MI001 P dimers (orange) without any ligand (dataset T8). All time points (1 min, 10 min, 1 h, 8 h) were performed in triplicate. The ratio of the FD controls from wildtype and deamidated measurements was used for normalization to account for different back exchange levels between the two datasets. Error bars indicate the standard deviation (SD). Deuteration values can be falsely low in centroid analysis of bimodal spectra. Compare Figure S 12 for assessment of statistically significant deuteration differences.

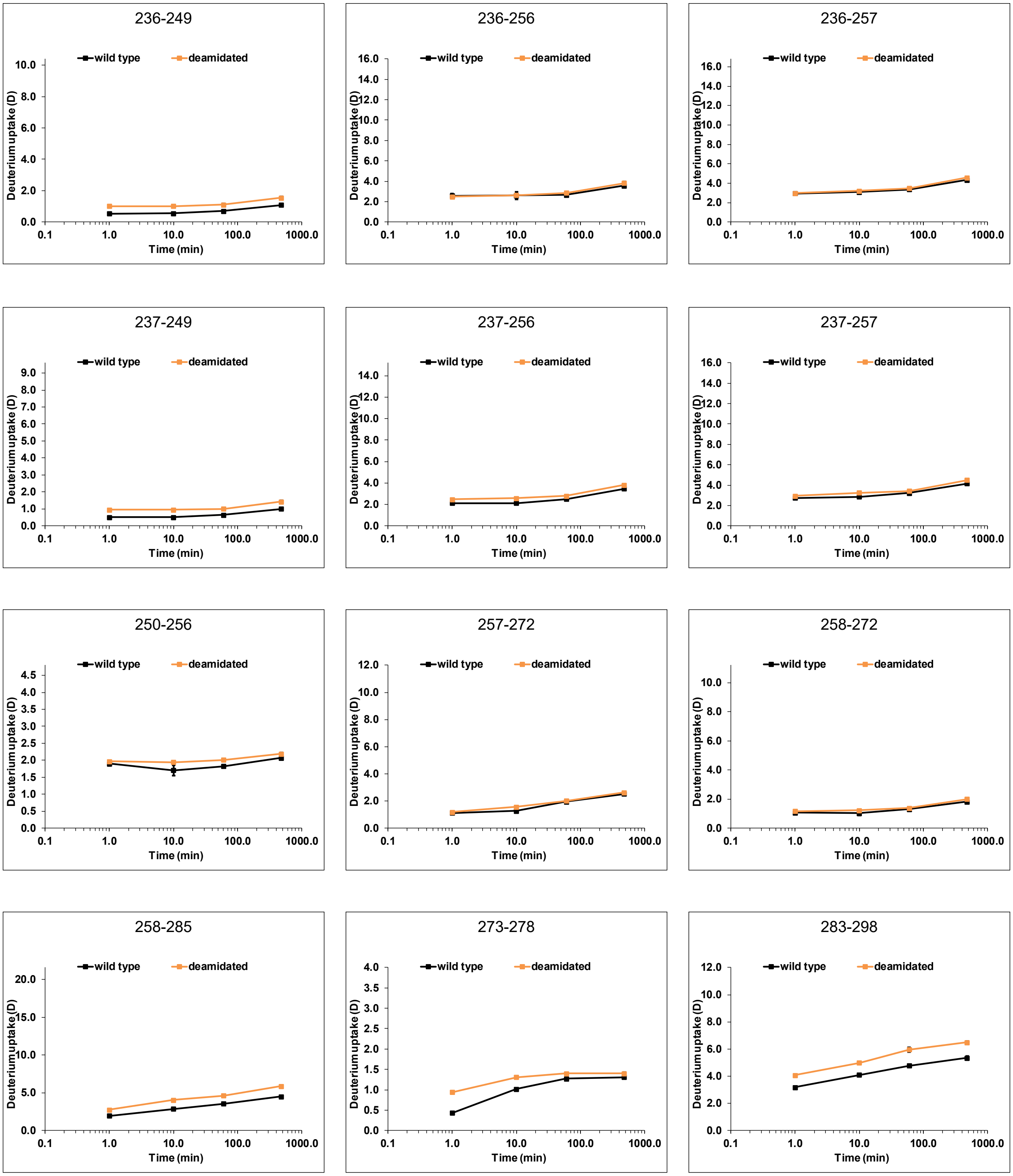

Figure S 33: continued

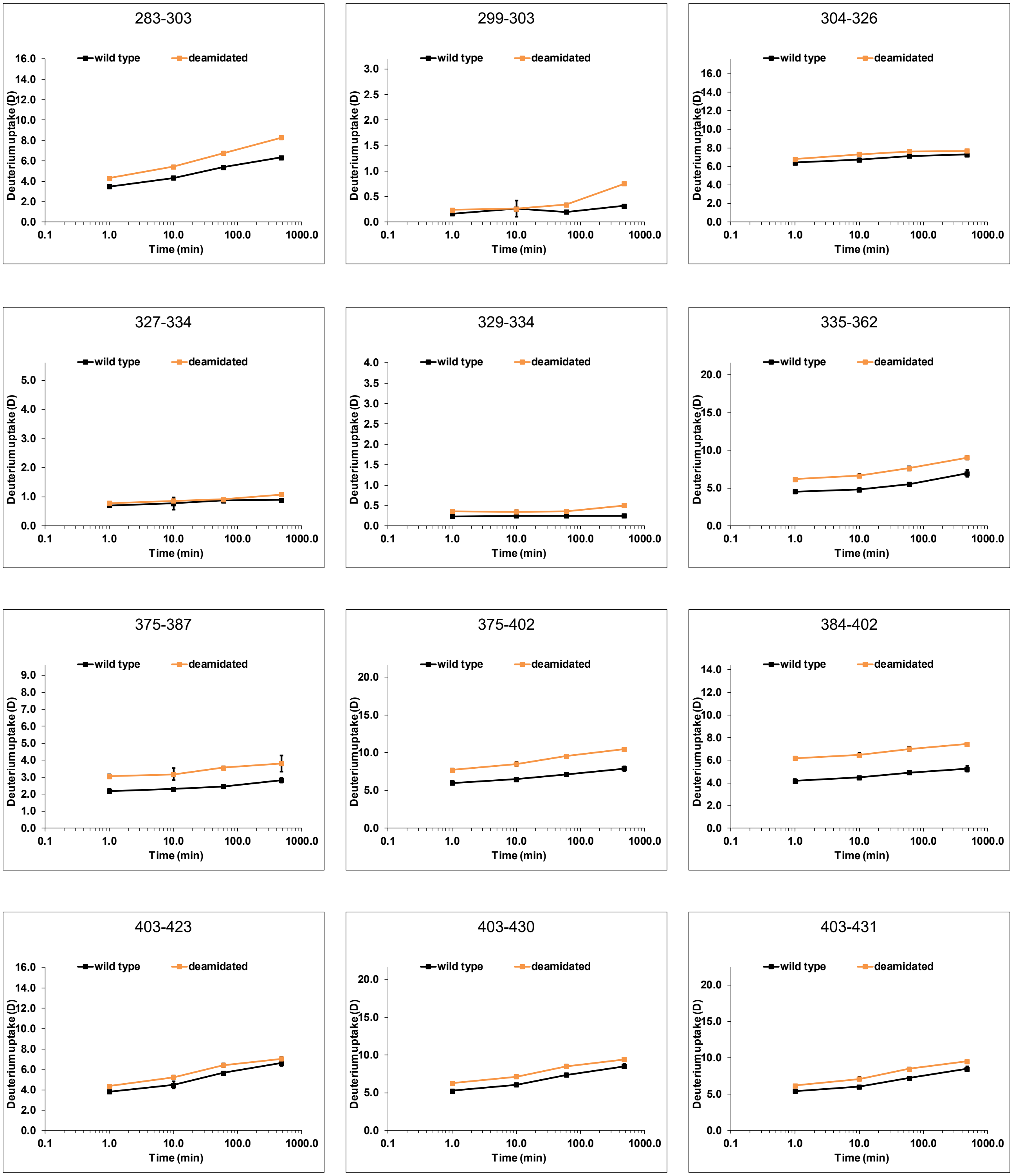

Figure S 33: continued

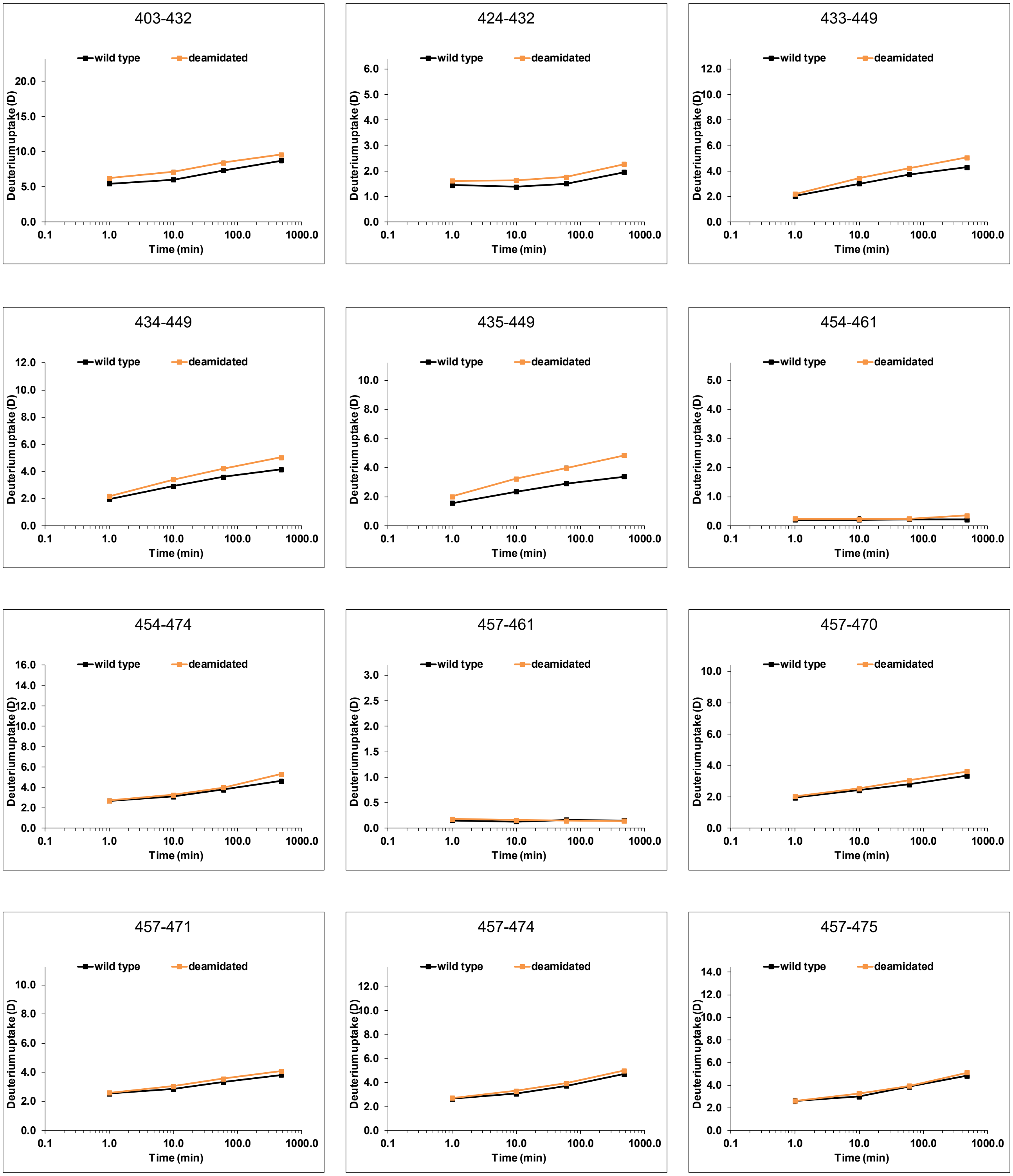

Figure S 33: continued

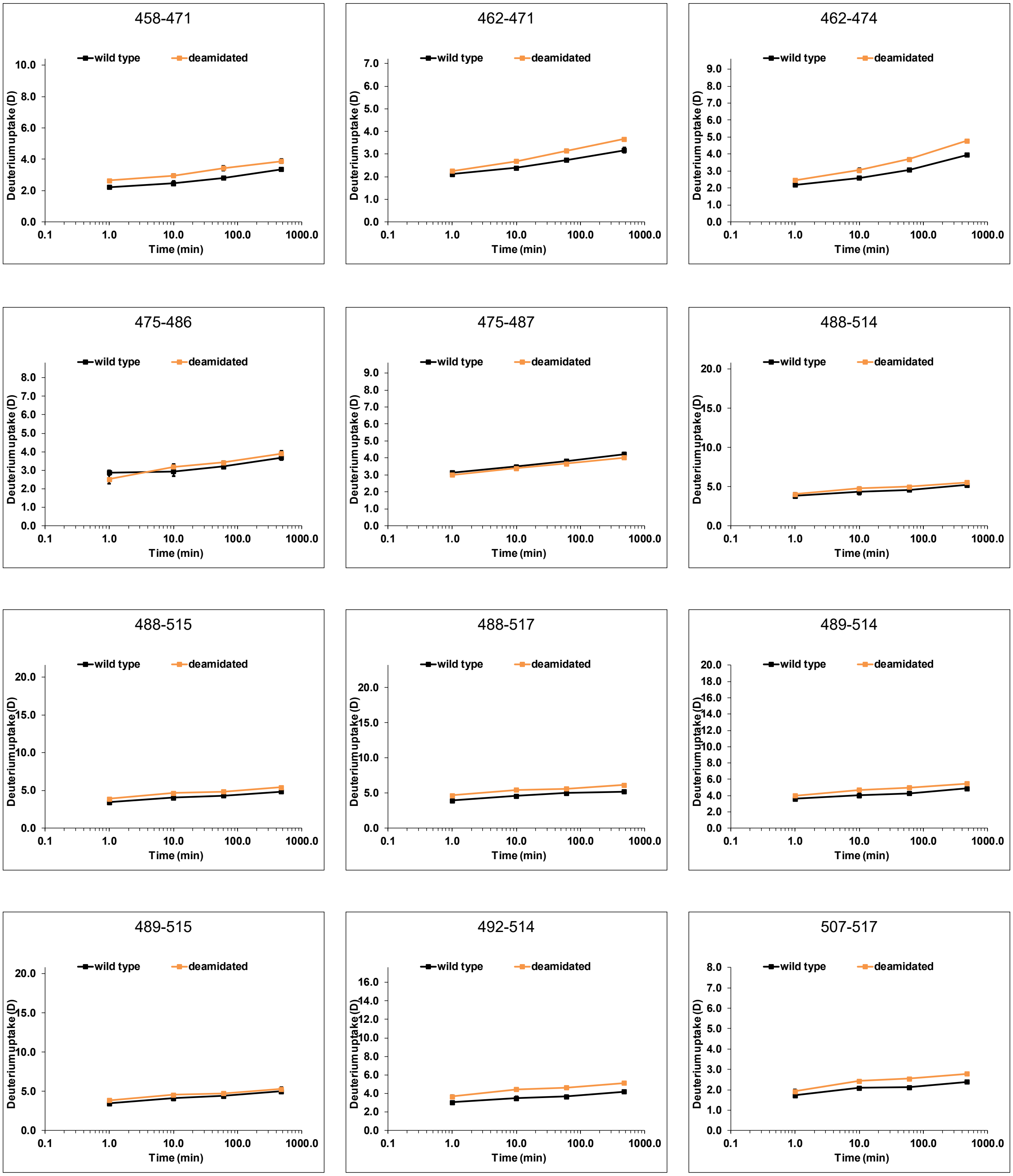

Figure S 33: continued

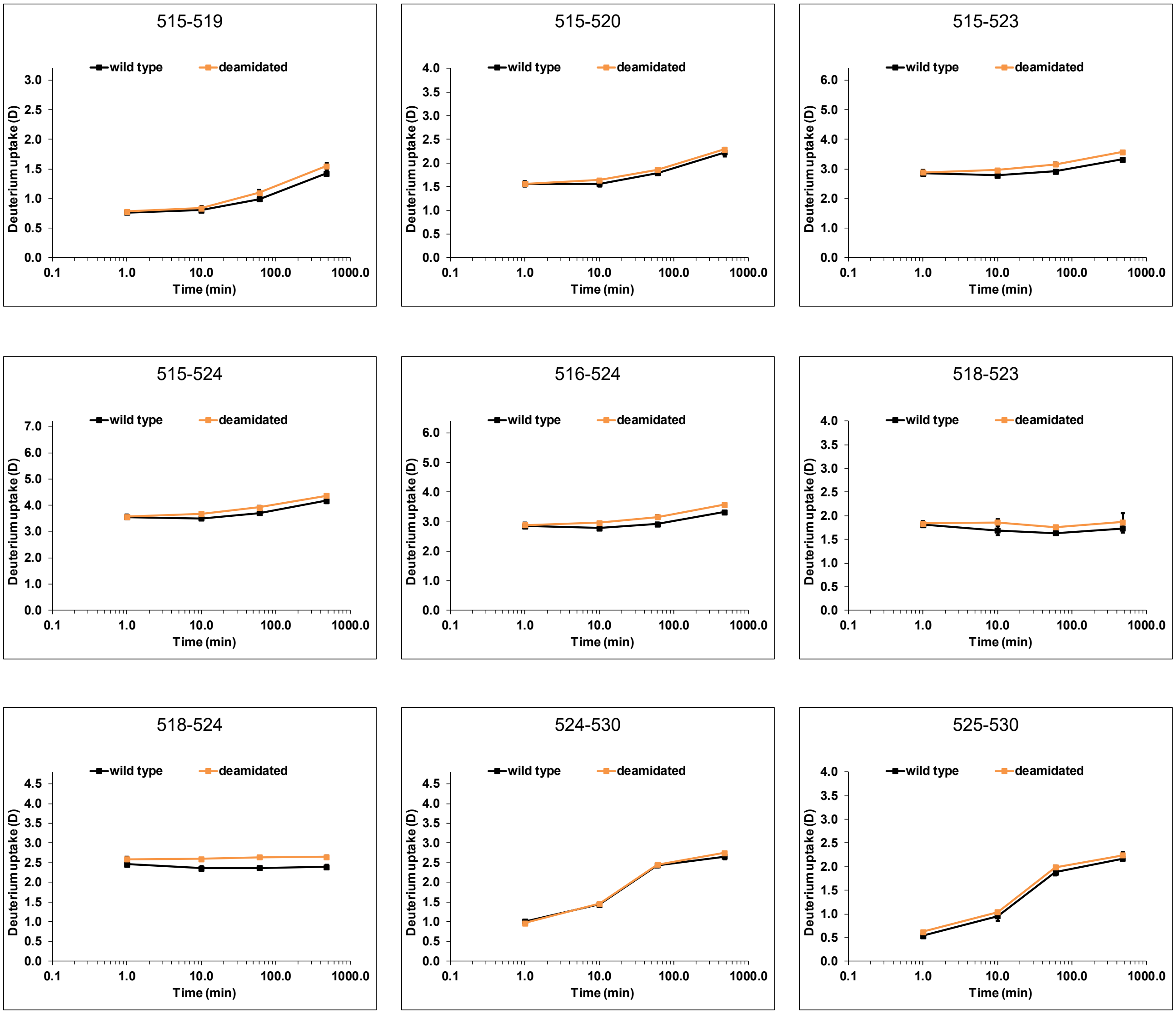

Figure S 33: continued

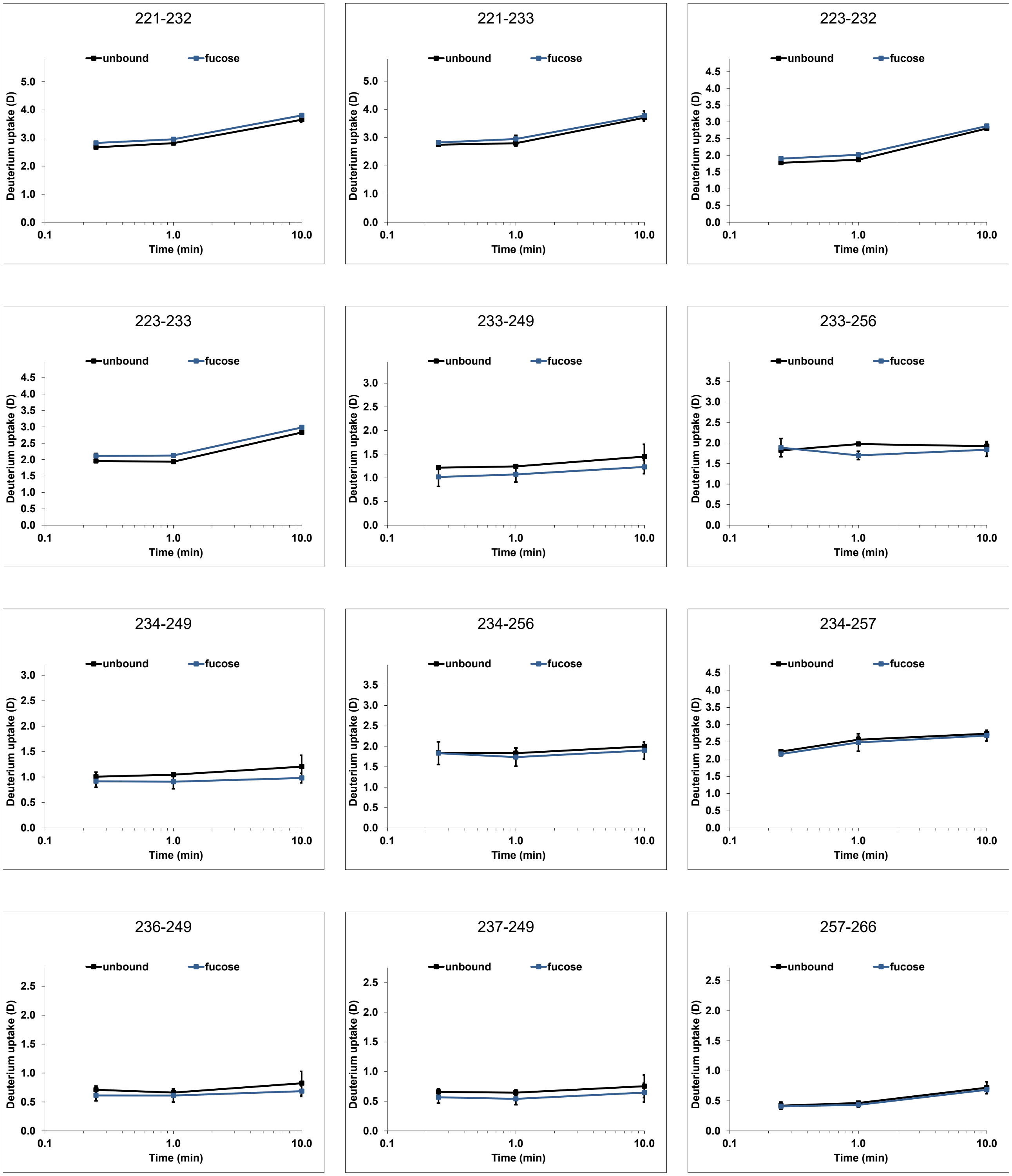

Figure S 34: Centroid deuterium uptake plots for partially deamidated Saga P dimers (black) with 100 mM fucose (blue) (dataset T10). All time points (15 s, 1 min and 10 min) were performed in triplicate. Error bars indicate the standard deviation (SD). This dataset did not show bimodality. Compare Figure S 12 and Table S 3 for assessment of statistically significant deuteration differences.

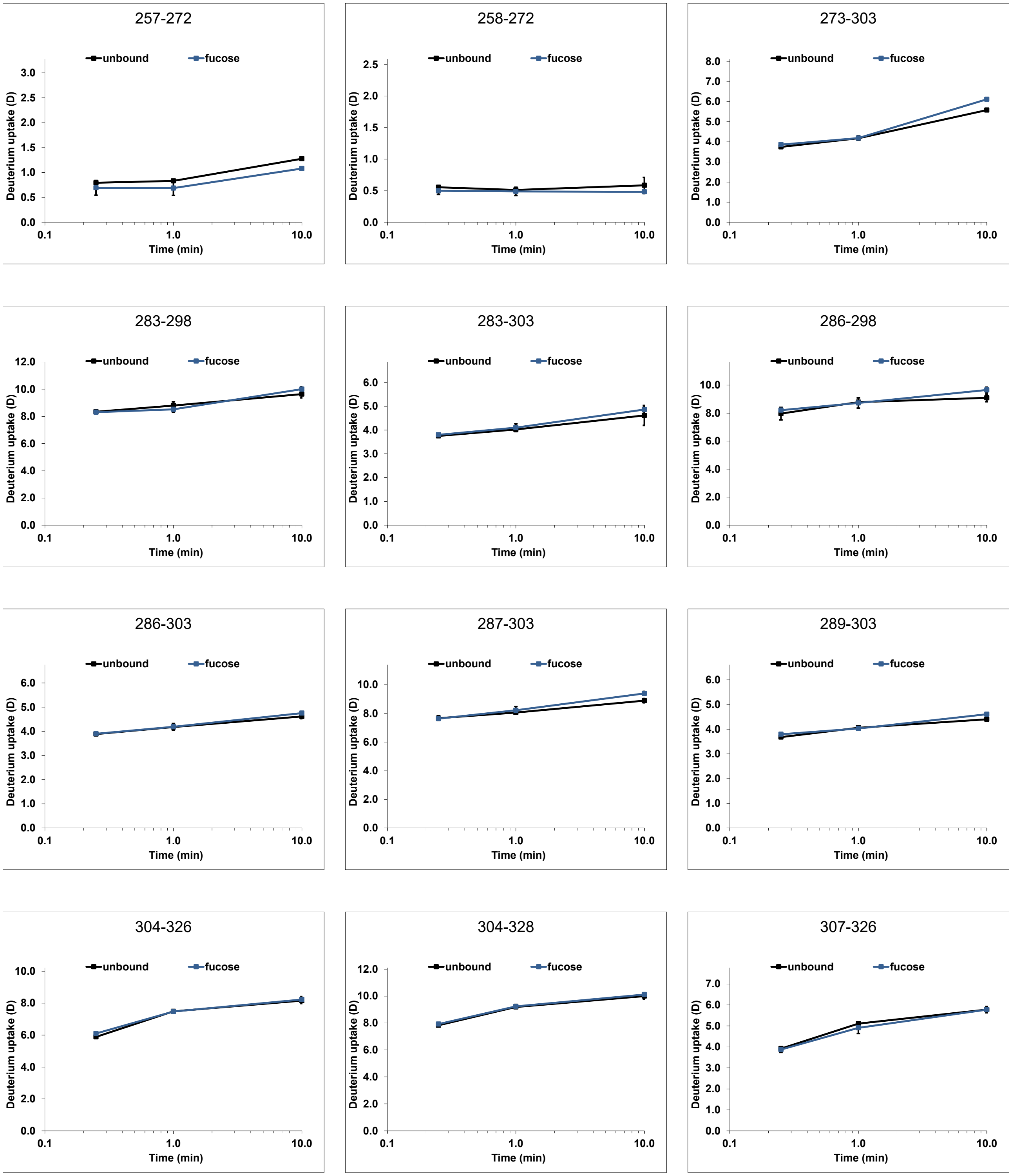

Figure S 34: continued

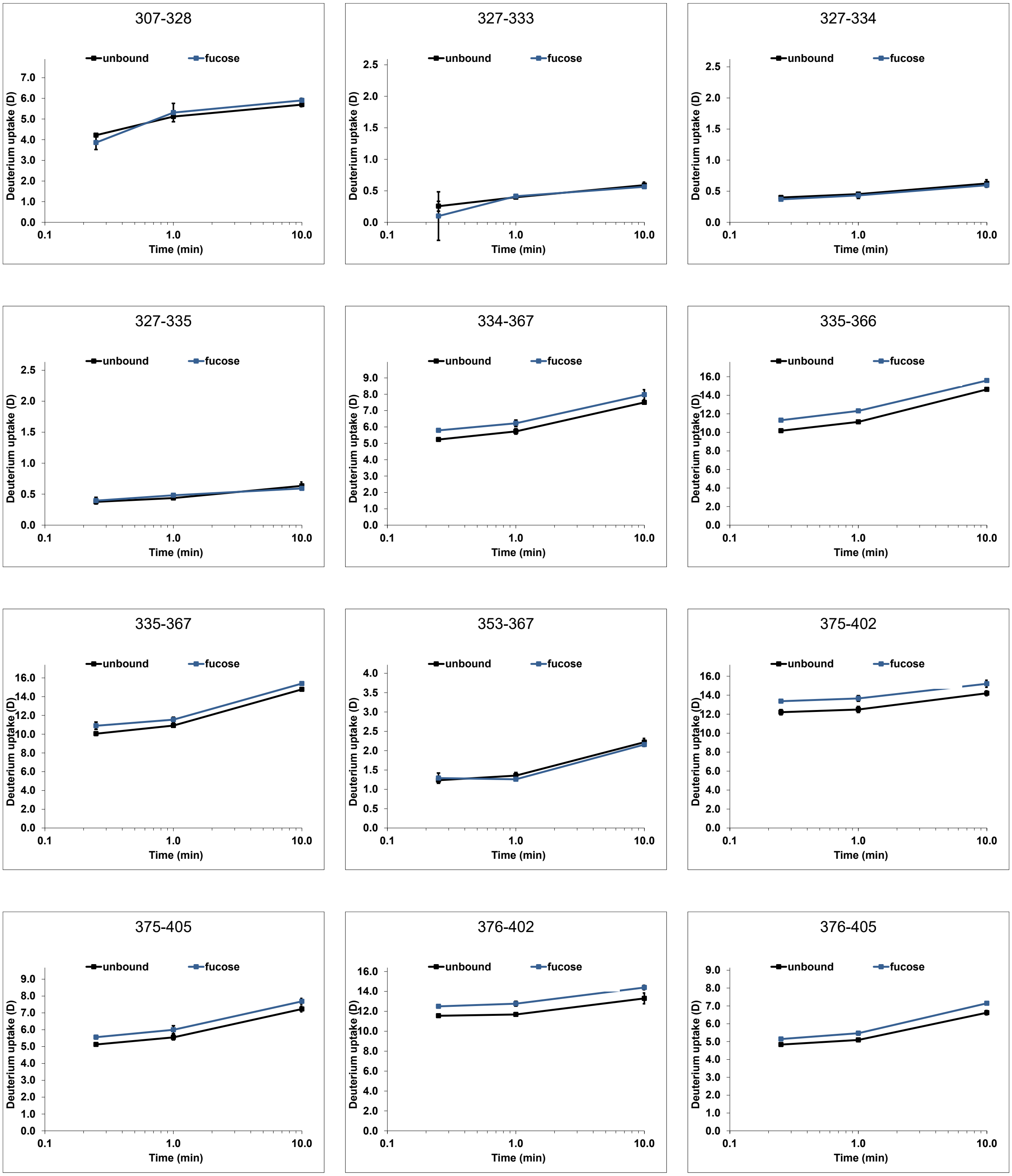

Figure S 34: continued

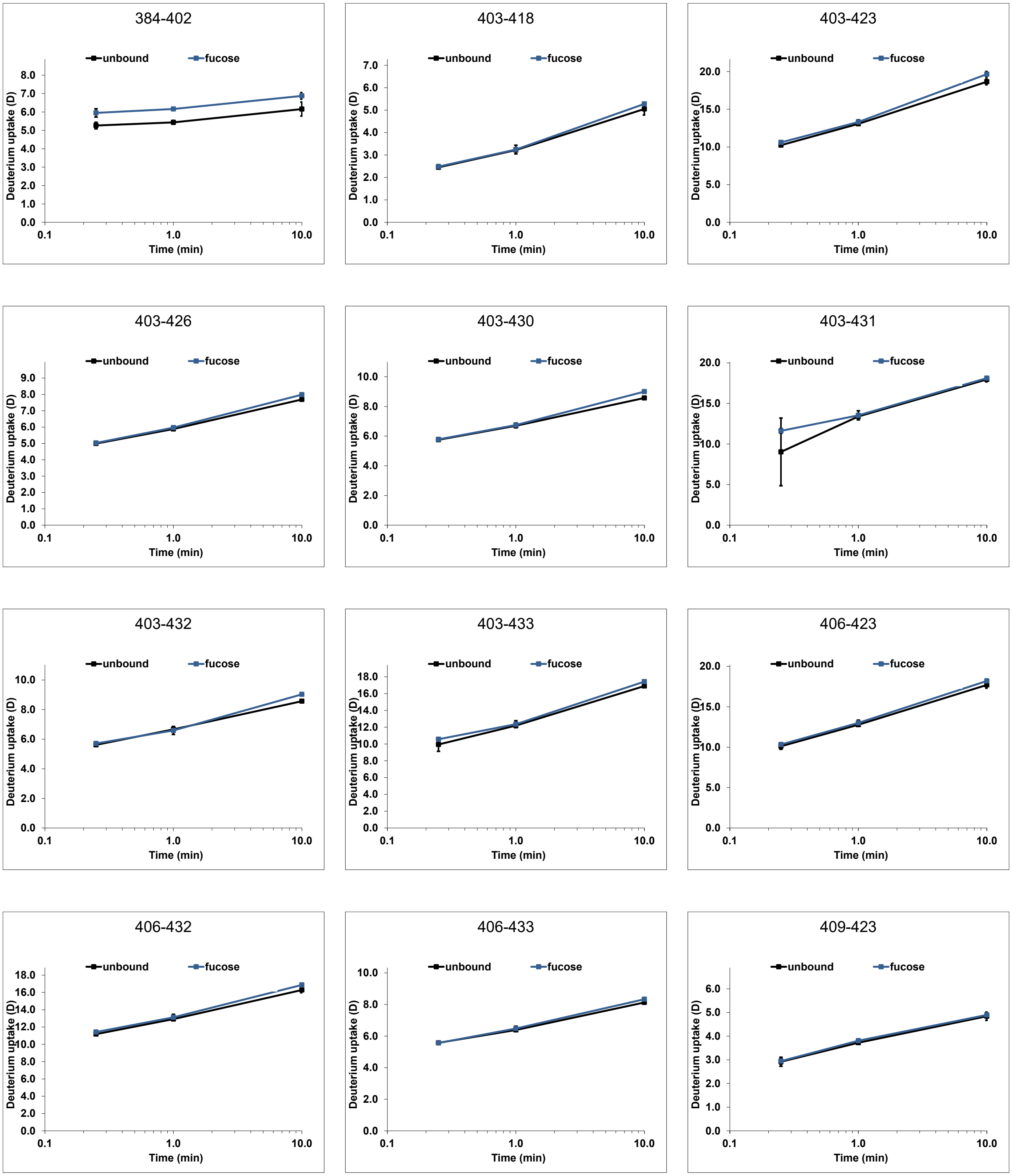

Figure S 34: continued

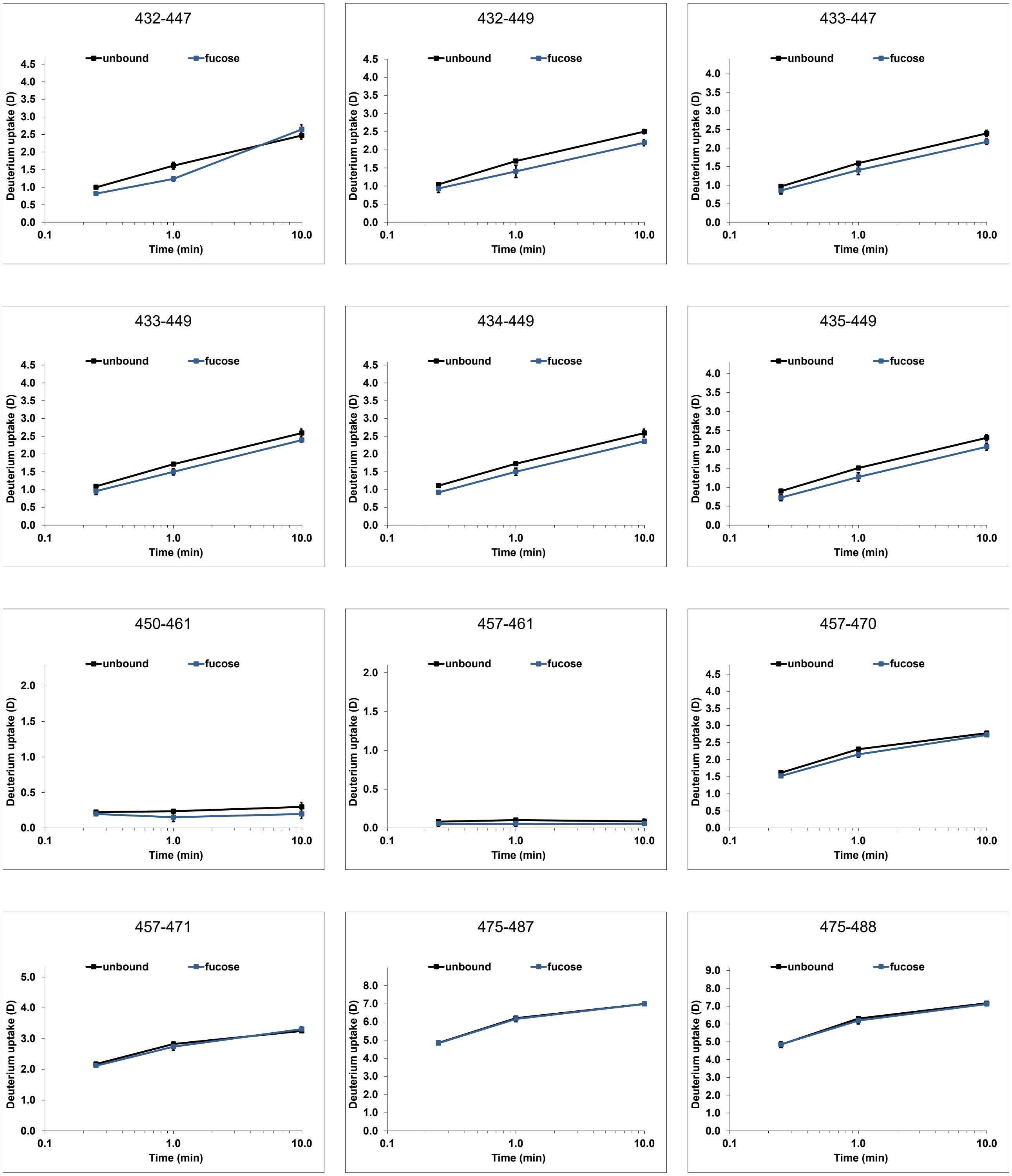

Figure S 34: continued

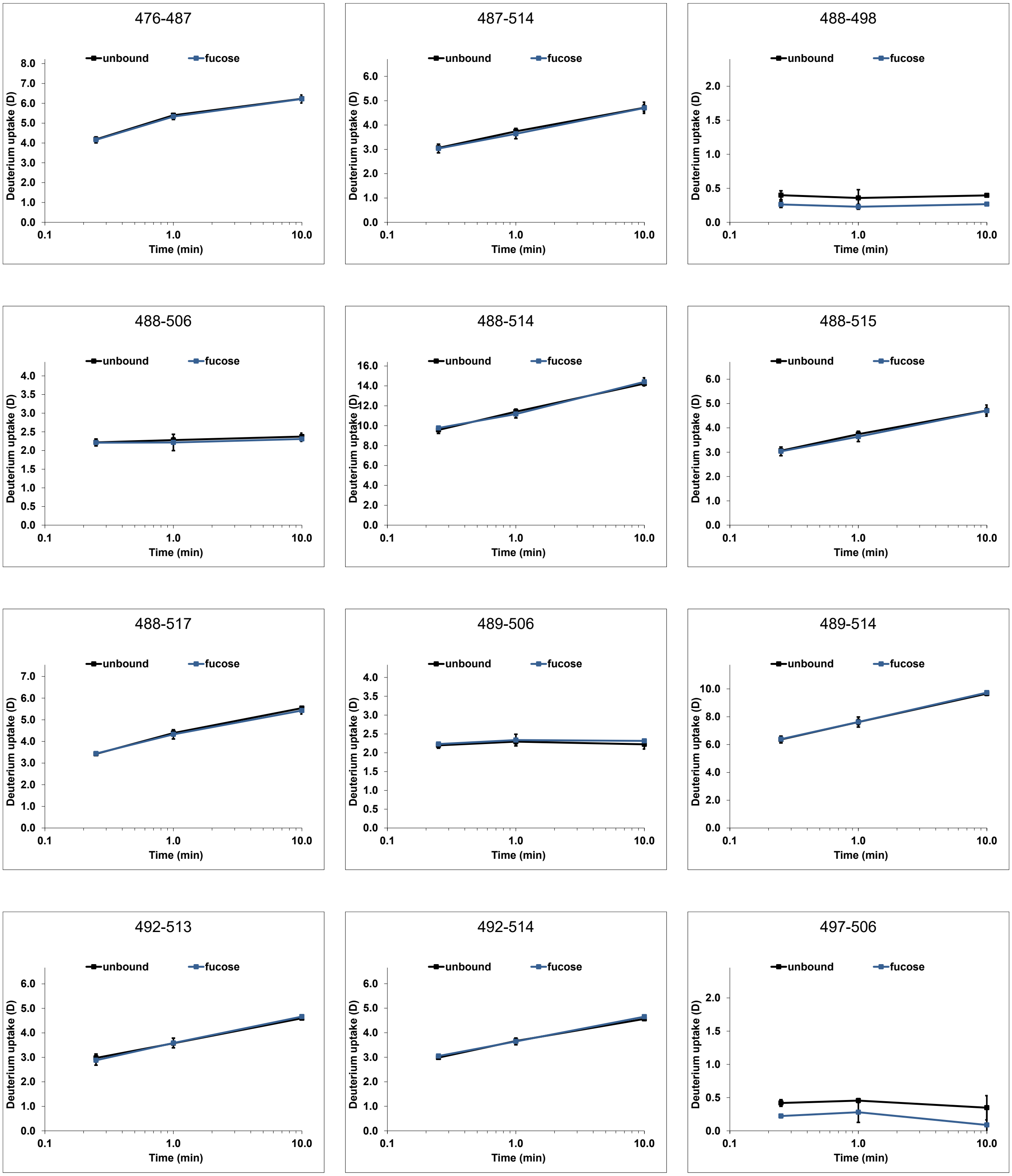
476-487
487-514
488-498
488-506
488-514
488-515
488-517
489-506
489-514
492-513
492-514
497-506
unbound
fucose
Deuterium uptake (D)
Time (min)

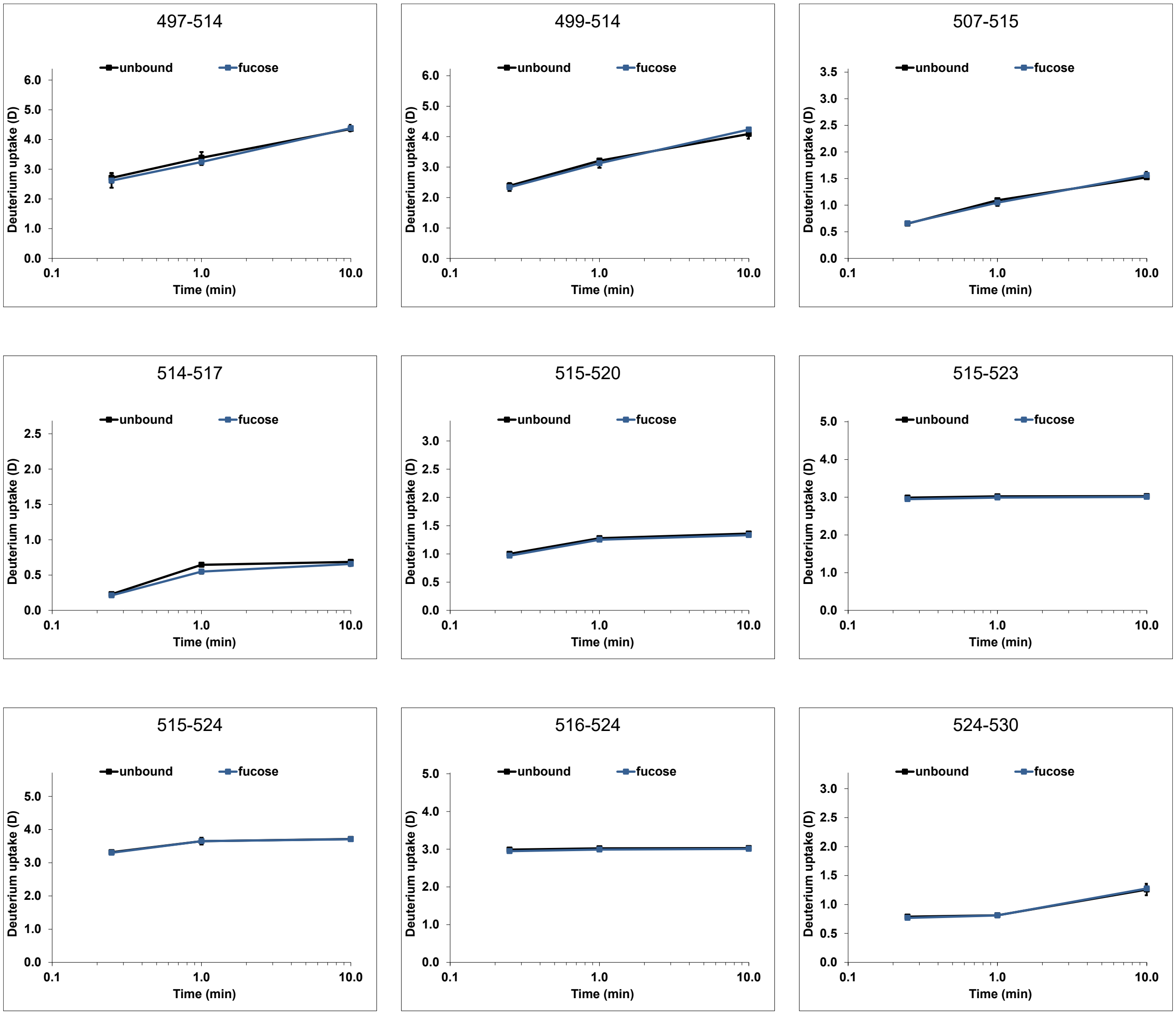

Figure S 34: continued
